# Transition to active learning in rural Nepal: an adaptable and scalable curriculum development model

**DOI:** 10.1186/s12909-019-1492-3

**Published:** 2019-02-20

**Authors:** Stephen Mehanni, Lena Wong, Bibhav Acharya, Pawan Agrawal, Anu Aryal, Madhur Basnet, David Citrin, Binod Dangal, Grace Deukmedjian, Santosh Kumar Dhungana, Bikash Gauchan, Tula Krishna Gupta, Scott Halliday, S. P. Kalaunee, Uday Kshatriya, Anirudh Kumar, Duncan Maru, Sheela Maru, Viet Nguyen, Jhalak Sharma Paudel, Pragya Rimal, Marwa Saleh, Ryan Schwarz, Sikhar Bahadur Swar, Aradhana Thapa, Aparna Tiwari, Rebecca White, Wan-Ju Wu, Dan Schwarz

**Affiliations:** 1 Possible, Kathmandu, Nepal; 2 Health Equity Action Leadership Initiative, University of California, San Francisco, San Francisco, CA USA; 3 Gallup Indian Medical Center, Gallup, NM USA; 4 Tuba City Regional Health Care, Tuba City, AZ USA; 5 Department of Psychiatry, University of California San Francisco, San Francisco, CA USA; 6 Department of Psychiatry, B.P. Koirala Institute of Health Sciences, Dharan, Nepal; 7 Henry M. Jackson School of International Studies, University of Washington, Seattle, WA USA; 8 Department of Global Health, University of Washington, Seattle, WA USA; 9 Department of Anthropology, University of Washington, Seattle, WA USA; 10 Department of Pediatrics, Natividad Medical Center, Salinas, CA USA; 11 College of Business and Leadership, Eastern University, St. Davids, PA USA; 12 Arnhold Institute for Global Health, Icahn School of Medicine at Mount Sinai, New York, NY USA; 13 Department of Health Systems Design and Global Health, Icahn School of Medicine at Mount Sinai, New York, NY USA; 14 Department of Pediatrics, Icahn School of Medicine at Mount Sinai, New York, NY USA; 15 Department of Internal Medicine, Icahn School of Medicine at Mount Sinai, New York, NY USA; 16 Department of Obstetrics and Gynecology, Icahn School of Medicine at Mount Sinai, New York, NY USA; 17 National Health Training Center, Department of Health Services, Ministry of Health and Population, Kathmandu, Nepal; 18 Division of Global Health Equity, Department of Medicine, Brigham and Women’s Hospital, Boston, MA USA; 19 Department of Medicine, Harvard Medical School, Boston, MA USA; 20 Division of General Internal Medicine, Department of Medicine, Massachusetts General Hospital, Boston, MA USA; 21 Department of Psychiatry, Kathmandu Medical College, Kathmandu, Nepal; 22 Department of Obstetrics and Gynecology, Boston Medical Center, Boston, MA USA; 23 Department of Obstetrics and Gynecology, Boston University School of Medicine, Boston, MA USA; 24 Department of Medicine, Beth Israel Deaconess Medical Center, Boston, MA USA; 25 Ariadne Labs, Brigham and Women’s Hospital and Harvard T.H. Chan School of Public Health, Boston, MA USA

**Keywords:** Active learning, Continuing medical education, Curriculum development, Learners as teachers, Limited resource, Rural

## Abstract

**Background:**

Traditional medical education in much of the world has historically relied on passive learning. Although active learning has been in the medical education literature for decades, its incorporation into practice has been inconsistent. We describe and analyze the implementation of a multidisciplinary continuing medical education curriculum in a rural Nepali district hospital, for which a core objective was an organizational shift towards active learning.

**Methods:**

The intervention occurred in a district hospital in remote Nepal, staffed primarily by mid-level providers. Before the intervention, education sessions included traditional didactics. We conducted a mixed-methods needs assessment to determine the content and educational strategies for a revised curriculum. Our goal was to develop an effective, relevant, and acceptable curriculum, which could facilitate active learning. As part of the intervention, physicians acted as both learners and teachers by creating and delivering lectures. Presenters used lecture templates to prioritize clarity, relevance, and audience engagement, including discussion questions and clinical cases. Two 6-month curricular cycles were completed during the study period. Daily lecture evaluations assessed ease of understanding, relevance, clinical practice change, and participation. Periodic lecture audits recorded learner talk-time, the proportion of lecture time during which learners were talking, as a surrogate for active learning. Feedback from evaluation and audit results was provided to presenters, and pre- and post-curriculum knowledge assessment exams were conducted.

**Results:**

Lecture audits showed a significant increase in learner talk-time, from 14% at baseline to 30% between months 3–6, maintained at 31% through months 6–12. Lecture evaluations demonstrated satisfaction with the curriculum. Pre- and post-curriculum knowledge assessment scores improved from 50 to 64% (difference 13.3% ± 4.5%, *p* = 0.006). As an outcome for the measure of organizational change, the curriculum was replicated at an additional clinical site.

**Conclusion:**

We demonstrate that active learning can be facilitated by implementing a new educational strategy. Lecture audits proved useful for internal program improvement. The components of the intervention which are transferable to other rural settings include the use of learners as teachers, lecture templates, and provision of immediate feedback. This curricular model could be adapted to similar settings in Nepal, and globally.

**Electronic supplementary material:**

The online version of this article (10.1186/s12909-019-1492-3) contains supplementary material, which is available to authorized users.

## Background

Robust continuing medical education (CME) programs are common in academic and urban centers in high-income countries, though are often lacking in rural or limited resource settings. Continuing education is heralded as an evidence-based strategy for improving healthcare worker retention in rural areas [1]. This is pertinent in low- and middle-income countries such as Nepal, which struggle with a healthcare worker shortage, especially in rural areas [2]. Recent Nepali medical graduates have been hesitant to work in rural posts, in part due to a lack of professional development opportunities [3, 4], and for junior clinicians, working in rural posts require additional training [5, 6].

Academic medical institutions in high-income countries are experiencing a surge in medical education innovations. However, there is a paucity of medical education literature from low-income countries. Using a framework for classifying medical education research [7], published studies from low-income countries are often descriptive in nature [8, 9], though several clarification studies exist [10, 11].

In the authors’ experience, passive learning is often the default in Nepali medical education, with didactics, rote memorization, and fact-based, rather than student-centered learning. Deficits in medical education are magnified in rural Nepal, where healthcare worker shortages are common [2]. However, there is interest among both educators and learners in realizing a shift towards more effective methods of education.

Active learning has varying definitions, but its core elements include student activity and engagement [12]. We focus on active learning in our intervention for several reasons. There is robust evidence that active learning strategies improve educational outcomes with regards to knowledge retention, thinking and writing skills, conceptual understanding, and knowledge transfer [12–15]. The evidence is especially robust in science, engineering, and mathematics [12–14]. Feasible strategies exist for adapting traditional lectures, in which information is presented to students passively, towards active learning. These strategies can be adopted by medical faculty who are not familiar with active learning pedagogies [16]. In addition, skill-based competencies, such as those required in the practice of medicine, can be effectively taught with learner participation [17].

A question that has not been addressed to our knowledge is the extent to which the implementation of active learning strategies can transform the learning climate in a limited resource setting. Despite the dominance of didactic lectures in Nepali medical education, evidence from other settings has shown that learning preferences can be molded over time through curricular change [18]. Teaching behavior in medical education has also been shown to be modifiable through strategies such as experiential learning, feedback, and the fostering of effective relationships [19]. In addition, strategies to facilitate active learning in a multidisciplinary CME curriculum could help to open communication flows between physicians, mid-level providers, and nurses. This is an important consideration, as many medical errors are associated with communication failures across power hierarchies [20]. The question this study seeks to address is: can educational interventions within a curriculum development initiative facilitate a transition towards active learning in rural Nepal?

In this paper, we describe and analyze a multifaceted intervention to facilitate active learning, through the implementation of a multidisciplinary CME curriculum in a rural Nepali public sector healthcare facility. We explore the use and limitations of various conceptual models as they apply to this unique setting. We frame the active learning intervention around Ericsson’s theory of expertise [21, 22]. We describe the curriculum development using Kern’s six-step model, including problem identification, needs assessment, goals and objectives, educational strategies, implementation, and evaluation and feedback [17]. We use a modified version of Kirkpatrick’s hierarchy of educational evidence to frame our evaluation [23, 24]. We discuss potential applications of our experience in other limited resource settings.

## Methods

### Ethics and consent

Written and verbal consent was obtained in Nepali for healthcare workers participating in the program evaluation. The study protocol was approved by the Nepal Health Research Council, registration number 472/2017.

### Study setting

Bayalpata Hospital (BH) is a public-sector hospital in the remote district of Achham, Nepal. The hospital is managed in partnership with Nepal’s Ministry of Health and Population by the nonprofit healthcare organization *Possible*. As of 2018, BH is staffed by ten community medical assistants (CMAs), 14 health assistants (HAs), and 26 nurses and auxiliary nurse midwives (ANMs). There are additionally seven staff physicians with Bachelor of Medicine, Bachelor of Surgery (MBBS) degrees, as well as three physicians who have completed a Doctor of Medicine, General Practitioner degree (MD-GP).

Professional training is 18 months of post-secondary medical education for ANMs and CMAs, and three years for HAs. Training for mid-level providers (HAs and CMAs) occurs primarily through didactic instruction of disease-specific guidelines for assessment and treatment, with a limited basic science foundation. Mid-level providers are tasked with diagnosing and treating a range of common medical conditions. Mid-level providers provide direct patient care in outpatient clinics, emergency departments, and inpatient wards. In addition, they perform minor procedures, and have prescribing privileges. At BH, mid-level providers receive direct supervision from staff physicians via daily rounds in the inpatient and emergency departments. In the outpatient clinics, mid-level providers act more independently, with physician consultation upon request only. Among mid-level providers, there is wide variation in ability to diagnose and treat common medical and surgical conditions.

The hospital cares for approximately 100,000 patients annually. There is a high burden of infectious diseases at BH including tuberculosis, childhood pneumonia, diarrheal illness, and HIV. There is an increasing burden of non-communicable diseases including chronic respiratory disease, heart failure, diabetes, hypertension, stroke, and mental illness.

### Problem identification and general needs assessment

Prior to the intervention, CMAs, HAs, physicians, ANMs, and nurses all attended a joint CME session for 45 min each morning, six days per week. Four sessions weekly were dedicated to didactic lectures, one session for a case report, and one session for a morbidity & mortality conference [25]. Lecture topics were decided on a weekly basis and assigned to staff physicians who had approximately two to three days to develop a lecture prior to presenting. The lecture topics were often quite broad. There was an expectation that lectures be targeted to mid-level providers, but with minimal guidance as to how to create a presentation that effectively achieved this goal. Thus, lectures often had information that was not relevant to the clinical setting and was not delivered or organized in such a way as to promote meaningful learning or retention. Lectures were delivered as traditional didactics, meaning a staff physician would deliver information compiled on a slide set, with minimal participation or discussion by learners.

The lecture topics had not previously been organized into a rational curriculum. An important exception is the implementation of a curriculum that focused on mental illness and was developed as part of a separate implementation research study [6, 26, 27]. Those education interventions were conducted every quarter and delivered by visiting psychiatrists. This paper focuses on the regular curricular interventions developed and delivered locally by on-site clinicians.

### Targeted needs assessment

A mixed-methods needs assessment, loosely based on a previously published approach [28], was conducted between February and June of 2016. The needs assessment was done to inform the design of the curriculum intervention and included brief healthcare worker surveys developed for this study, direct observation, and analysis of the local disease burden. Healthcare workers indicated the following as positive factors for their learning: discussions, clinical cases, questions, compulsory attendance, and testing. Additionally, they noted they would prefer information on national guidelines and common complaints. A lecture review showed deficiencies in topics relevant to the local setting. Based on direct observation, and informal quizzes conducted during CME time, medical leadership determined that providers had difficulty with diagnosis and management of chronic non-communicable diseases and psychiatric conditions. Finally, we reviewed Nepal health statistics from the Nepal Ministry of Health and Population and the World Health Organization, in addition to the most common diagnoses at BH, in order to develop an outline for a cohesive curriculum (Additional file 1).

### Goals and objectives

Our goal was to develop an effective, relevant, and acceptable curriculum, targeted to mid-level providers, with content created and delivered by Nepali physicians, in order to improve clinical care and catalyze a transformation towards active learning. This goal was directly informed by the targeted needs assessment, as described above. Objectives included: 1) improving clinical knowledge, as measured by an increase in score on a multiple-choice knowledge-assessment exam; 2) achieving acceptability and satisfaction for learners, as measured by lecture evaluation results; 3) achieving impact for the regional medical education system, as determined by the decision of whether to expand the curriculum to an additional clinical site; and 4) increasing learner participation, as measured by learner talk-time during didactics.

### Educational strategies

Our intervention focused on the didactic component of the curriculum. CME course content was structured to focus on the diseases and problems that represented the largest burdens for our patient population, and the largest knowledge deficits for our learners. The content was organized into system blocks (e.g. orthopedic, pulmonary, cardiovascular), with lecture topics organized by problem (e.g. “Approach to dyspnea” and “Diabetes in the clinic”). We created detailed and structured PowerPoint templates according to evidence on lecture effectiveness and cognitive load theory [29–31], to focus on clarity, visibility, relevance, simplicity, and audience engagement. Templates guided presenters to focus on common diagnoses and problems, and towards use of educational objectives, clinical cases, discussion questions, visual learning aids, repetition, and key learning points (Additional files 2 and 3). In this way, concepts of active learning and case-based learning could be incorporated into the lectures [16]. The presenters were encouraged to focus their content to the median level of training, or the mid-level provider. However, they were encouraged to include relevant material in each didactic to maintain engagement for learners at other levels. Templates encouraged lecturers to use images when possible and to avoid PowerPoint slides with greater than 33 words. This is attendant to cognitive load theory, which holds that when an individual’s attention is divided between spoken and written words, learning is less effective [29–31].

Staff physicians, who normally attended CME as learners, also served as teachers in this model. Physicians developed and delivered course content in Nepali, based on templates on a rotating basis. Individual staff physicians delivered approximately one to two lectures monthly. In this way, deep learning occurred for the individual who prepared and delivered the lecture, and practical knowledge could be obtained by other participants.

### Implementation

Support from the hospital’s leadership team was in place prior to implementation. There was protected time each morning for CME, with mandatory attendance. The Medical Director and the Director of Medical Education at BH, in conjunction with university-based clinicians working with *Possible*, oversaw the curriculum development, including its course structure and framework.

A CME session was scheduled to communicate the rationale, goals, and objectives of the new curriculum, and to deliver a pre-curriculum knowledge assessment exam. An additional introductory CME session introduced learners and clinicians to the concept of active learning.

Clinicians with backgrounds in medical education generated templates for a six-month rotating curriculum, which was chosen to account for average medical staff turnover. The templates were distributed to staff physicians on a rolling basis, approximately one to two weeks prior to their delivering a lecture. Staff physicians would then generate lecture content based on the template, and deliver the lectures during scheduled didactic time. To describe the assumptions and rationale underlying the curriculum intervention, we shared a Theory of Change map (Additional file 4) with the hospital team.

To allow for further refinement during the next curricular cycle, all finalized lectures were stored in a repository. Future program expansion could include implementation of the curriculum at another clinical site managed by *Possible*. During the study period, two 6-month curricular cycles were completed.

### Evaluation and feedback

Based on Kirkpatrick’s hierarchy, which has been adapted to medical education [23, 24], this evaluation design included direct or indirect measurements of learner participation, attitudes and perceptions, knowledge acquisition, behavior changes, and organizational changes (Table 1). Changes to patient outcomes were not measured. While we acknowledge Kirkpatrick’s framework has been criticized for its reliance on implicit assumptions [32], we felt it was appropriate for this study because of the relatively simple instructional design and short-term endpoints.

**Table 1:** Components of the curriculum evaluation, organized by Kirkpatrick’s hierarchy

Kirkpatrick’s Hierarchy	Evaluation Tool	Educational Objective
*Healthcare outcomes or organizational changes*	Curricular expansion	Expansion of curriculum to an additional clinical site
*Behavior changes*	Lecture evaluation	Self-reported change in clinical practice patterns
*Knowledge acquisition*	Knowledge assessment exam	Improvement in score from pre- to post-curriculum
*Attitudes and perceptions*	Lecture evaluation	Self-reported ease of understanding and relevance
*Participation*	Lecture audit	Increase in learner talk-time during lectures
	Lecture evaluation	Self-reported participation during lectures

We developed an English-language multiple-choice knowledge assessment exam. Nepali medical leadership and U.S. physicians developed one to two multiple-choice questions from each lecture. Questions were based on key points and learning objectives from lectures. All questions were pooled, reviewed, and edited by medical team leadership via an online project management system, to ensure best practices were used [33]. From the total pool of questions, 33 were selected for the exam, to ensure participants would have time to complete the exam during a standard 45-min CME session. Questions were intentionally drawn from all blocks of the curriculum. The exam was administered to mid-level providers and physicians only, prior to and following the first curricular cycle. Exam results were stratified by level of training, to allow for internal program evaluation. In this way we could assess the extent to which exam scores changed for physicians vs mid-level providers, to determine whether we were achieving our goal of targeting the curriculum to mid-level providers. After ensuring results were normally distributed, a two-sample t-test was conducted to determine significance between differences in exam scores. All statistical analyses were performed using Stata software, version 13 (StataCorp LLC).

Paper-based, anonymous lecture evaluations were completed by learners daily in Nepali, immediately following each lecture (Additional file 5). Two questions focused on whether the lecture was easy to understand, and whether it was relevant to their work. These questions utilized a 4-point Likert scale. Two questions, inquiring whether the learner could identify a clinical practice change, and whether they participated during the lecture, elicited ‘yes’ or ‘no’ responses. Evaluations intentionally contained self-regulating questions, encouraging trainees to reflect on the learning process and its translation to clinical care. This was done to harness the survey effect, which posits that these types of questions can prime subjects to modify their attitudes and priorities, which in turn can enhance training effectiveness and improve learning [34–36].

Additionally, lecture audits were performed in real-time using convenience sampling of lectures by one of two clinicians (SM and LW). Auditors observed lectures in real-time, using a stopwatch to record the total lecture time, as well as the learner talk-time. Audits were performed discreetly, and participants were not aware which lectures were being audited. Definitions for talk-time were agreed upon by auditors in advance. Learner talk-time was measured as the proportion of time in which learners were speaking (asking a question, responding to a question, or participating in discussion), or in which the presenter awaited a response to a question. This metric was used as a proxy for the incorporation of active learning strategies into lectures. Student-to-teacher talk-time ratios have been correlated previously with lecture effectiveness, high performing classes, and increased learner satisfaction [37–40].

Presenters received immediate feedback on their educational strategies from one of two clinicians (SM and LW). In addition, results from lecture evaluations and audits were fed back to presenters on the same day. This strategy was based on Ericcson’s theory of expertise, which posits that immediate feedback is a core component of behavior change and expertise development [21]. It also aligns with instructional strategies which have been shown to be effective, including opportunities for practice and feedback, and reinforcement techniques [41]. Aggregated results from knowledge assessment exams were shared with healthcare workers. Curricular feedback received from lecture evaluations was incorporated into iterative curricular design. For example, based on early feedback from participants, review sessions with clinical questions were held at the end of each system block.

## Results

**Fig. 1:**
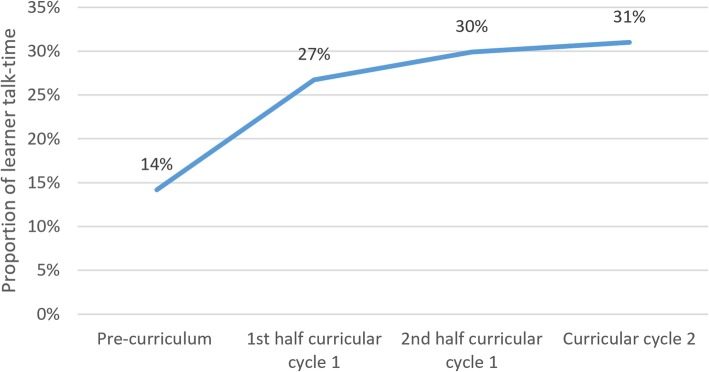
Learner talk-time by curricular cycle. The proportion of total lecture time during which learners talked, as opposed to presenters, is shown here. Results are subdivided by the curricular cycles during which lecture audits took place. Pre-curriculum audits (*n* = 7) took place during lectures prior to the initiation of the 6-month repeating curriculum. 1st half of curricular cycle 1 audits (*n* = 12) took place during the first three months of cycle 1. 2nd half of curricular cycle 1 audits (*n* = 20) took place during the second three months of cycle 1. Curricular cycle 2 audits (*n* = 10) took place during the second cycle of this 6-month repeating curriculum

To determine if learner talk-time increased over the course of the intervention, selected lectures were audited, and classified into four groups: pre-curriculum (*n* = 7), 1st half of curricular cycle 1 (months 0–3; *n* = 12), 2nd half of curricular cycle 1 (months 3–6; *n* = 20), and curricular cycle 2 (months 6–12; *n* = 10). Learner talk-time increased from a pre-curriculum baseline of 14, to 30% at the second half of the curricular cycle 1. This improvement was sustained through curricular cycle 2 (Fig. 1). After determining the data were normally distributed, we conducted a one-way ANOVA, which demonstrated the difference in talk-time to be statistically significant between groups (*F*(3,45) 4.07, *p* = .012).

**Fig. 2:**
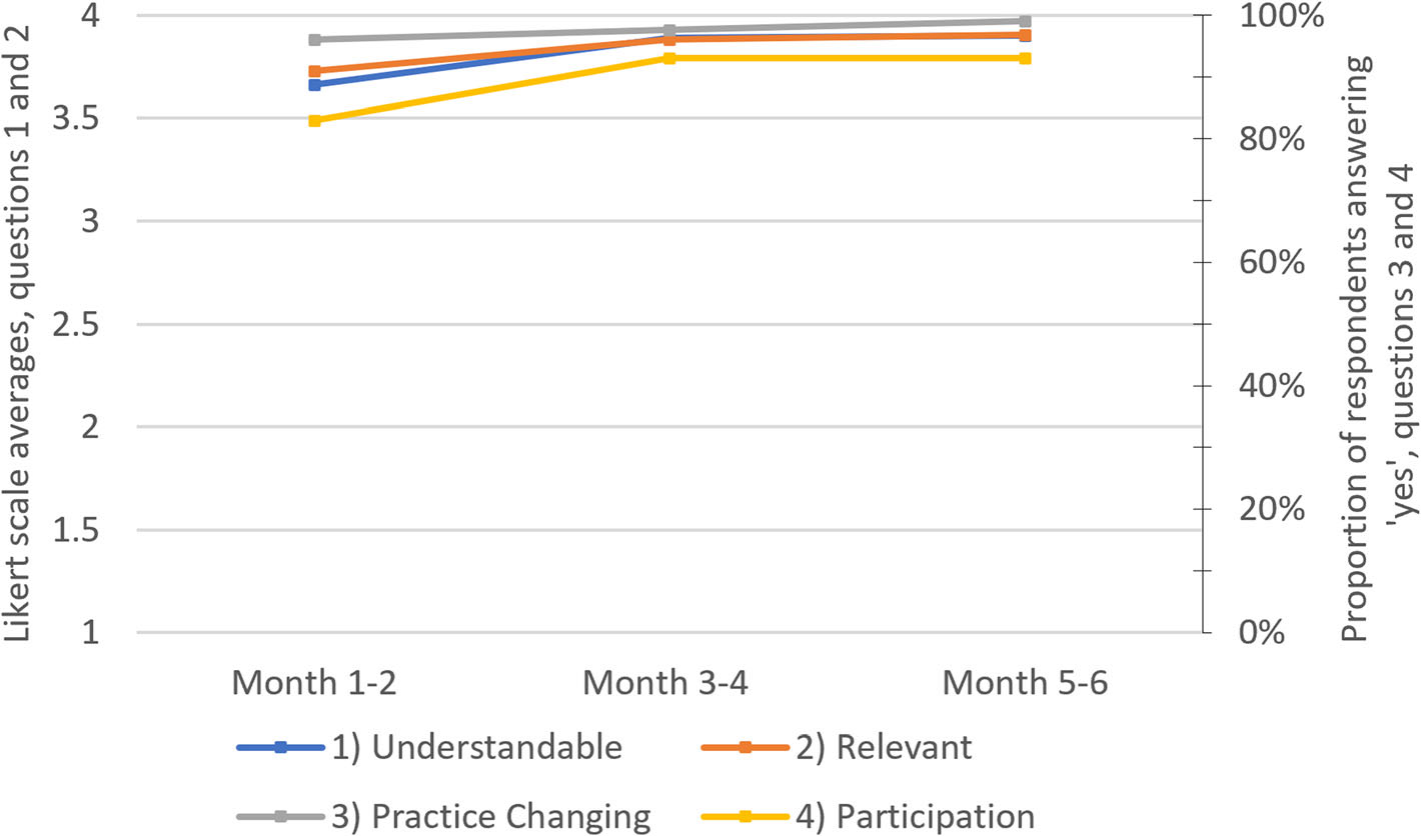
Lecture evaluations over time, curricular cycle 1. Questions 1 and 2 utilized a four-point Likert scale (1 = strongly disagree, 2 = disagree, 3 = agree, 4 = strongly agree). Likert scale scores were aggregated by question and subdivided into two-month increments during curricular cycle 1. Questions 3 and 4 elicited ‘yes’ or ‘no’ responses. The proportion of respondents answering ‘yes’ was aggregated by question and subdivided into two-month increments during curricular cycle 1. See Additional file 5 for the specific evaluation questions

Lecture evaluation scores were high for the duration of the curriculum (Fig. 2). There was a slight improvement in evaluation scores after the first two months, with scores later plateauing. Pre-curriculum lecture evaluations are not available for comparison. There was a slight drop in evaluations received over time, with an average of 26.2 evaluations per lecture for months 1–2, compared to 22.1 evaluations per lecture for months 5–6. We are not able to calculate the more meaningful evaluation response rates, as we do not have reliable denominator data for the total number of staff in attendance at each lecture. This number depended on leave time and other variables.

Pre- and post-curriculum knowledge assessment scores improved from 50 to 64%. A two-sample t-test demonstrated the difference in exam scores to be significant (13.3% ± 4.5%, *p* = 0.006). This difference remained significant when restricted to mid-level providers (8.7% ± 4.1%, *p* = 0.047). Of 34 total mid-level providers and physicians on staff, 18 completed the pre-curriculum exam, and 17 completed the post-curriculum exam. Providers who did not complete the exams were either on leave, or on duty during the examination time.

As an outcome for the measure of organizational change, the curriculum was replicated at an additional clinical site managed by *Possible* and the Ministry of Health and Population: Charikot Primary Health Center in Dolakha District, Nepal.

## Discussion

Here we describe a multicomponent curricular intervention in rural Nepal, with one objective being a transition towards active learning. As mentioned, a targeted needs assessment informed our objectives and strategies, and indicated learner openness to active learning strategies such as questions, discussions, and clinical cases. We attempted to harness simple strategies such as immediate feedback and the survey effect to increase active learning during lectures, as measured by learner talk-time.

### Lessons learned

Our results broadly support the premise that a curriculum developed using educational theories can facilitate increased learner participation. We know from prior research that curricula themselves can mold students’ learning preferences [18]. A key finding from our intervention includes the acceptability and feasibility of a transition towards active learning. Ericsson’s theory of expertise provides insights into how the behavior change, for both the learners and teachers, was accomplished. For teachers, standardized PowerPoint templates made the incorporation of clinical cases, discussions, and audience questions the default option. Teachers received same-day, one on one feedback through lecture audit results, lecture evaluation results, and verbal discussion. Teachers also received implicit feedback during lectures, that learners were willing and able to participate. The ability to transform teaching behavior is in line with the education literature, which suggests educational interventions can lead to changes in teaching behavior, as reported by participants and detected by students [19]. Through the incorporation of lecture audits into our evaluation, we demonstrated change in teacher and learner behavior during an education intervention, sustained over time. Lecture audits are unlikely to be necessary in the maintenance or scaling phase of a curriculum, but rather can be useful for developing or refining the intervention.

As mentioned previously, during our targeted needs assessment, learners indicated they would prefer additional questions, discussions, and cases to be incorporated into lectures. However, it can be intimidating for mid-level providers or nurses to speak in a traditionally hierarchical medical system. Through the lecture evaluation, learners were primed to realize that their participation was valued and expected. Indeed, both self-reported participation, and the proportion of time learners were participating in discussions increased over the course of the curriculum. Facilitating dialogue between physicians, nurses, and mid-level providers may have other effects, such as reducing medical errors in traditionally hierarchical systems [20]. While unmeasured in this study, it is plausible that this curricular intervention led to positive effects on inter-team communication.

Learners were successfully tasked with teaching roles, to allow current and future Nepali medical educators to gain comfort with active learning techniques, which could help with the sustainability of change. While additional effort was required to facilitate active learning, follow-up results during the second curricular cycle suggest these changes can be sustained over time. Additionally, a large amount of curricular content could be generated in a short period of time through the use of lecture templates, because learners could then be tasked with creating lecture content.

### Limitations

Limitations of our study include weaknesses with the data generating processes. For example, surveys seek to measure an individual’s subjective preferences or feelings, but they are inherently subject to responder bias and survey fatigue. Our lecture evaluations initially showed slight improvements, but then plateaued. Initial improvements in evaluation scores could be explained by presenters modifying the content and style of presentations based on regular feedback from academic clinicians, and lecture evaluation results. Though survey fatigue, in which participants begin to feel ambivalence towards surveys with increasing use, may have played an additional role in the eventual high scores [42, 43]. The decreased number of evaluations received per lecture over time also suggest survey fatigue may have contributed. Though we cannot be certain of this conclusion without evaluation response rates. In this study, the surveys were designed to augment curricular effectiveness by harnessing the survey effect, though we are not able to measure whether this attempt was successful.

There were significant improvements in knowledge assessment exam scores from pre- to post-curriculum, but this improvement does not automatically translate to improved clinical outcomes or behavior change, neither of which were directly measured. It is worth noting the exam was not standardized, and for convenience, every question was given equal weight during scoring. Due to limitations in data availability during the curriculum evaluation stage, two-sample t-tests were used rather than paired t-tests. This allowed us to compare exam scores at the aggregate, but not the individual level. The pre- and post-curriculum exams were administered six months apart, but were identical, which may have affected the score improvements noted. Additionally, we did not have a control arm to determine how the new curriculum might have compared with an alternate curriculum. Furthermore, it is not possible to infer which components of the curriculum were effective based on exam results.

There is some theoretical and experimental evidence to suggest increased learner talk-time is associated with improved educational outcomes, and with active learning [37–40]. However, the use of learner talk-time in the lecture audit is only a crude surrogate for active learning. Lecture audits were performed by nonblinded coauthors SM and LW, rather than independent observers, and thus were subject to measurement bias. This was mitigated through pre-specified definitions of what constituted talk-time.

An additional limitation to this study is transferability. The study was designed to determine if the core components of the intervention are feasible and sustainable within a single setting, and to offer insights from its implementation and evaluation. Its core components are adaptable and likely feasible in similar settings in Nepal and globally, but further research will need to be done to verify transferability. Critical differences in learning culture exist across settings, and context-specific curricular modifications would be necessary if reproduced in other settings.

## Conclusions

We have designed and implemented a medical education intervention to transition towards active learning at a rural hospital in Nepal. This study shows this intervention is feasible and acceptable when embedded within a larger curriculum development initiative. Key lessons include harnessing learners as teachers, in conjunction with lecture templates and timely feedback, to overcome resource barriers to generating lecture content, and to ensure sustainability of change. The core components of this intervention are adaptable and may be valuable for similar settings within Nepal or globally.

## Additional files

Additional file 1: Curriculum outline, organized by system. (PDF 208 kb)

Additional file 2: Master PowerPoint template, used to generate topic-specific templates. (PPTX 419 kb)

Additional file 3: Sample topic-specific PowerPoint template: “Approach to Heart Failure”. (PPTX 548 kb)

Additional file 4: Theory of Change map for curriculum improvement. (PDF 171 kb)

Additional file 5: Lecture evaluation, in English and Nepali. (PDF 156 kb)

## Abbreviations

ANM: Auxiliary Nurse Midwife
BH: Bayalpata Hospital
CMA: Community Medical Assistant
CME: Continuing Medical Education
HA: Health Assistant
MBBS: Bachelor of Medicine, Bachelor of Surgery
MD-GP: Doctor of Medicine, General Practitioner
