# Supplementary material for: Transition to active learning in rural Nepal: an adaptable and scalable curriculum development model
Source: BMC Med Educ. 2019 Feb 20;19:61. doi: 10.1186/s12909-019-1492-3 (PMC6383231; doi:10.1186/s12909-019-1492-3)
Supplement: Supplementary file 1 — Curriculum outline, organized by system. (PDF 208 kb) [file 12909_2019_1492_MOESM1_ESM.pdf]

Bayalpata Hospital Continuing Medical Education Curriculum Outline

Obstetrics & Gynecology

1. Contraceptive counseling
2. Normal antenatal care
3. Abortion
4. Pregnancy complications
5. Labor and childbirth complications
6. Postpartum complications
7. Lower abdominal pain in female
8. Abnormal uterine bleeding and dysmenorrhea

Infectious Disease

1. Approach to fever
2. Sepsis
3. Pulmonary Tuberculosis
4. Extrapulmonary Tuberculosis and Visceral Leishmaniasis
5. Human Immunodeficiency Virus
6. Leprosy
7. Rational antibiotic prescribing

Gastroenterology

1. Abdominal Emergencies
2. Abdominal Pain
3. Acid Peptic Disorders
4. Approach to Gastrointestinal Bleeding
5. Pediatric Gastroenterology Cases
6. Gastrointestinal Parasites

Orthopedics & Musculoskeletal

1. Principles of fractures
2. Lower extremity trauma I
3. Lower extremity trauma II
4. Upper extremity trauma
5. Non-traumatic joint pain
6. Snake bite

Cardiovascular

1. Heart failure
2. Approach to chest pain
3. Hypertension and hypertensive emergency
4. Electrocardiogram I
5. Electrocardiogram II

6. Electrocardiogram Cases

Pulmonary

1. Approach to cough
2. Approach to dyspnea
3. Chronic respiratory diseases
4. Lower respiratory infection and pleural effusion
5. Reading Chest x-rays

Neurology

1. Approach to Headache
2. Meningitis
3. Neurologic Deficits
4. Evaluation of Syncope
5. Seizures

Pediatrics *(note: much of pediatrics is covered in organ-specific topics)*

1. Newborn care
2. Neonatal sepsis
3. Severe acute malnutrition

Head, Eyes, Ears, Nose and Throat

1. Ear pain/ discharge
2. Eye pain/ discharge
3. Throat and sinus problems

Dermatology

1. Skin Disorders I
2. Skin Disorders II
3. Burns

Endocrinology

1. Diabetes – part I
2. Diabetes – part II
3. Diabetic ketoacidosis
4. Thyroid disorders

Genitourinary

1. Common genitourinary problems
2. Sexually transmitted infection syndromes

Renal

1. Approach to acute kidney injury

Hepatic

1. Approach to Liver Tests

Hematology

1. Anemia

Psychiatry

1. Grief
2. Depression
3. Psychosis
4. Acute Stress Reaction
5. Post-traumatic Stress Disorder

Quality Improvement

1. Core concepts in quality improvement
