# Supplementary material for: Transition to active learning in rural Nepal: an adaptable and scalable curriculum development model
Source: BMC Med Educ. 2019 Feb 20;19:61. doi: 10.1186/s12909-019-1492-3 (PMC6383231; doi:10.1186/s12909-019-1492-3)
Supplement: Supplementary file 2 — Master PowerPoint template, used to generate topic-specific templates. (PPTX 419 kb) [file 12909_2019_1492_MOESM2_ESM.pptx]

## Slide 1
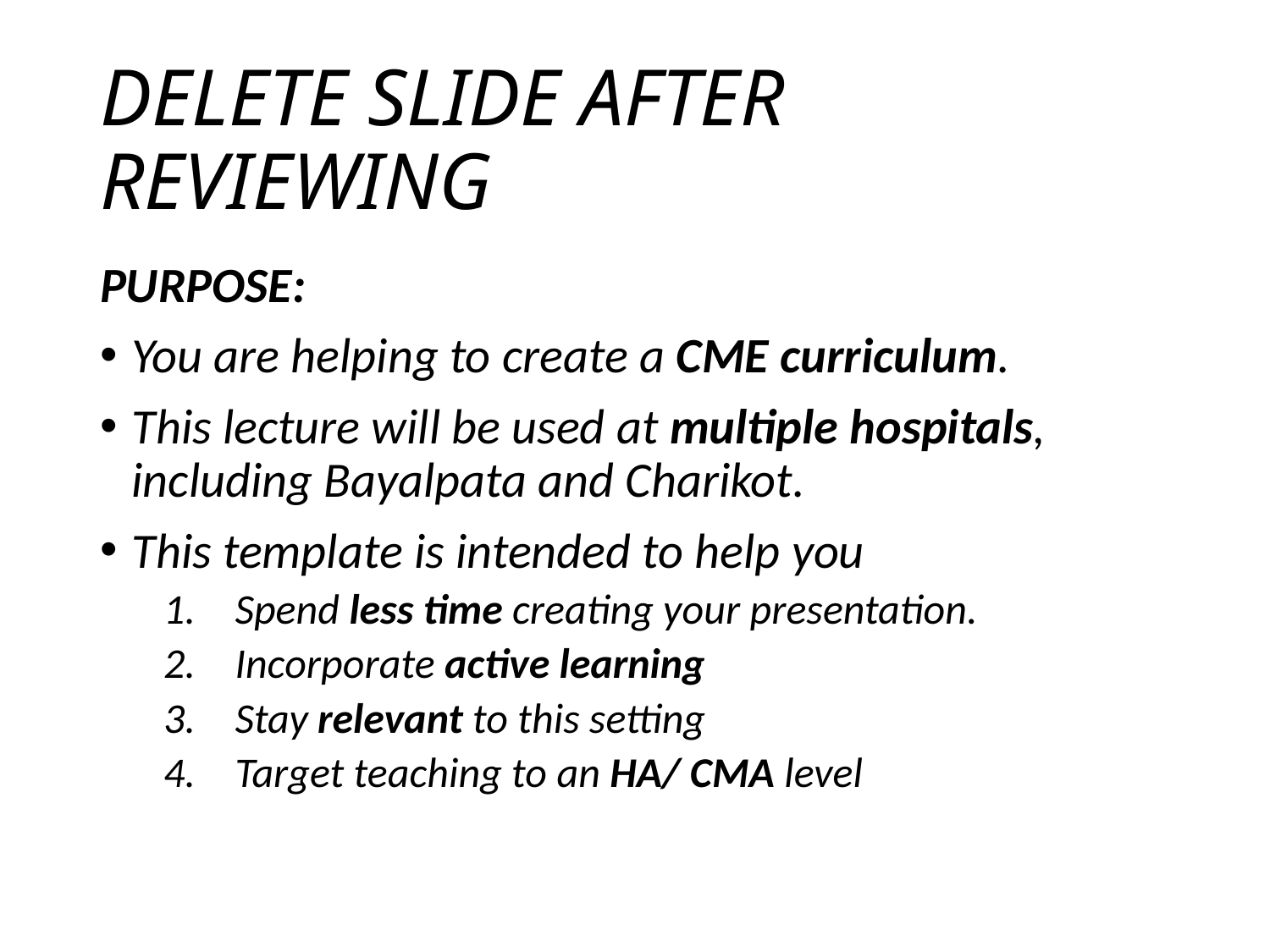

# DELETE SLIDE AFTER REVIEWING
PURPOSE:
You are helping to create a CME curriculum.
This lecture will be used at multiple hospitals, including Bayalpata and Charikot.
This template is intended to help you
Spend less time creating your presentation.
Incorporate active learning
Stay relevant to this setting
Target teaching to an HA/ CMA level

## Slide 2
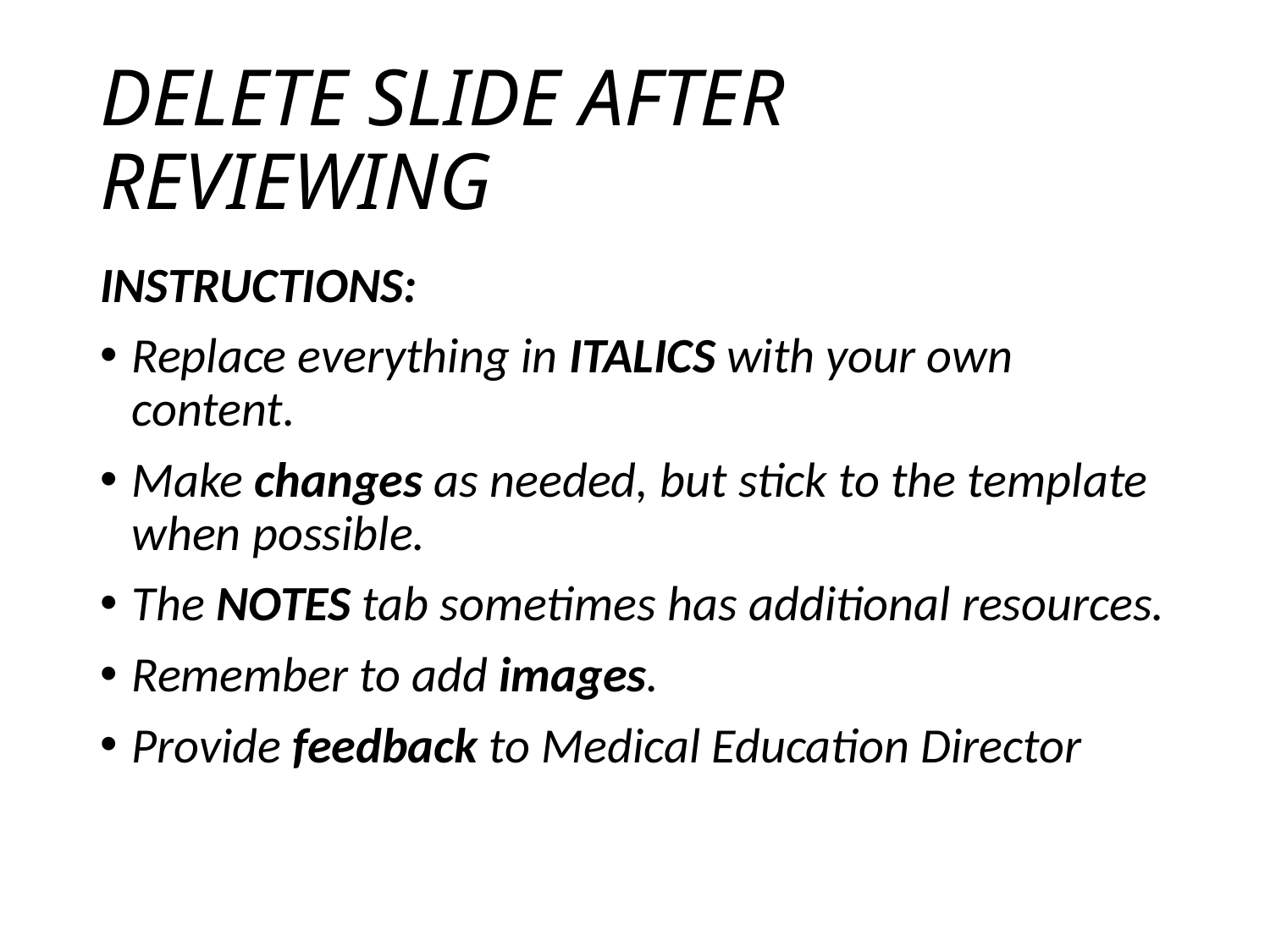

# DELETE SLIDE AFTER REVIEWING
INSTRUCTIONS:
Replace everything in ITALICS with your own content.
Make changes as needed, but stick to the template when possible.
The NOTES tab sometimes has additional resources.
Remember to add images.
Provide feedback to Medical Education Director

## Slide 3
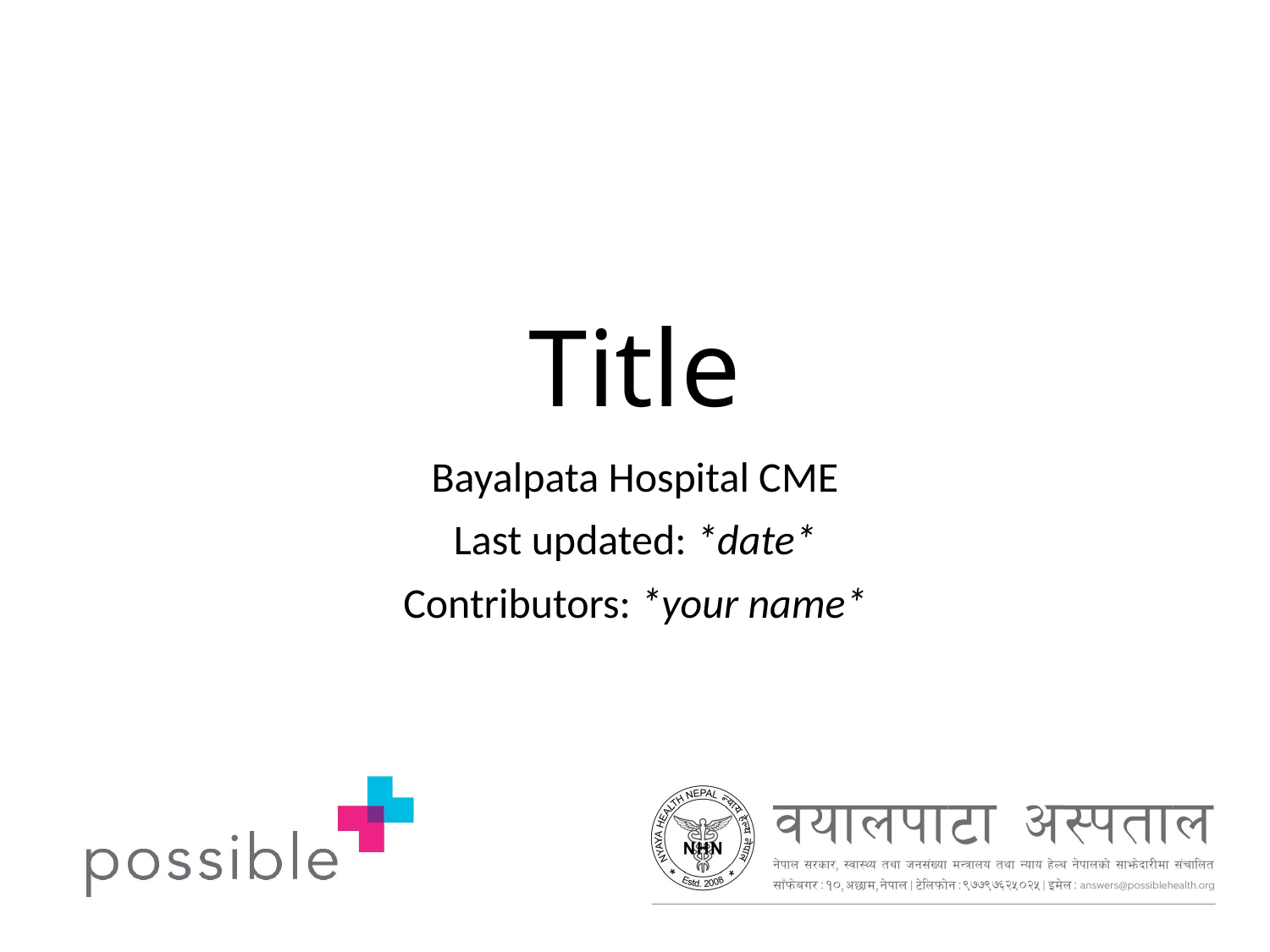

# Title
Bayalpata Hospital CME
Last updated: *date*
Contributors: *your name*

## Slide 4
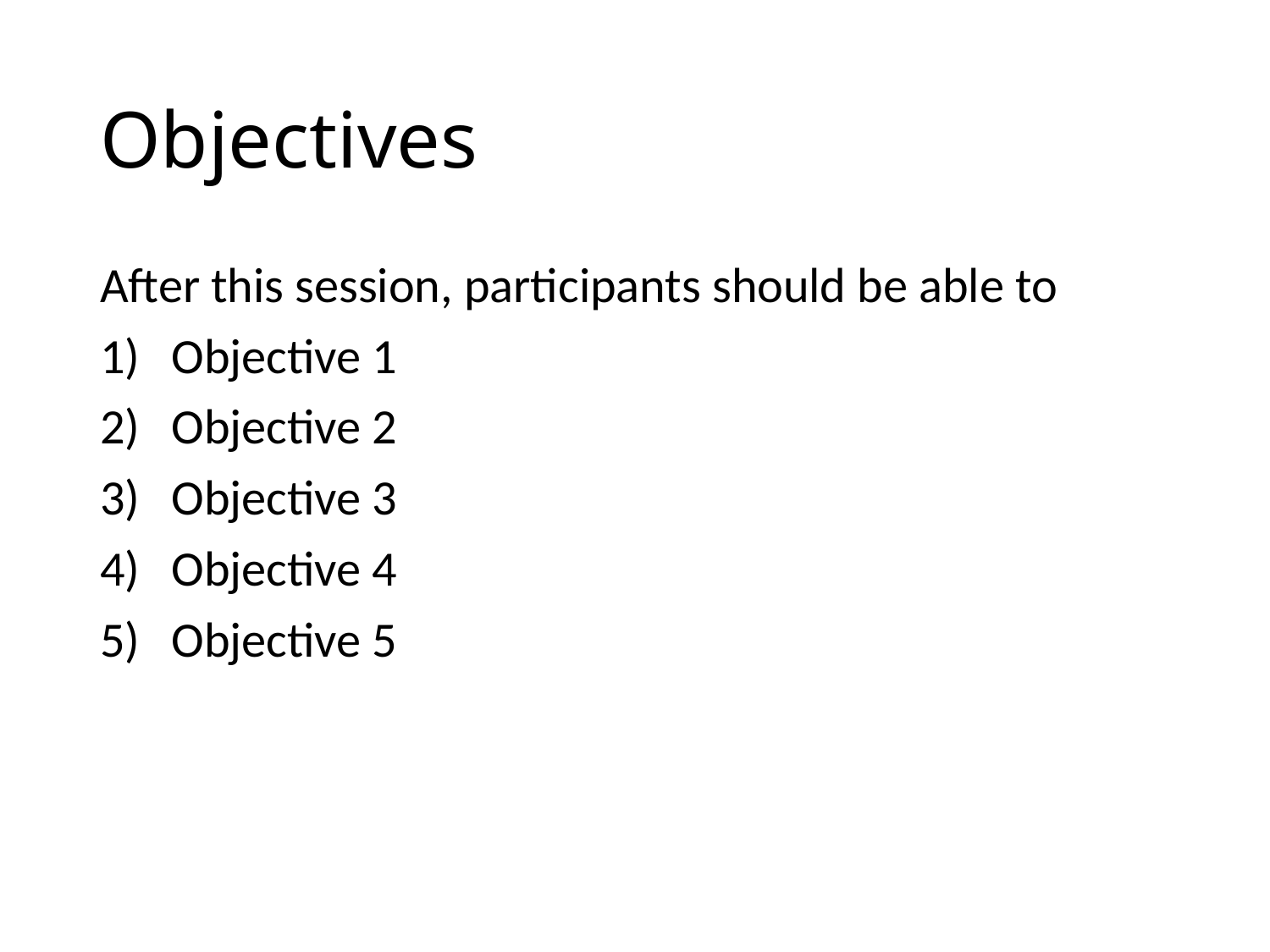

# Objectives
After this session, participants should be able to
Objective 1
Objective 2
Objective 3
Objective 4
Objective 5

## Slide 5
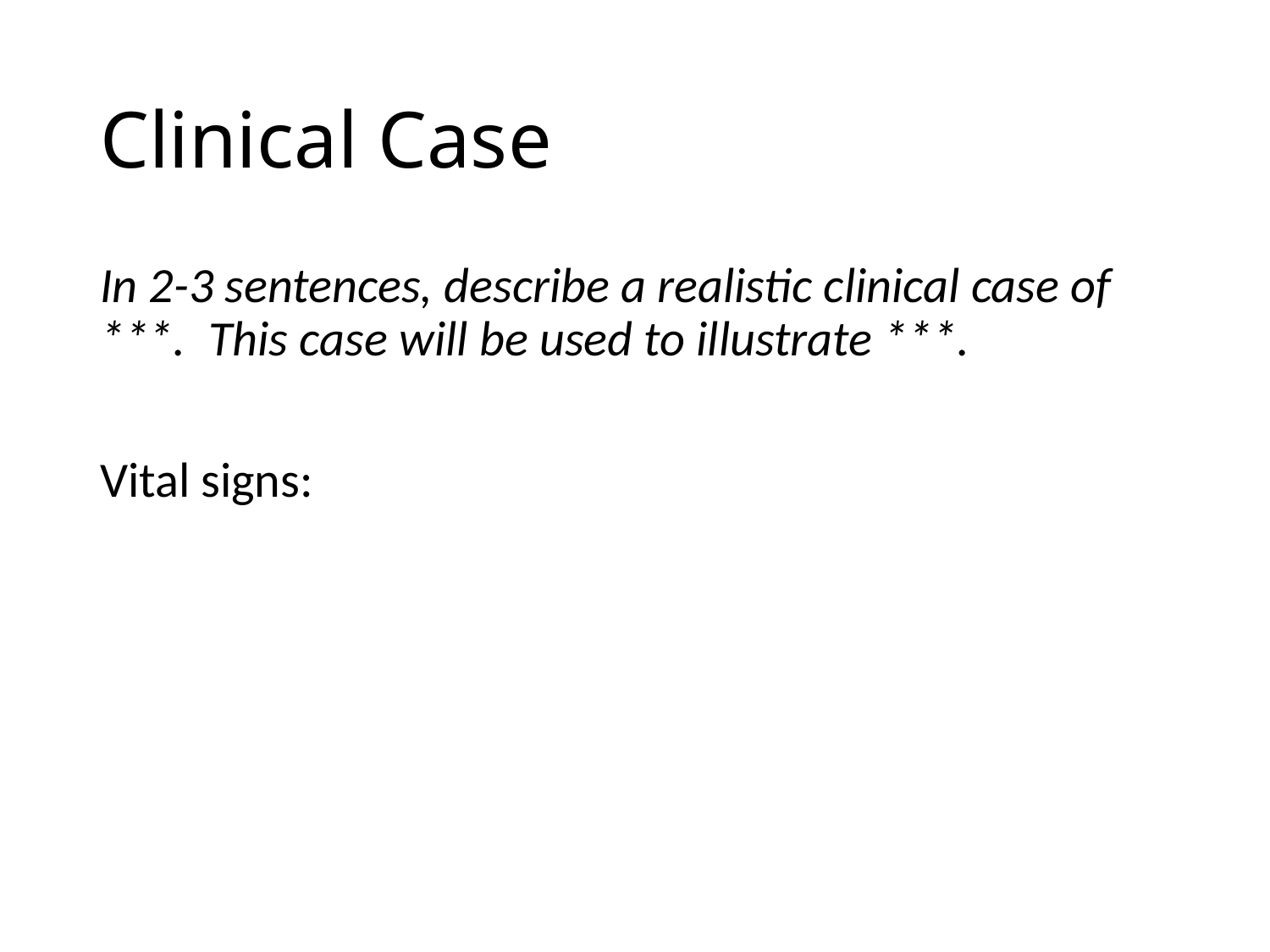

# Clinical Case
In 2-3 sentences, describe a realistic clinical case of ***. This case will be used to illustrate ***.
Vital signs:

## Slide 6
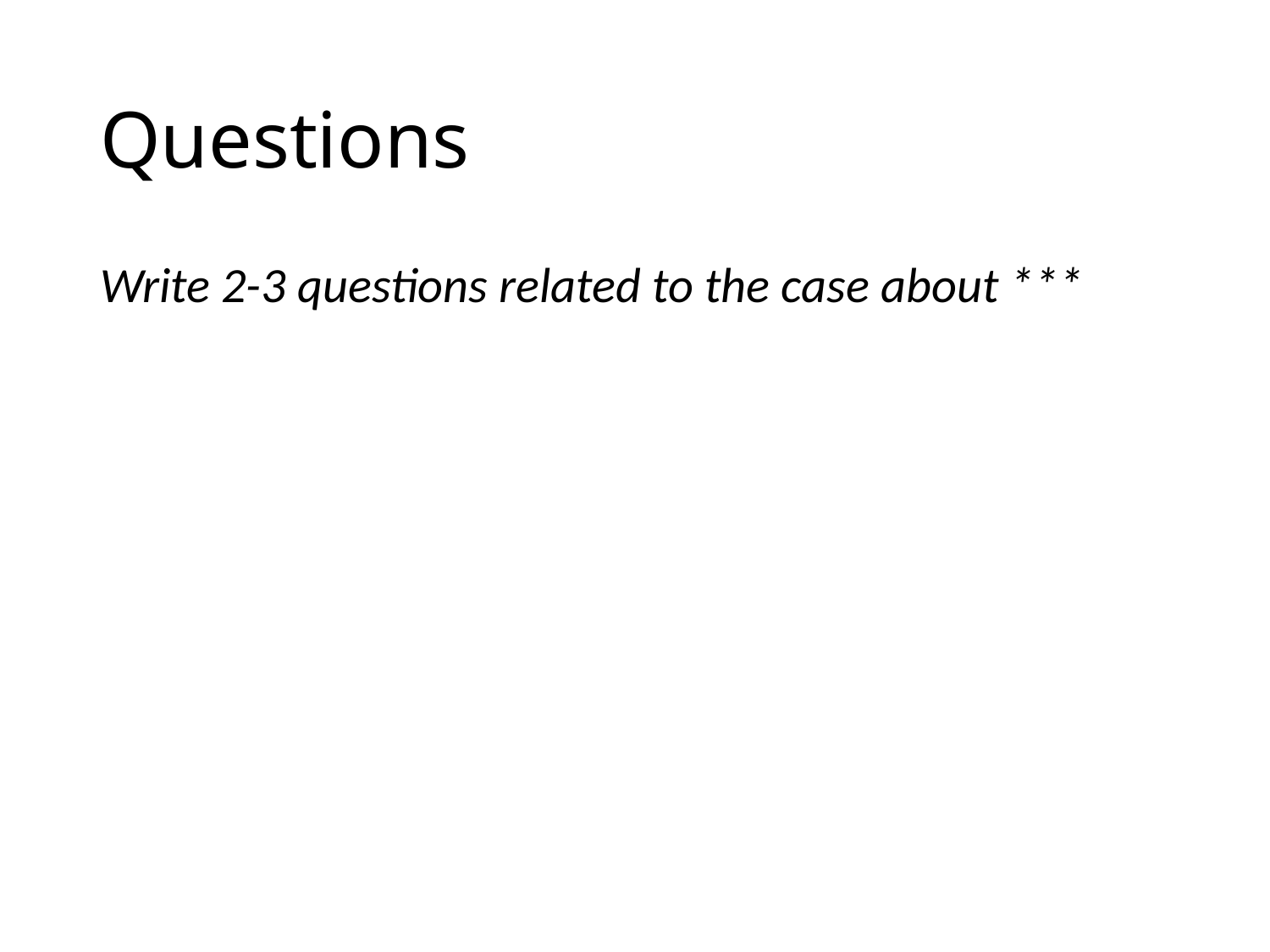

# Questions
Write 2-3 questions related to the case about ***

## Slide 7
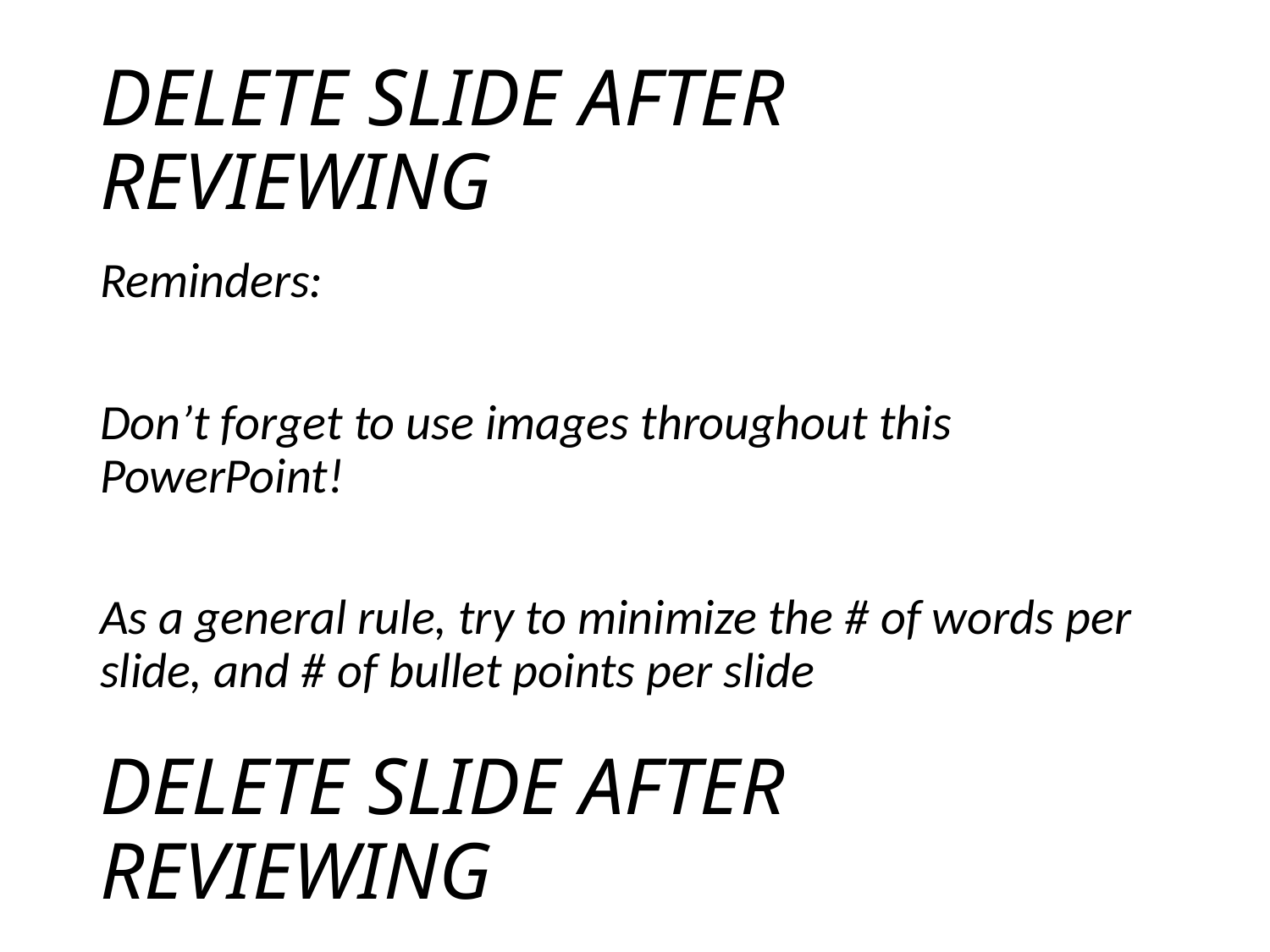

# DELETE SLIDE AFTER REVIEWING
Reminders:
Don’t forget to use images throughout this PowerPoint!
As a general rule, try to minimize the # of words per slide, and # of bullet points per slide
DELETE SLIDE AFTER REVIEWING

## Slide 8
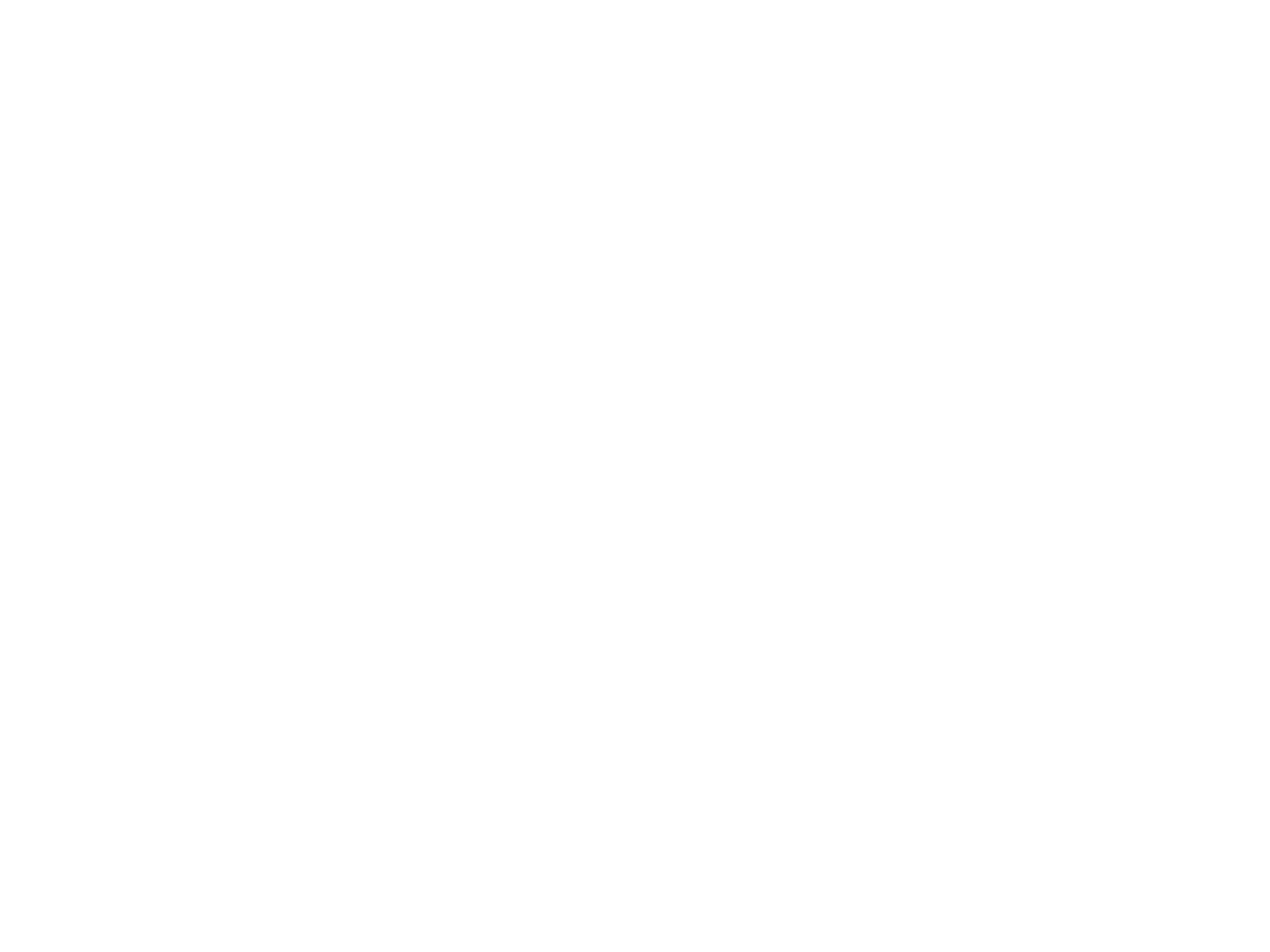

#

## Slide 9
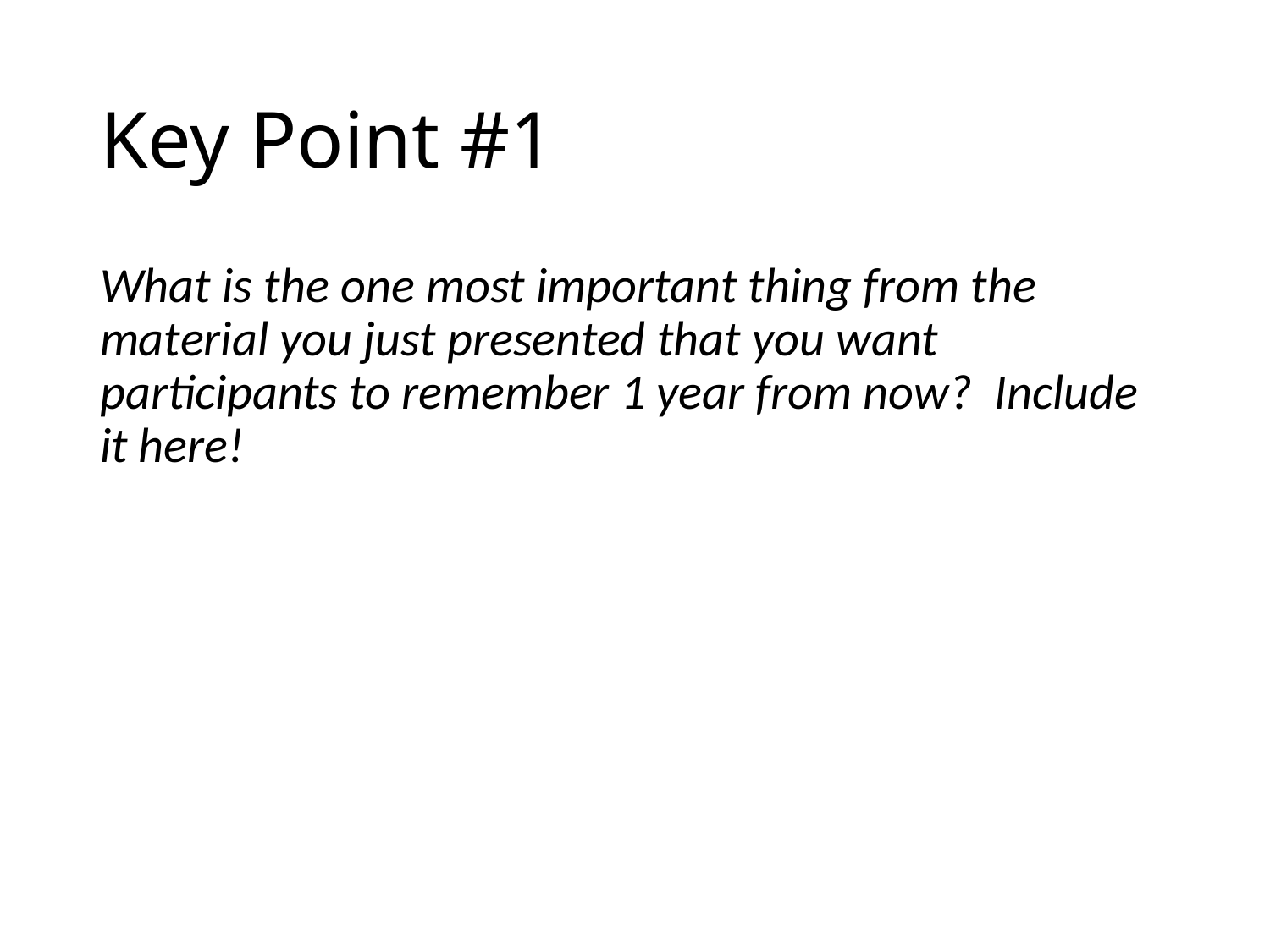

# Key Point #1
What is the one most important thing from the material you just presented that you want participants to remember 1 year from now? Include it here!

## Slide 10
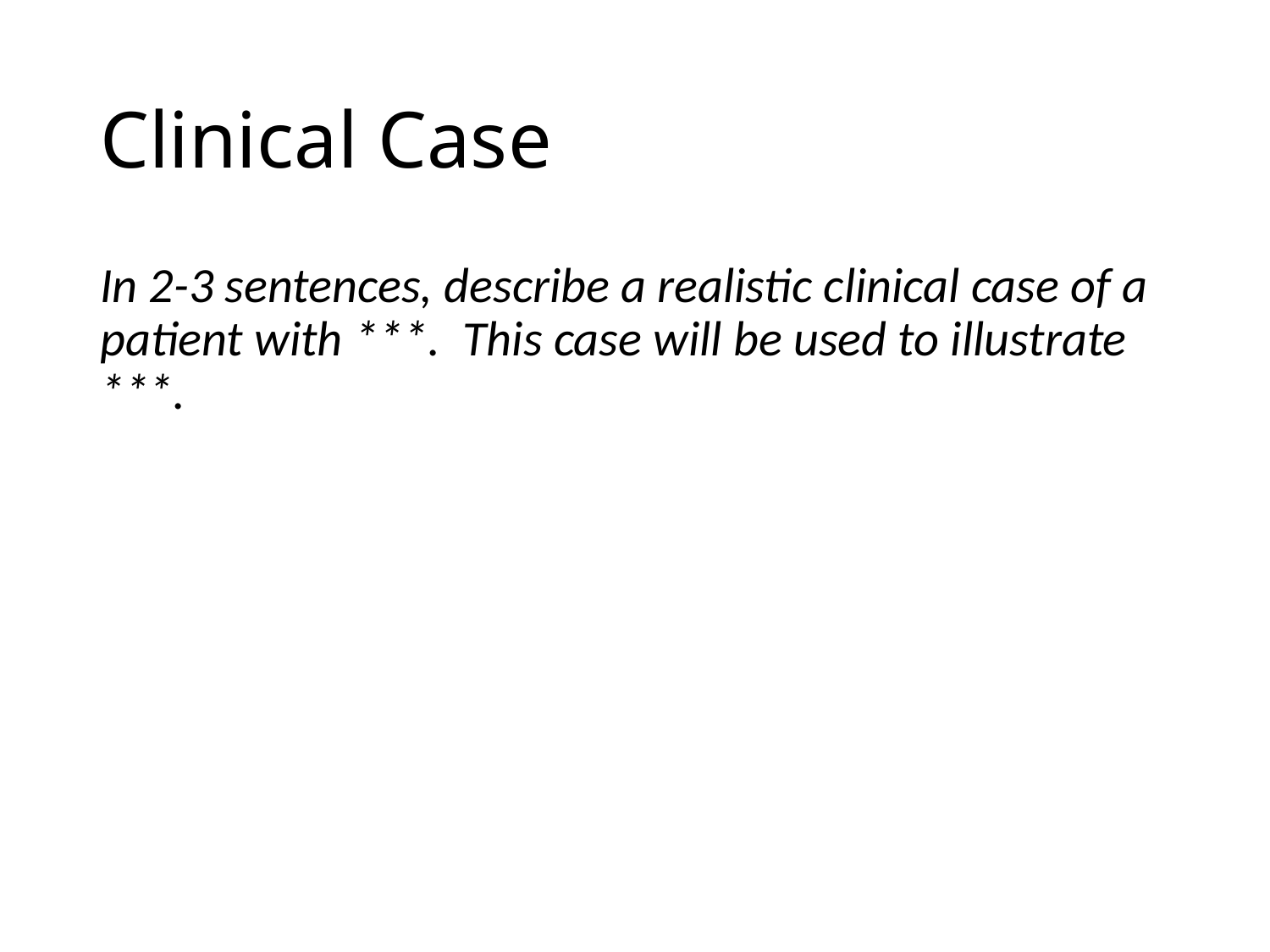

# Clinical Case
In 2-3 sentences, describe a realistic clinical case of a patient with ***. This case will be used to illustrate ***.

## Slide 11
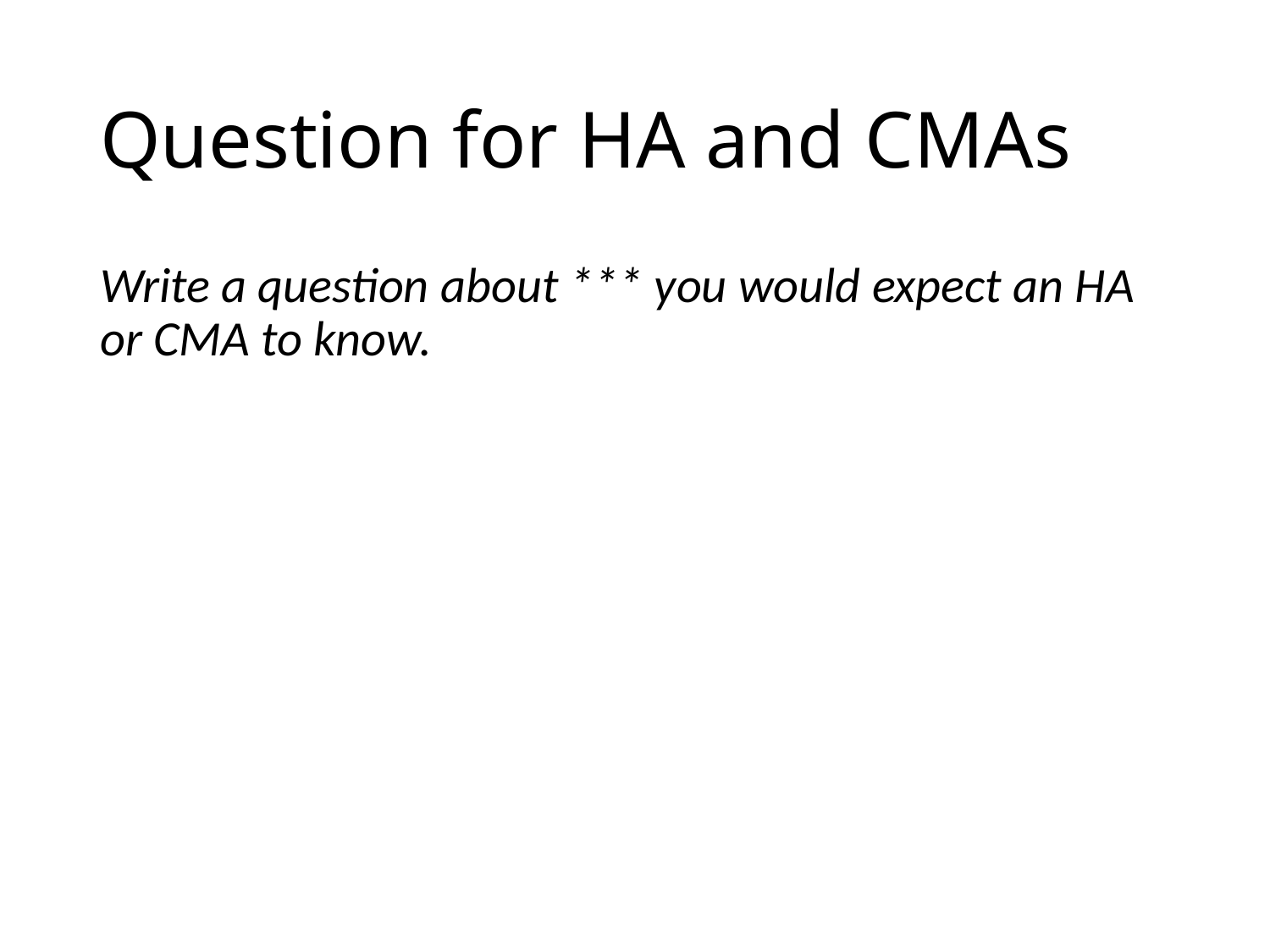

# Question for HA and CMAs
Write a question about *** you would expect an HA or CMA to know.

## Slide 12
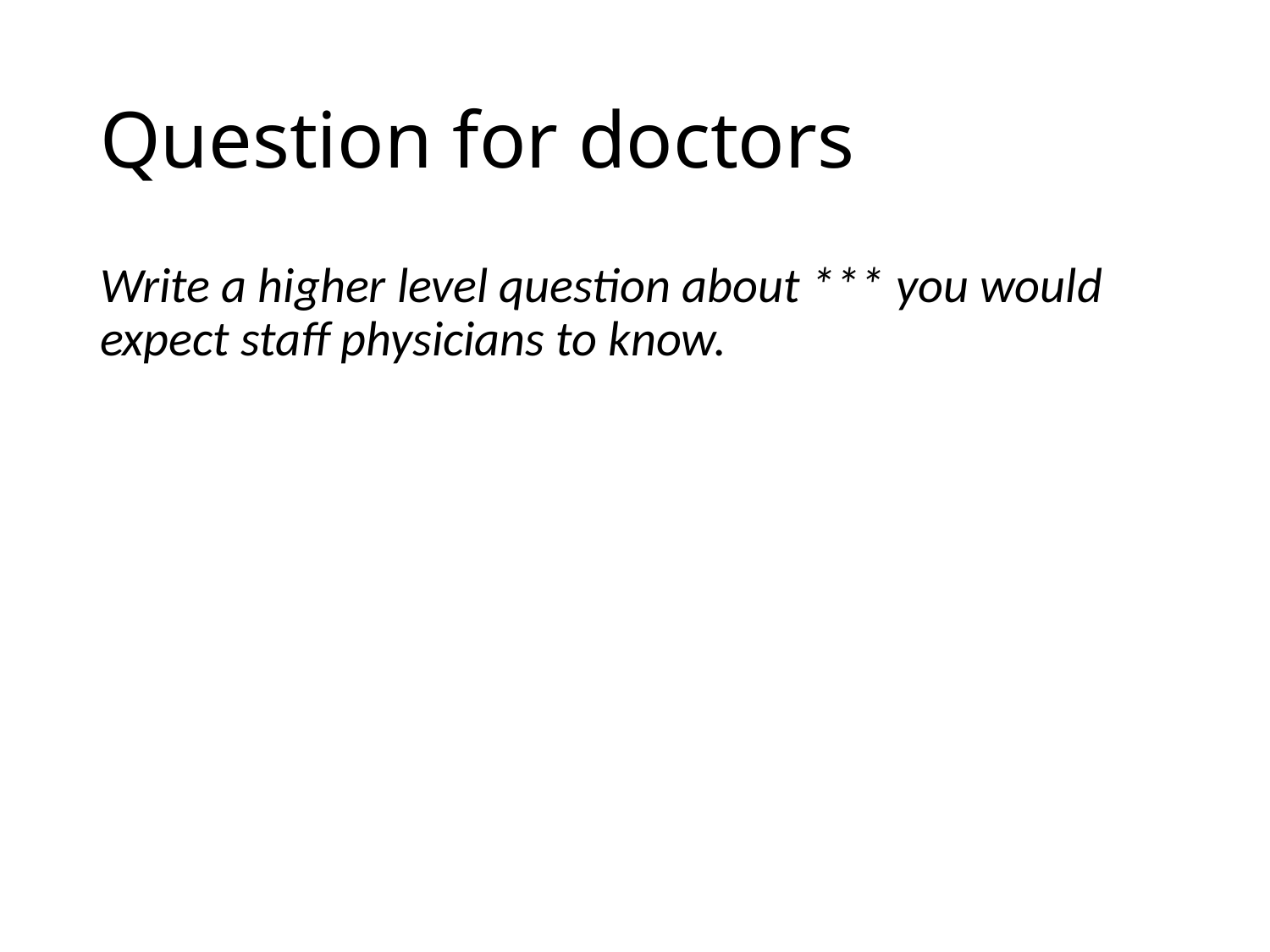

# Question for doctors
Write a higher level question about *** you would expect staff physicians to know.

## Slide 13
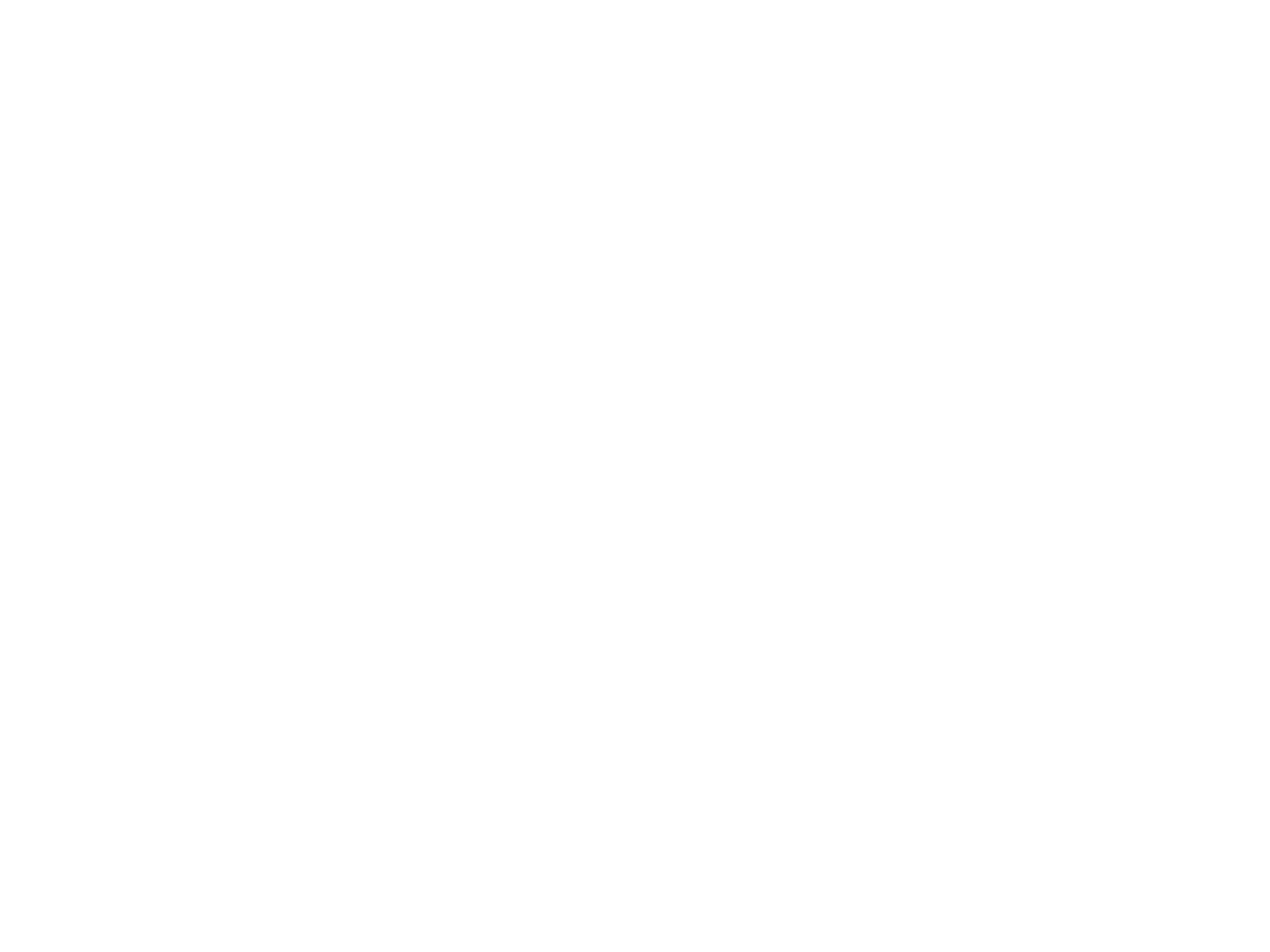

#

## Slide 14
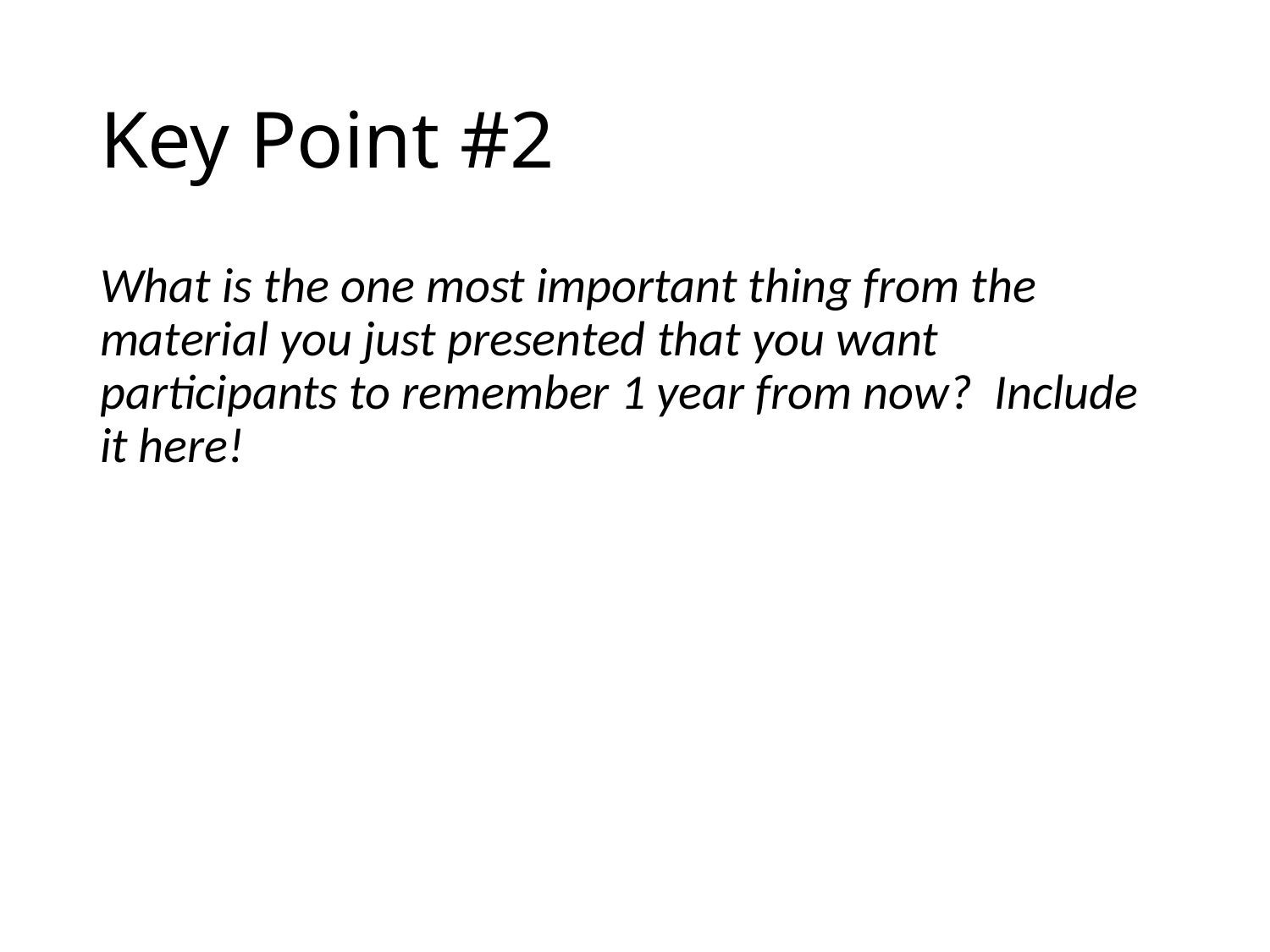

# Key Point #2
What is the one most important thing from the material you just presented that you want participants to remember 1 year from now? Include it here!

## Slide 15
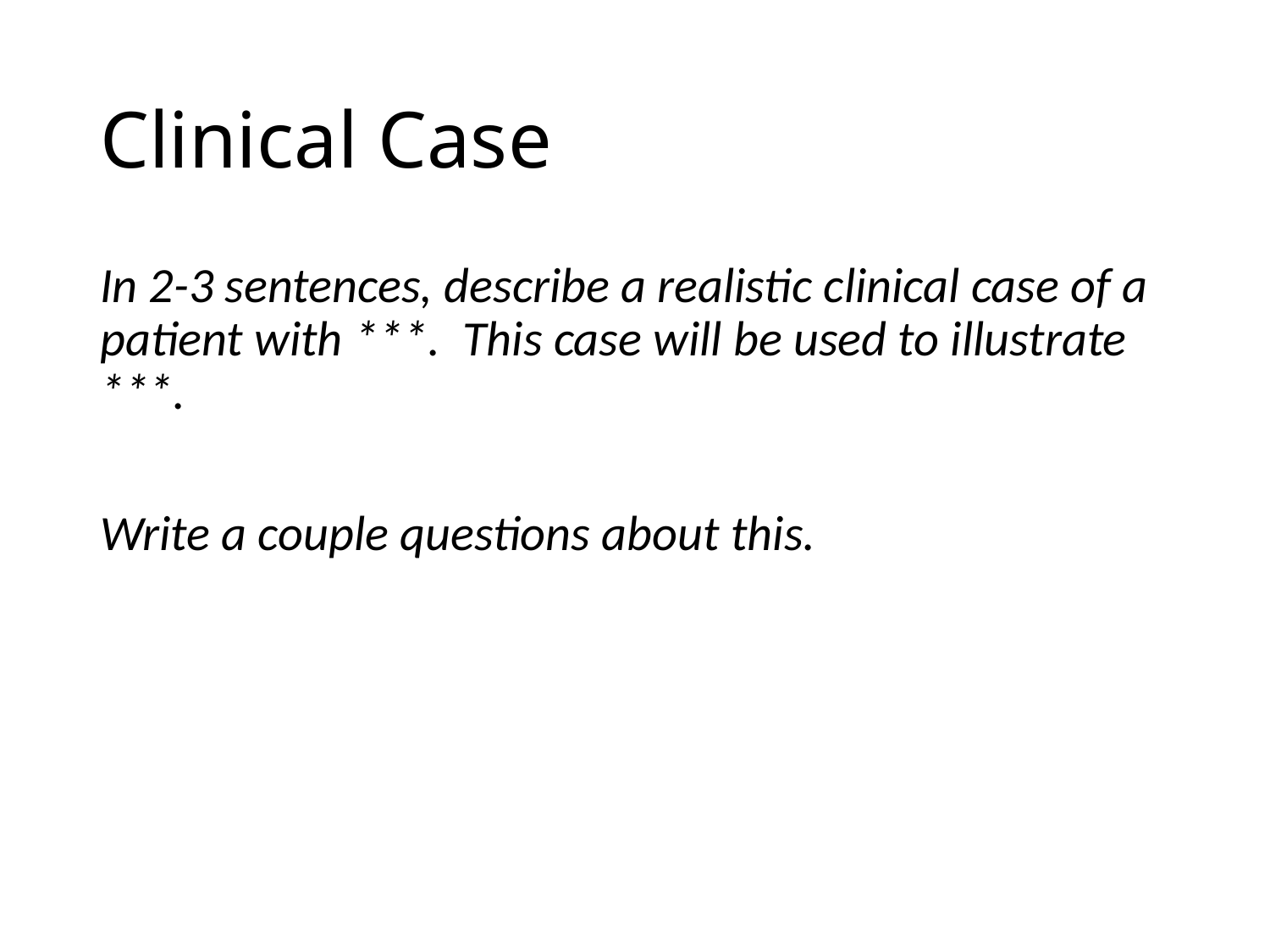

# Clinical Case
In 2-3 sentences, describe a realistic clinical case of a patient with ***. This case will be used to illustrate ***.
Write a couple questions about this.

## Slide 16
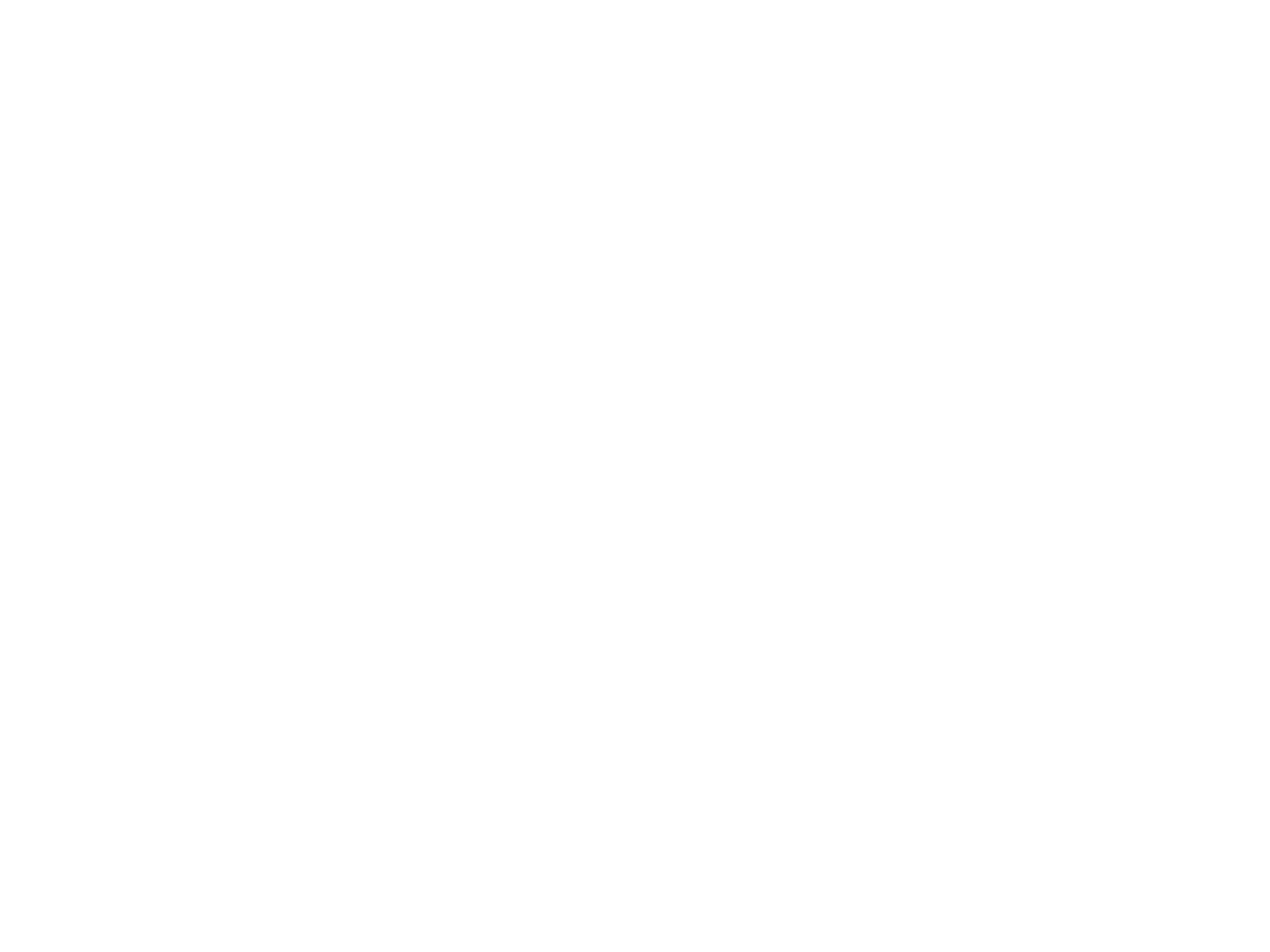

#

## Slide 17
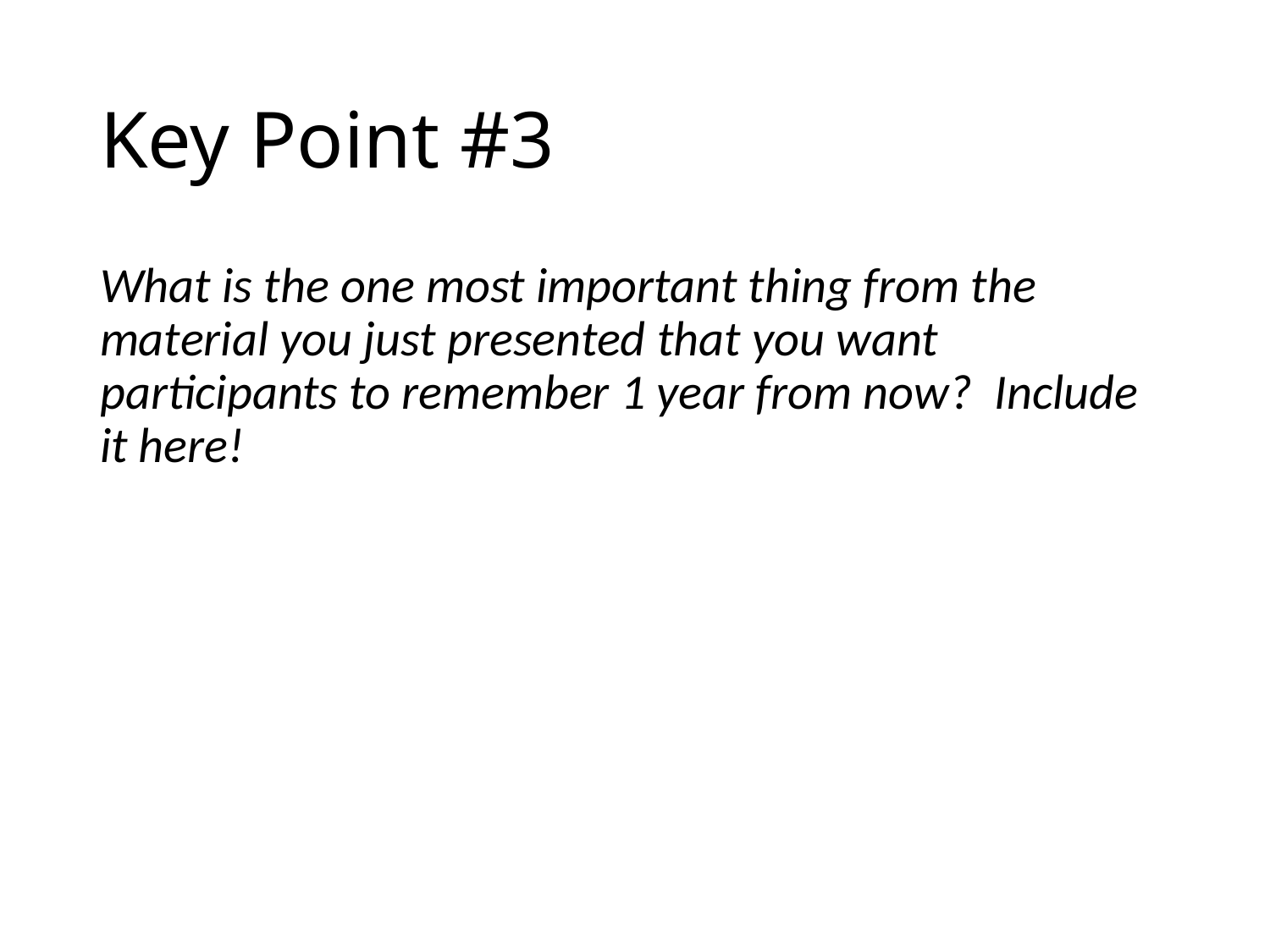

# Key Point #3
What is the one most important thing from the material you just presented that you want participants to remember 1 year from now? Include it here!

## Slide 18
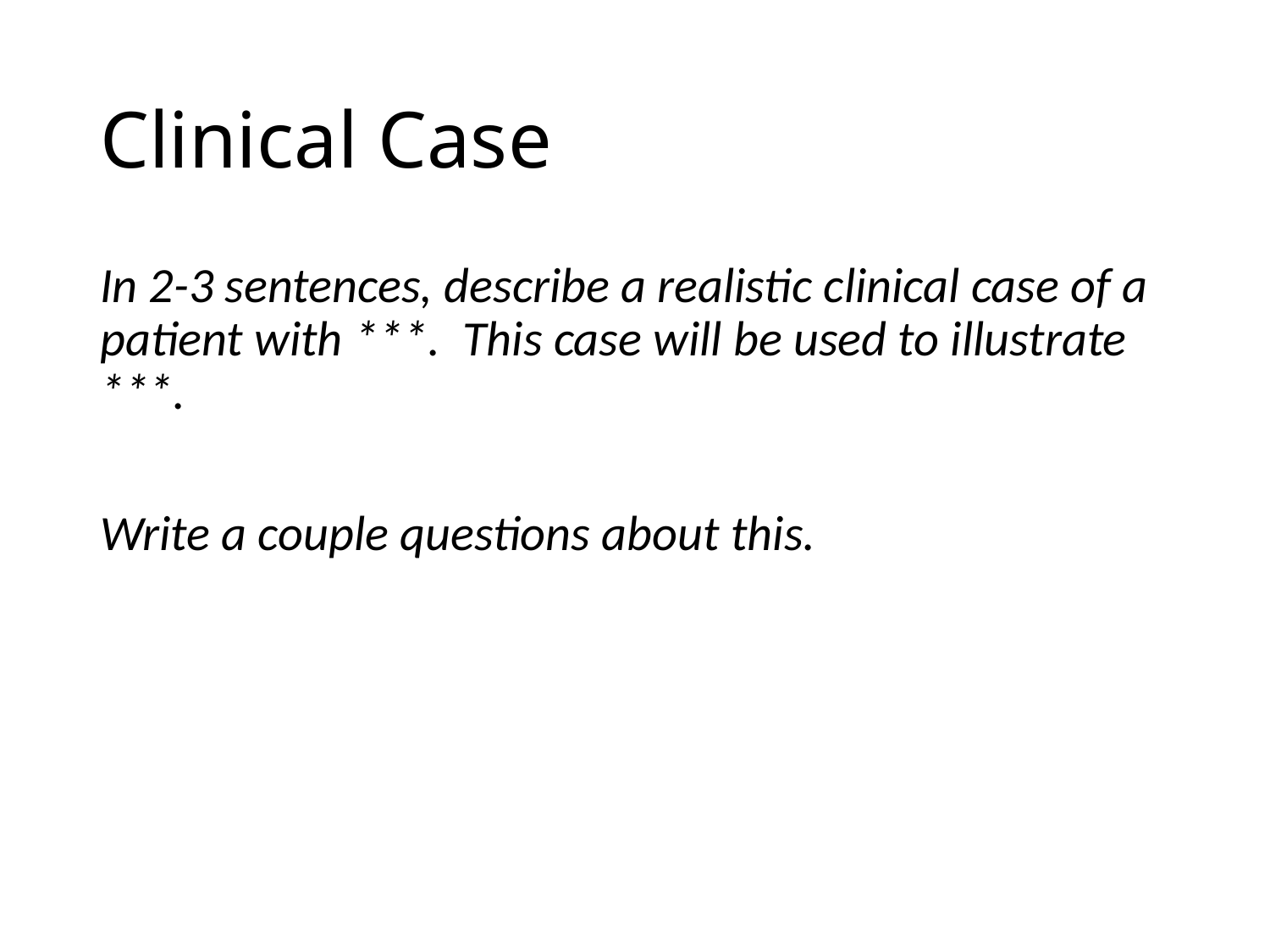

# Clinical Case
In 2-3 sentences, describe a realistic clinical case of a patient with ***. This case will be used to illustrate ***.
Write a couple questions about this.

## Slide 19
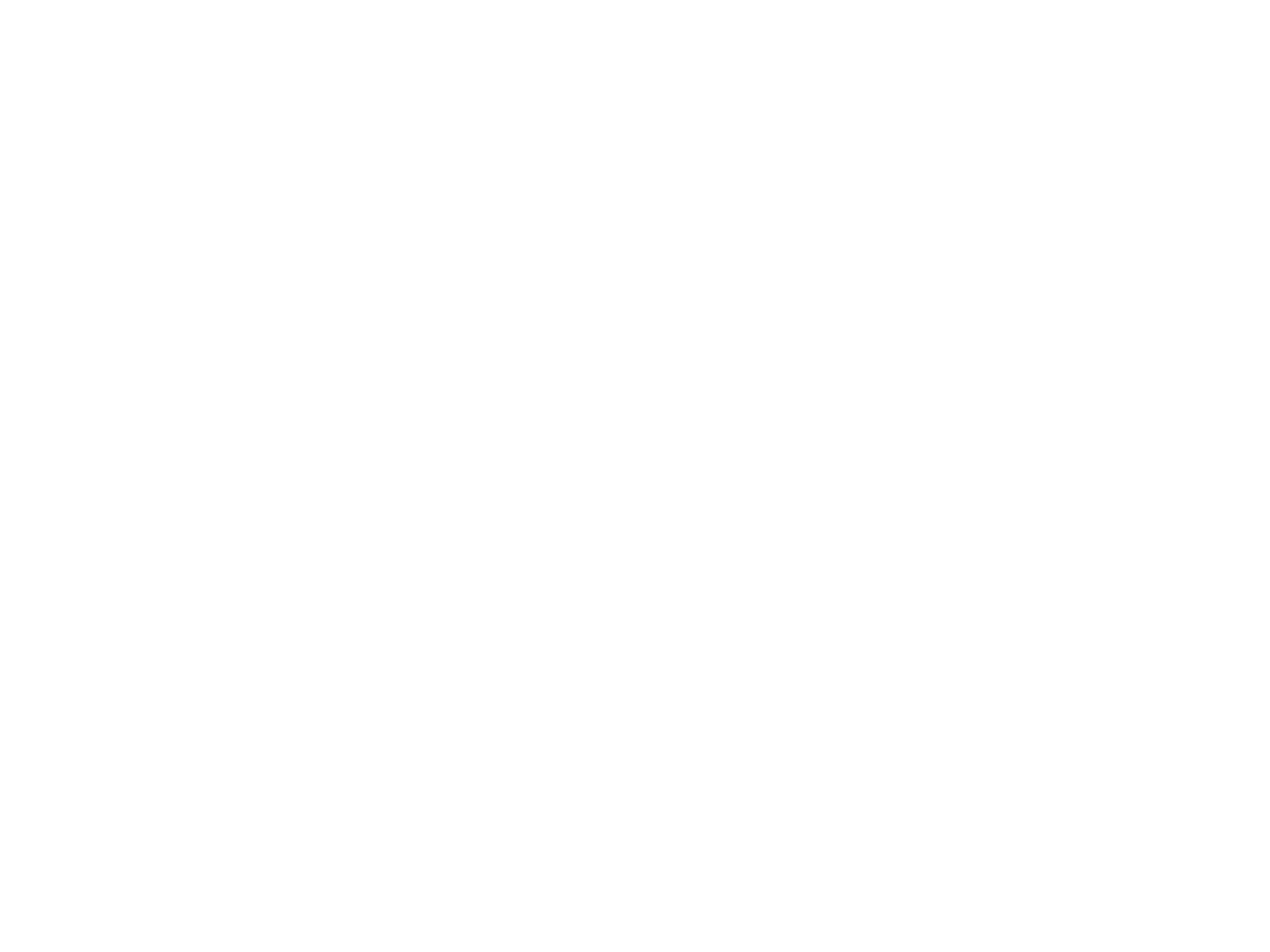

#

## Slide 20
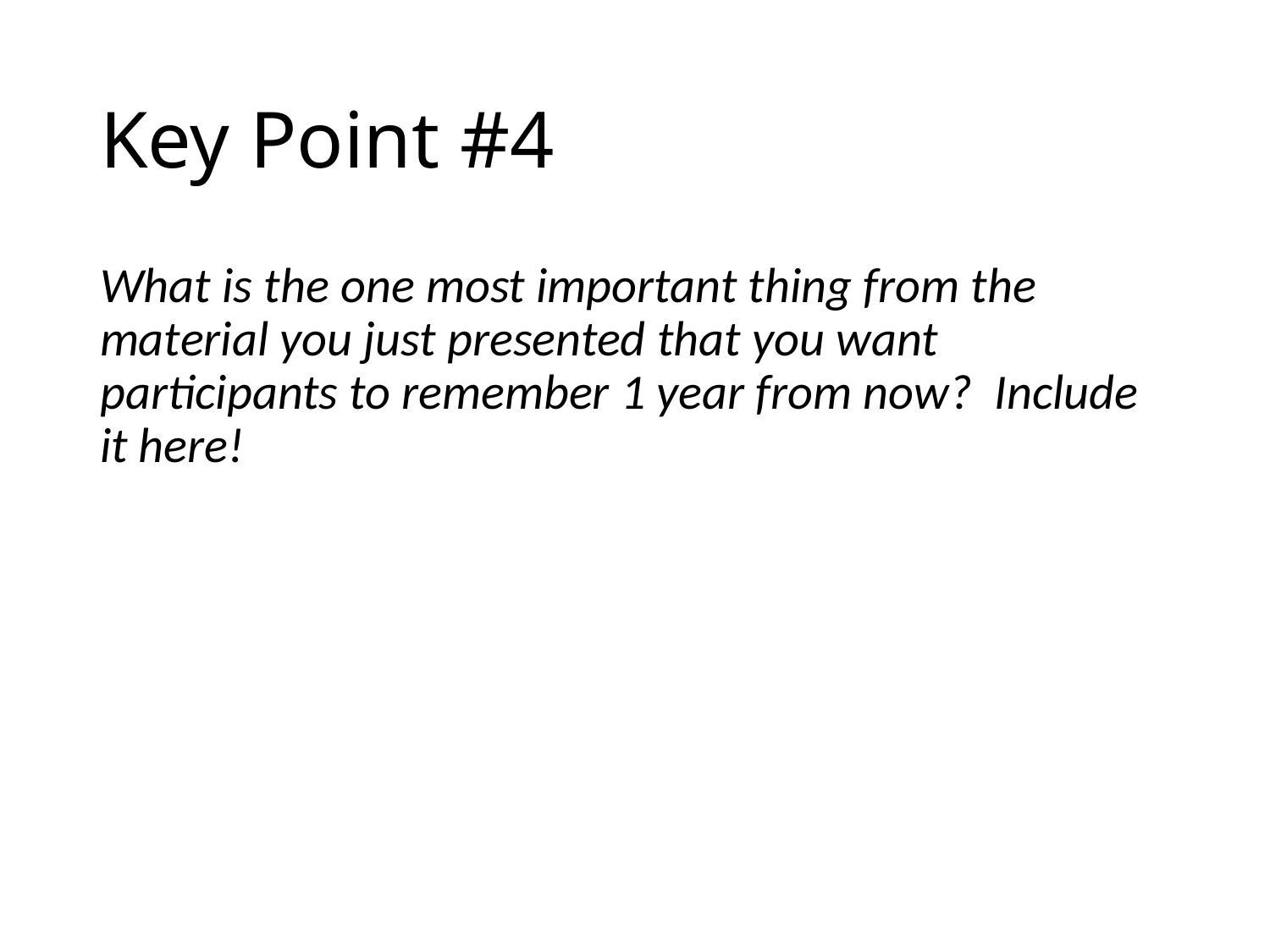

# Key Point #4
What is the one most important thing from the material you just presented that you want participants to remember 1 year from now? Include it here!

## Slide 21
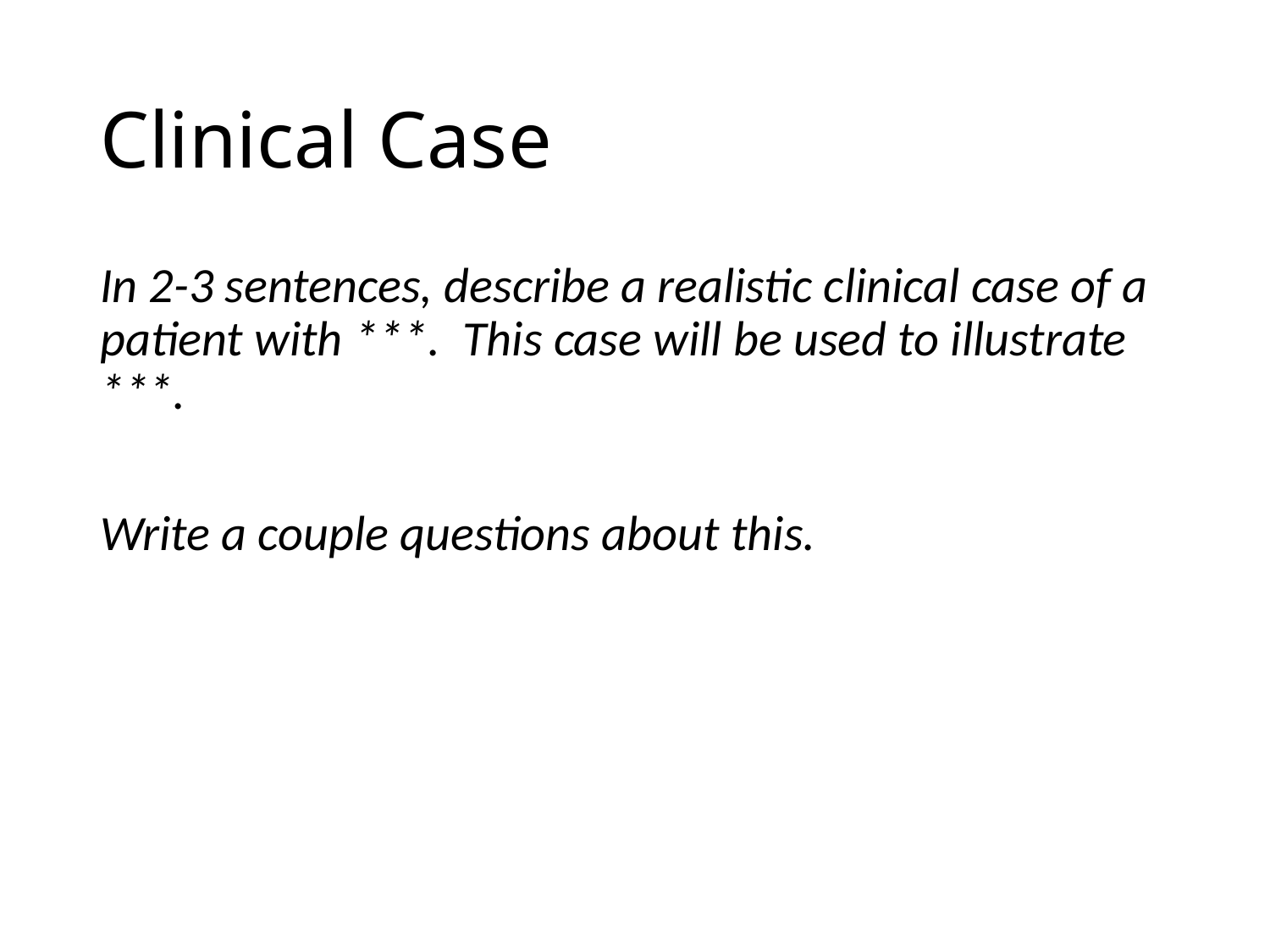

# Clinical Case
In 2-3 sentences, describe a realistic clinical case of a patient with ***. This case will be used to illustrate ***.
Write a couple questions about this.

## Slide 22
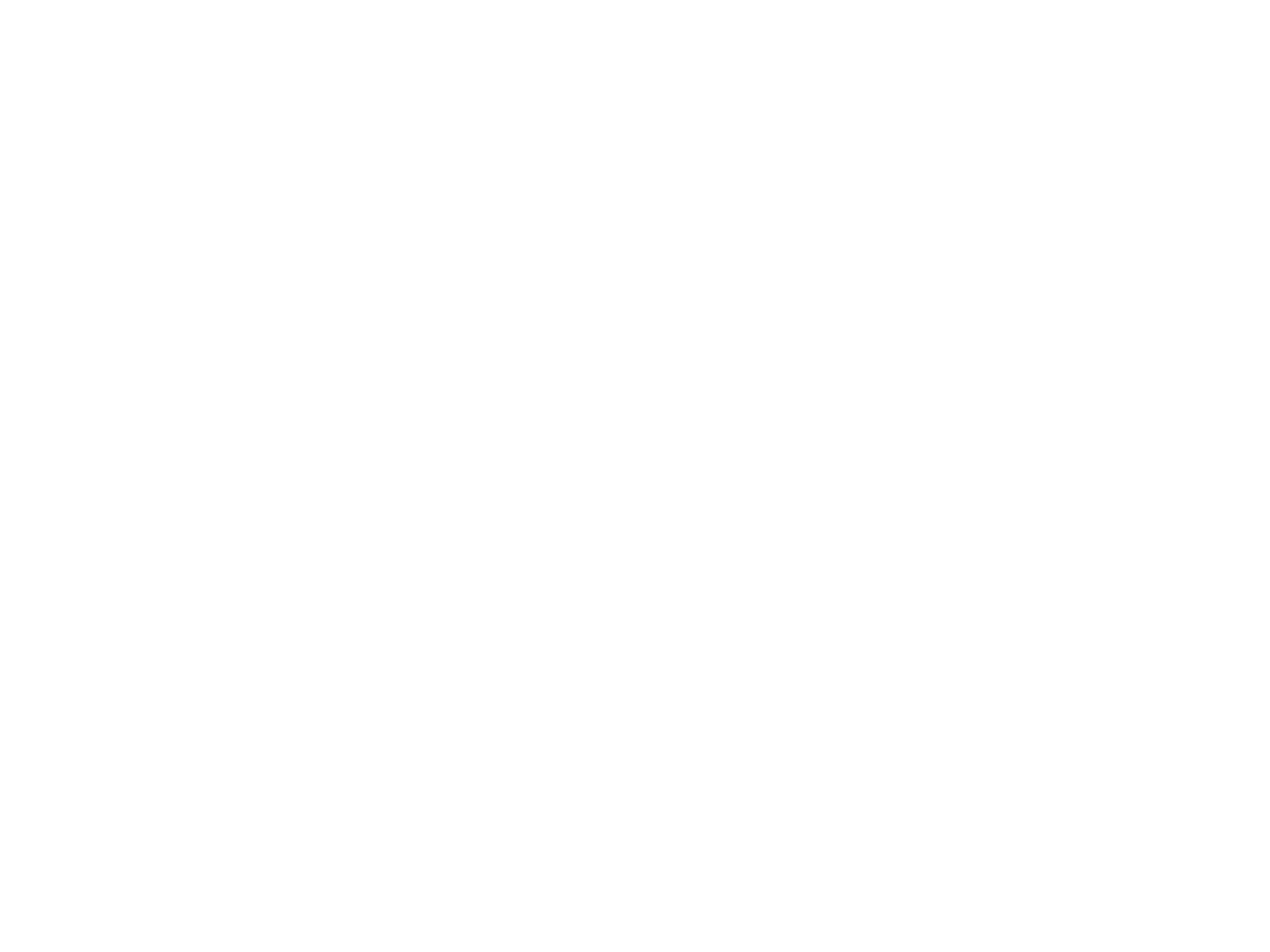

#

## Slide 23
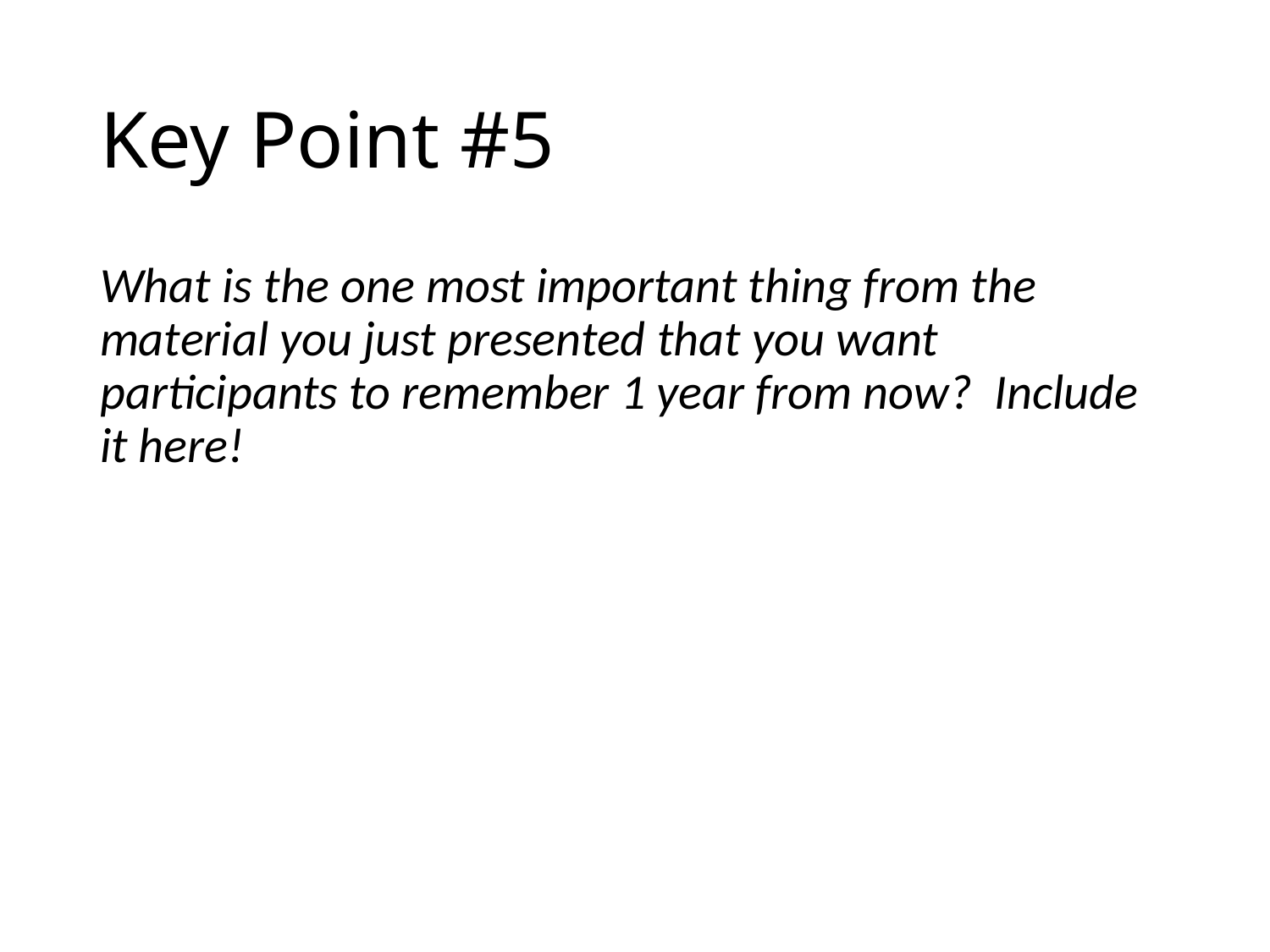

# Key Point #5
What is the one most important thing from the material you just presented that you want participants to remember 1 year from now? Include it here!

## Slide 24
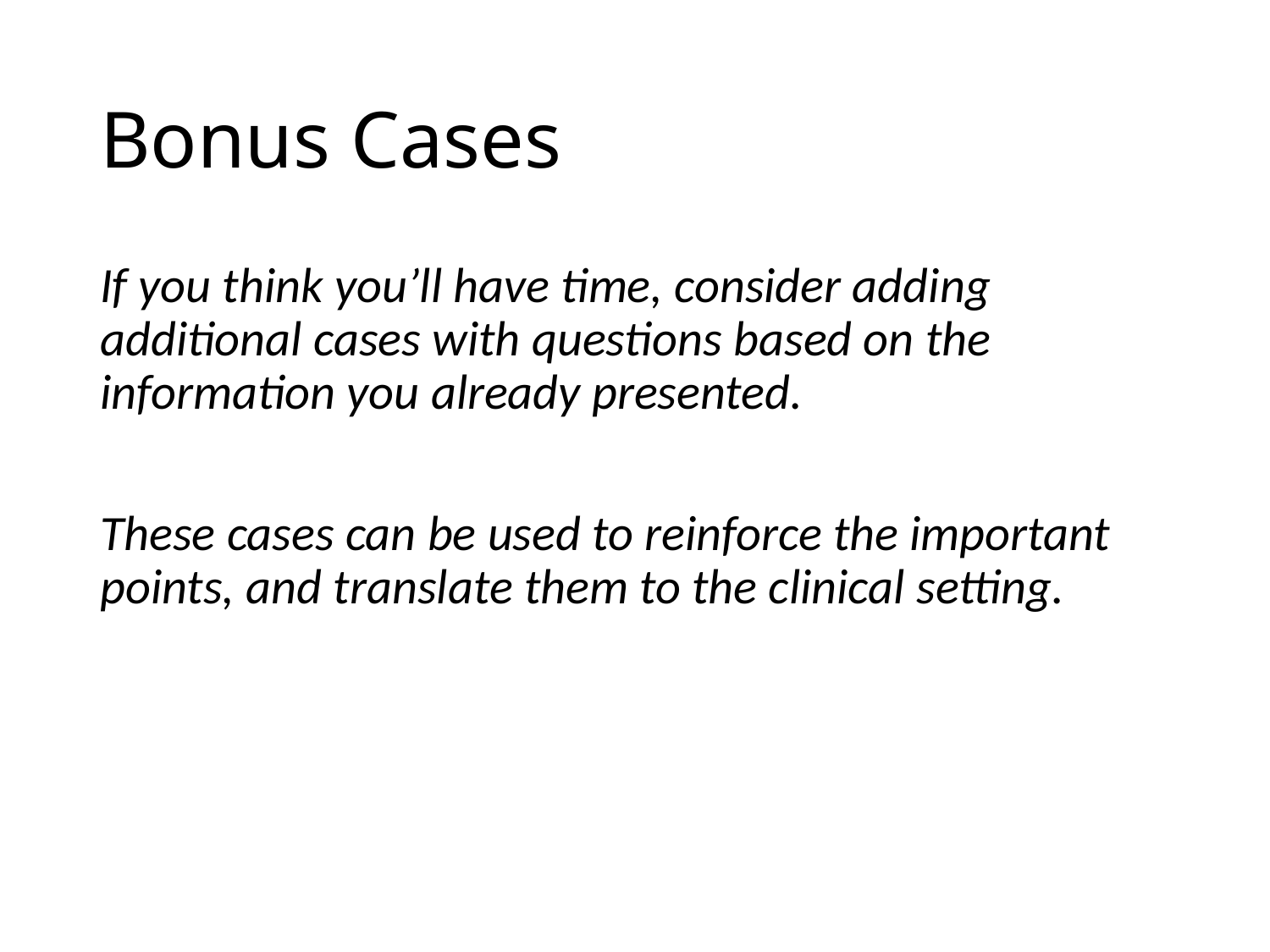

# Bonus Cases
If you think you’ll have time, consider adding additional cases with questions based on the information you already presented.
These cases can be used to reinforce the important points, and translate them to the clinical setting.

## Slide 25
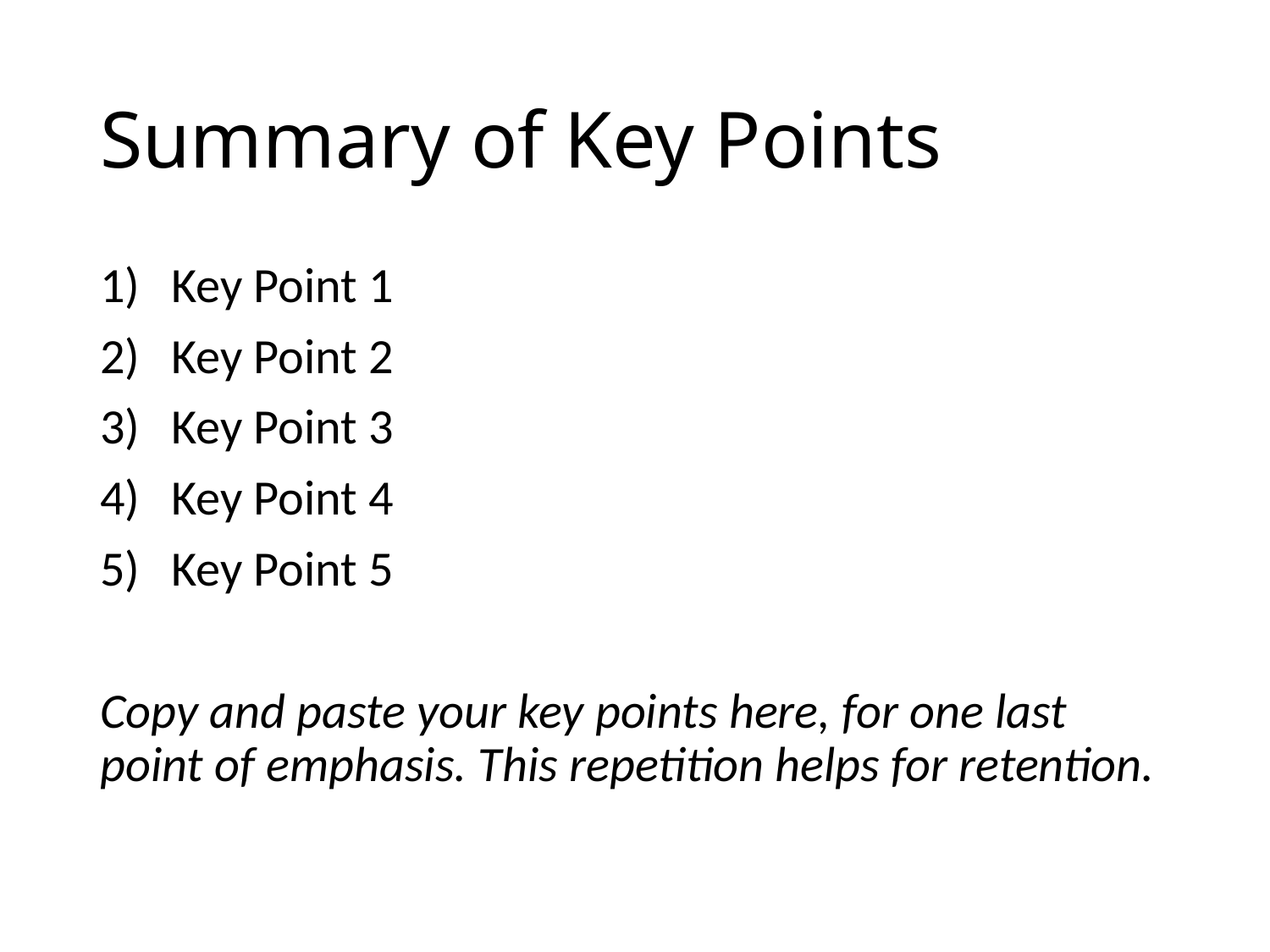

# Summary of Key Points
Key Point 1
Key Point 2
Key Point 3
Key Point 4
Key Point 5
Copy and paste your key points here, for one last point of emphasis. This repetition helps for retention.

## Slide 26
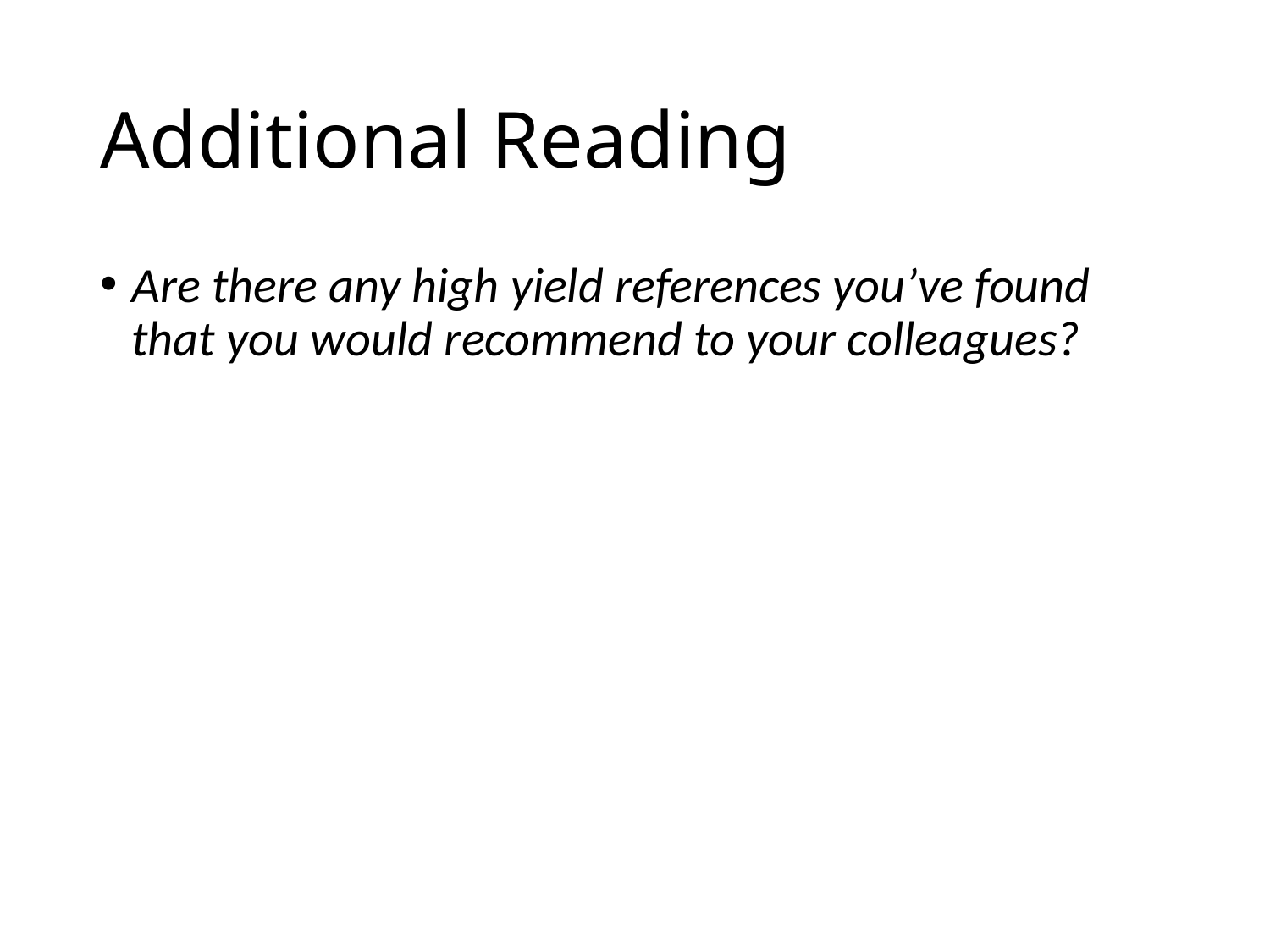

# Additional Reading
Are there any high yield references you’ve found that you would recommend to your colleagues?

## Slide 27
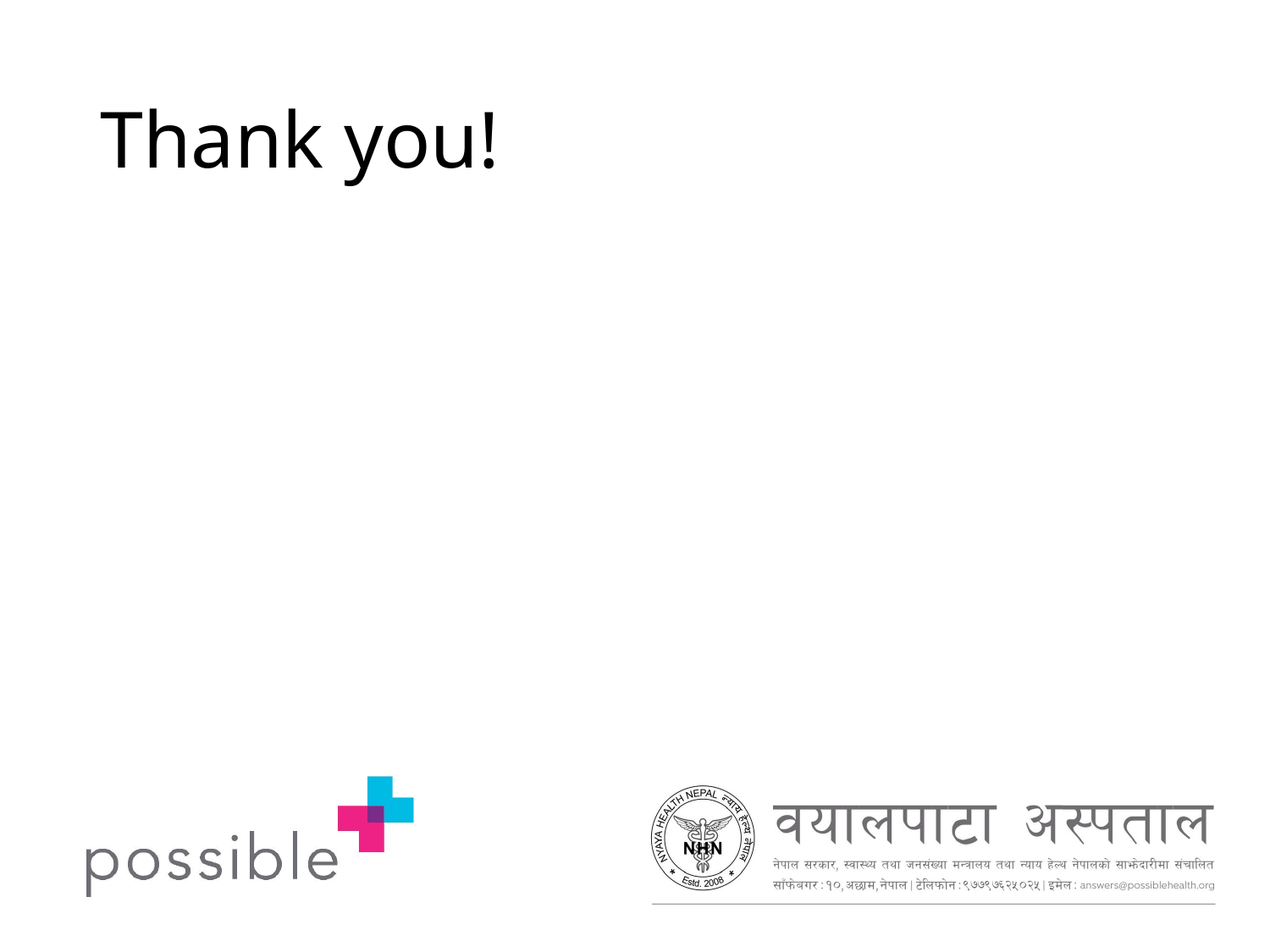

# Thank you!

## Slide 28
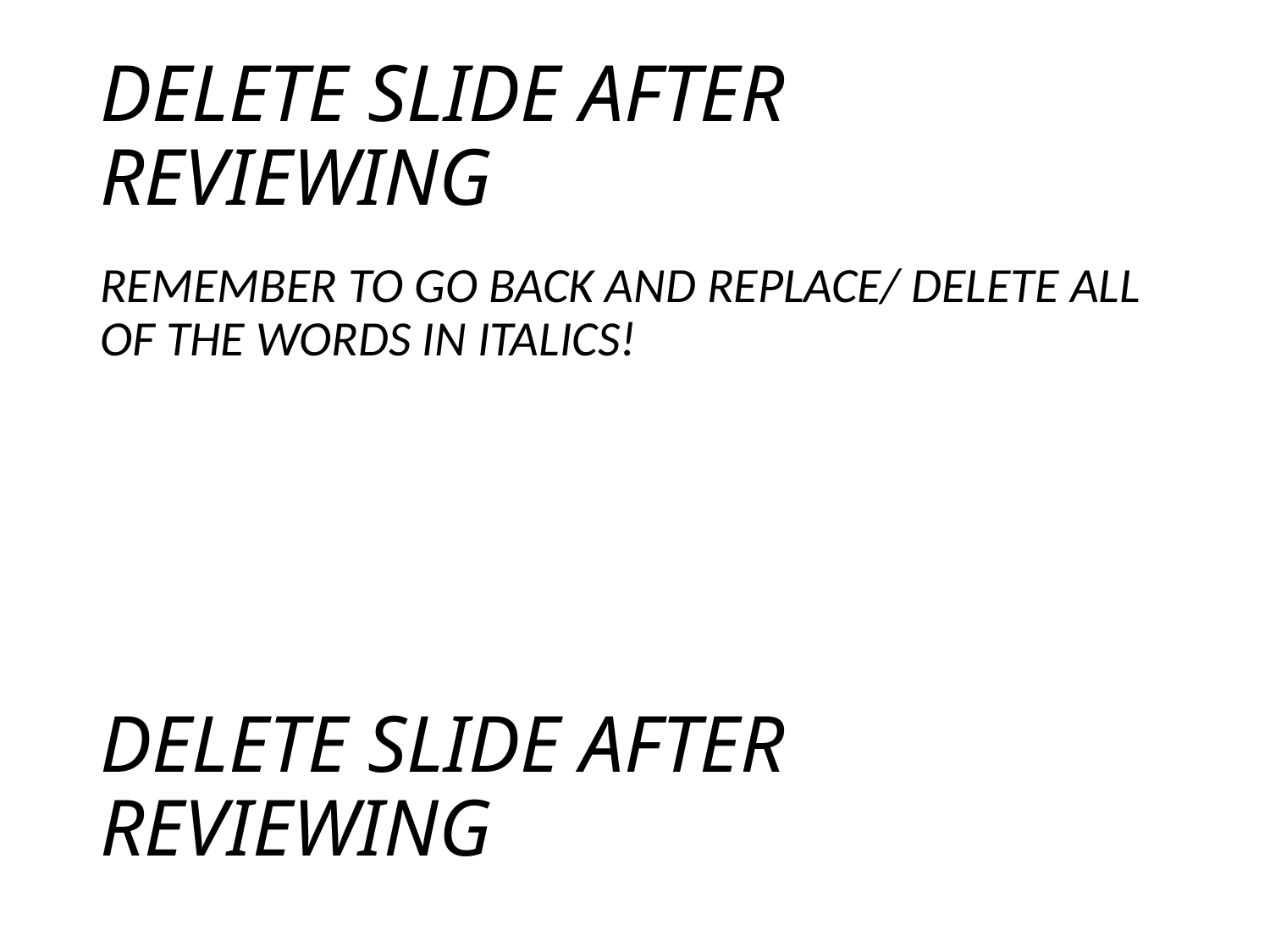

# DELETE SLIDE AFTER REVIEWING
REMEMBER TO GO BACK AND REPLACE/ DELETE ALL OF THE WORDS IN ITALICS!
DELETE SLIDE AFTER REVIEWING
