# Supplementary material for: Transition to active learning in rural Nepal: an adaptable and scalable curriculum development model
Source: BMC Med Educ. 2019 Feb 20;19:61. doi: 10.1186/s12909-019-1492-3 (PMC6383231; doi:10.1186/s12909-019-1492-3)
Supplement: Supplementary file 3 — Sample topic-specific PowerPoint template: “Approach to Heart Failure”. (PPTX 548 kb) [file 12909_2019_1492_MOESM3_ESM.pptx]

## Slide 1
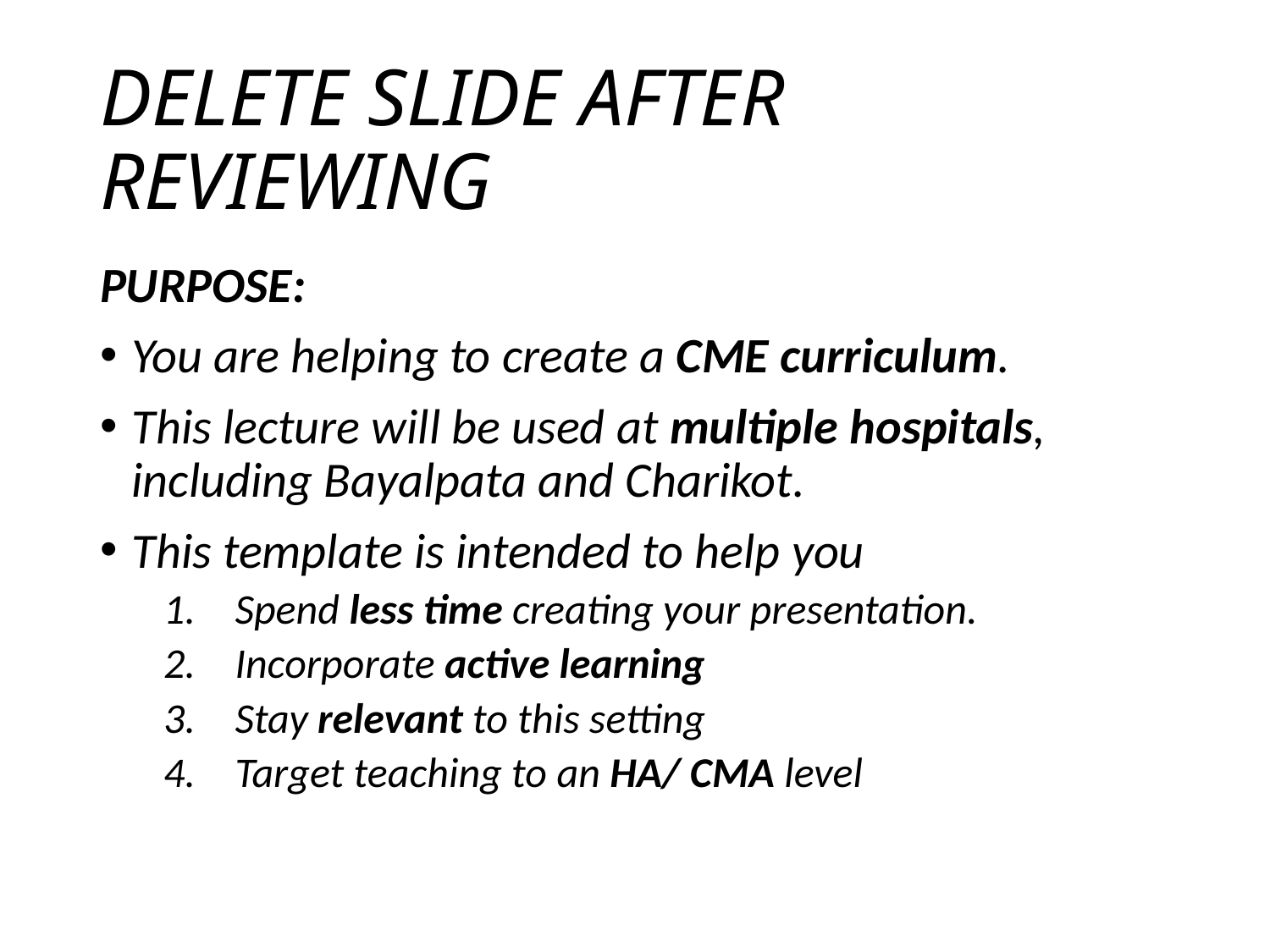

# DELETE SLIDE AFTER REVIEWING
PURPOSE:
You are helping to create a CME curriculum.
This lecture will be used at multiple hospitals, including Bayalpata and Charikot.
This template is intended to help you
Spend less time creating your presentation.
Incorporate active learning
Stay relevant to this setting
Target teaching to an HA/ CMA level

## Slide 2
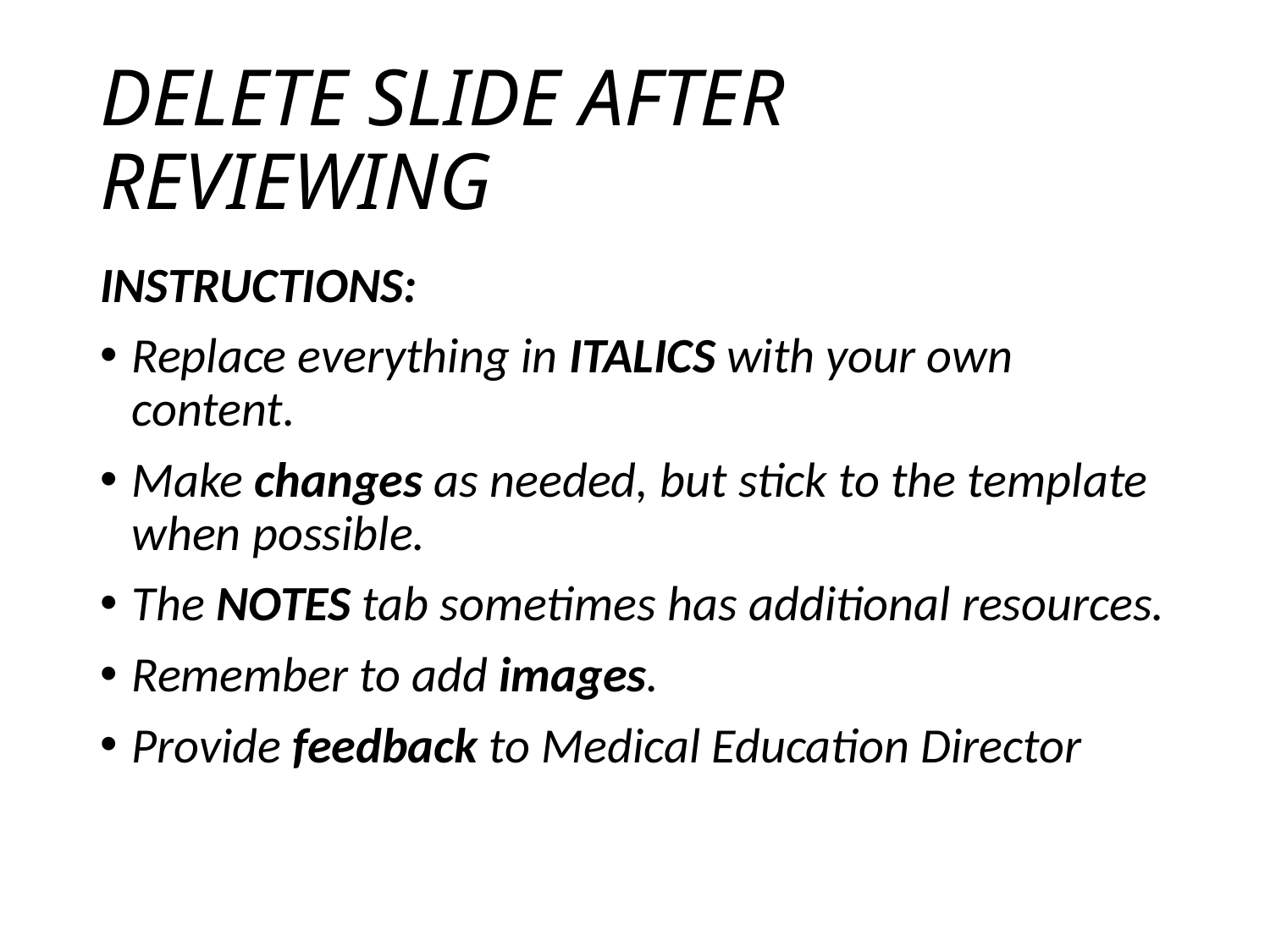

# DELETE SLIDE AFTER REVIEWING
INSTRUCTIONS:
Replace everything in ITALICS with your own content.
Make changes as needed, but stick to the template when possible.
The NOTES tab sometimes has additional resources.
Remember to add images.
Provide feedback to Medical Education Director

## Slide 3
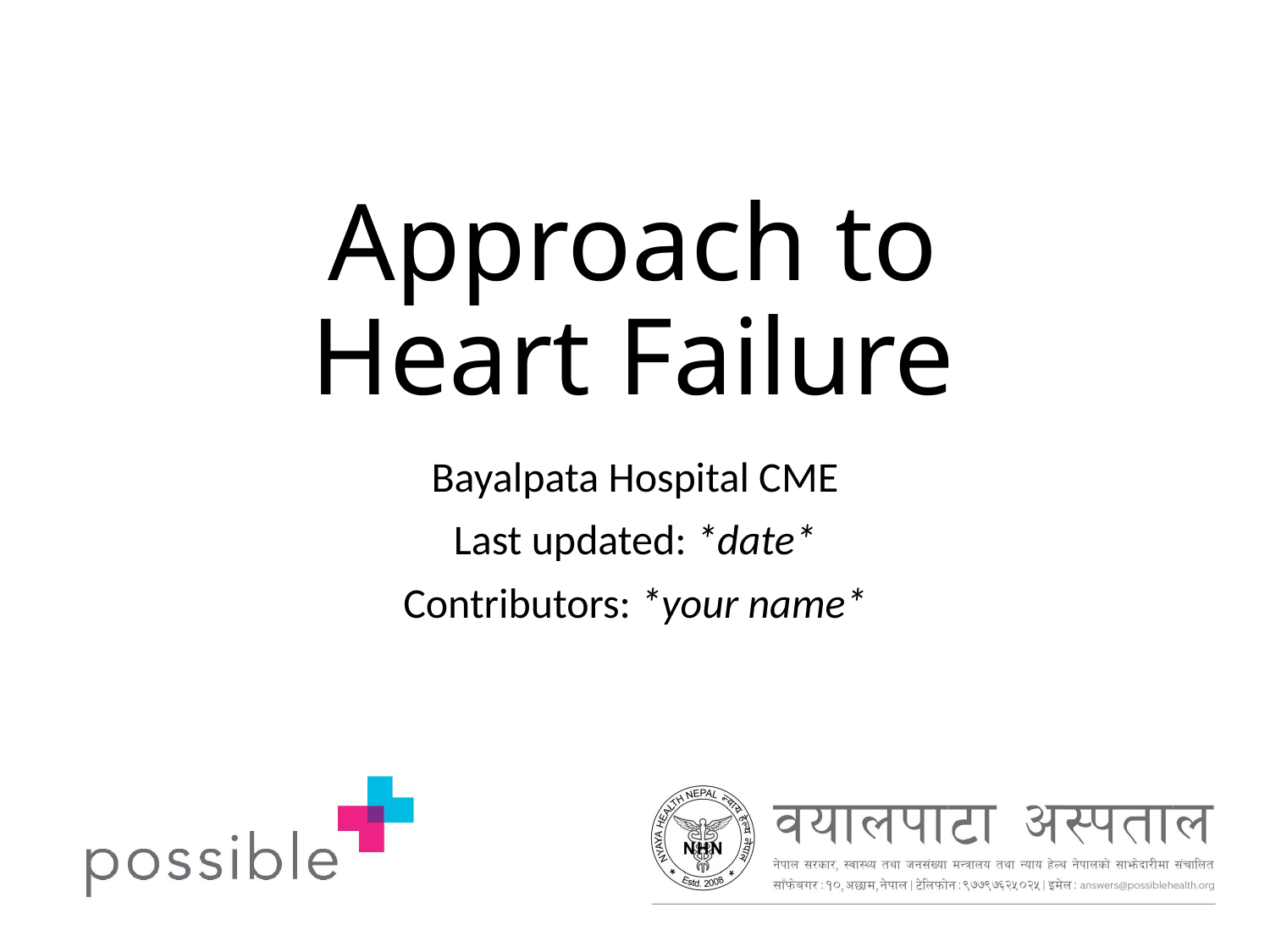

# Approach to Heart Failure
Bayalpata Hospital CME
Last updated: *date*
Contributors: *your name*

## Slide 4
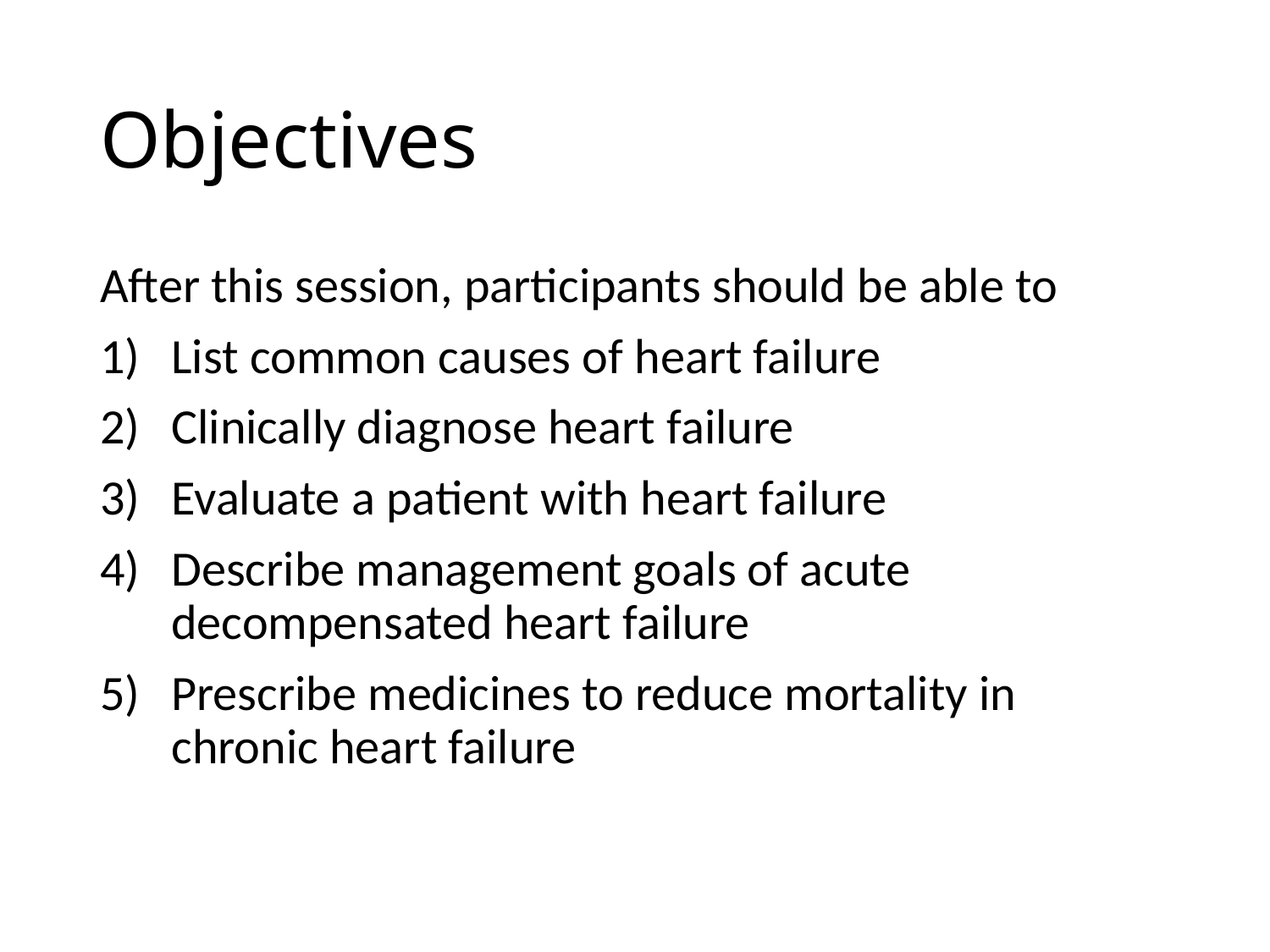

# Objectives
After this session, participants should be able to
List common causes of heart failure
Clinically diagnose heart failure
Evaluate a patient with heart failure
Describe management goals of acute decompensated heart failure
Prescribe medicines to reduce mortality in chronic heart failure

## Slide 5
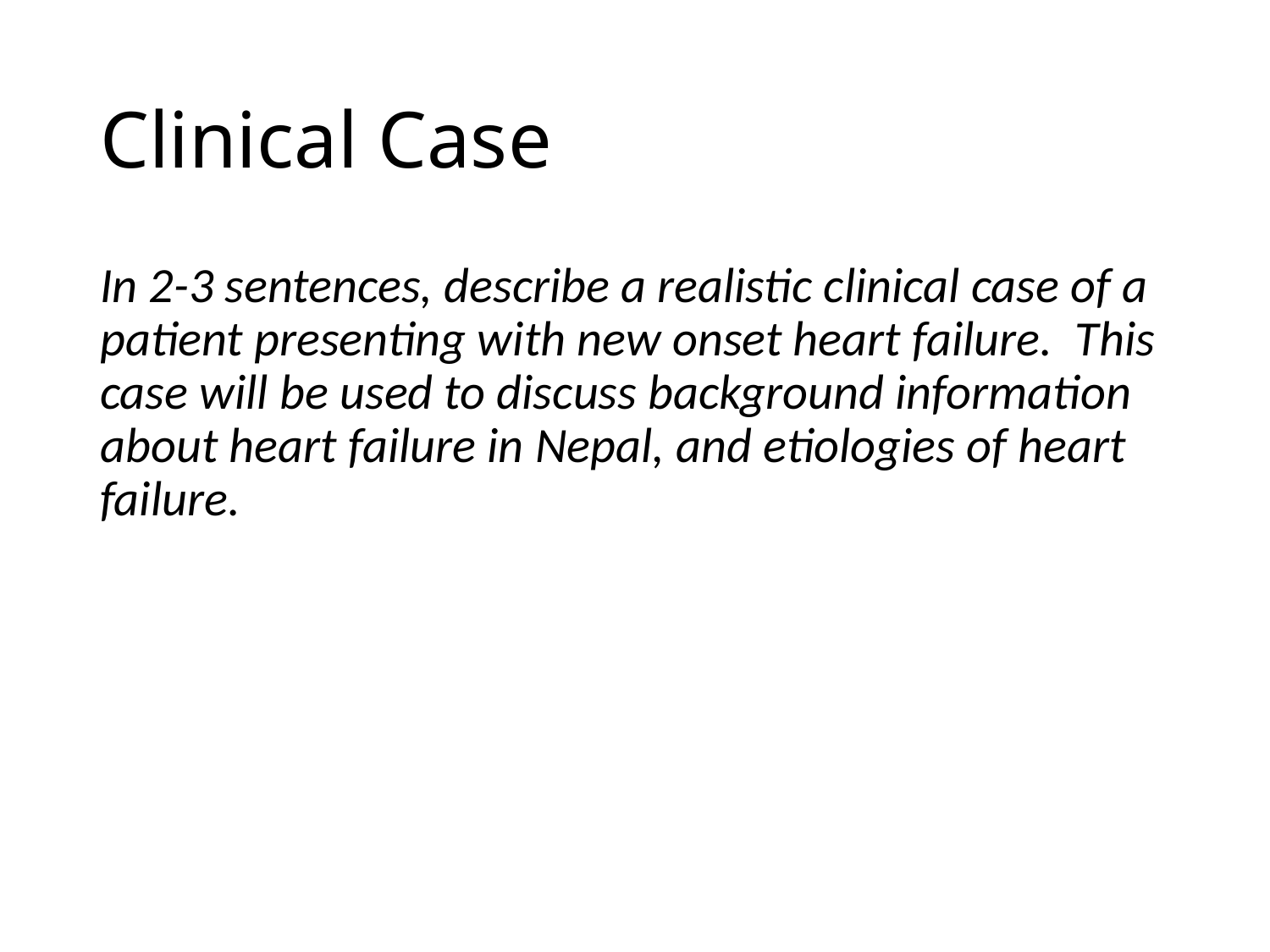

# Clinical Case
In 2-3 sentences, describe a realistic clinical case of a patient presenting with new onset heart failure. This case will be used to discuss background information about heart failure in Nepal, and etiologies of heart failure.

## Slide 6
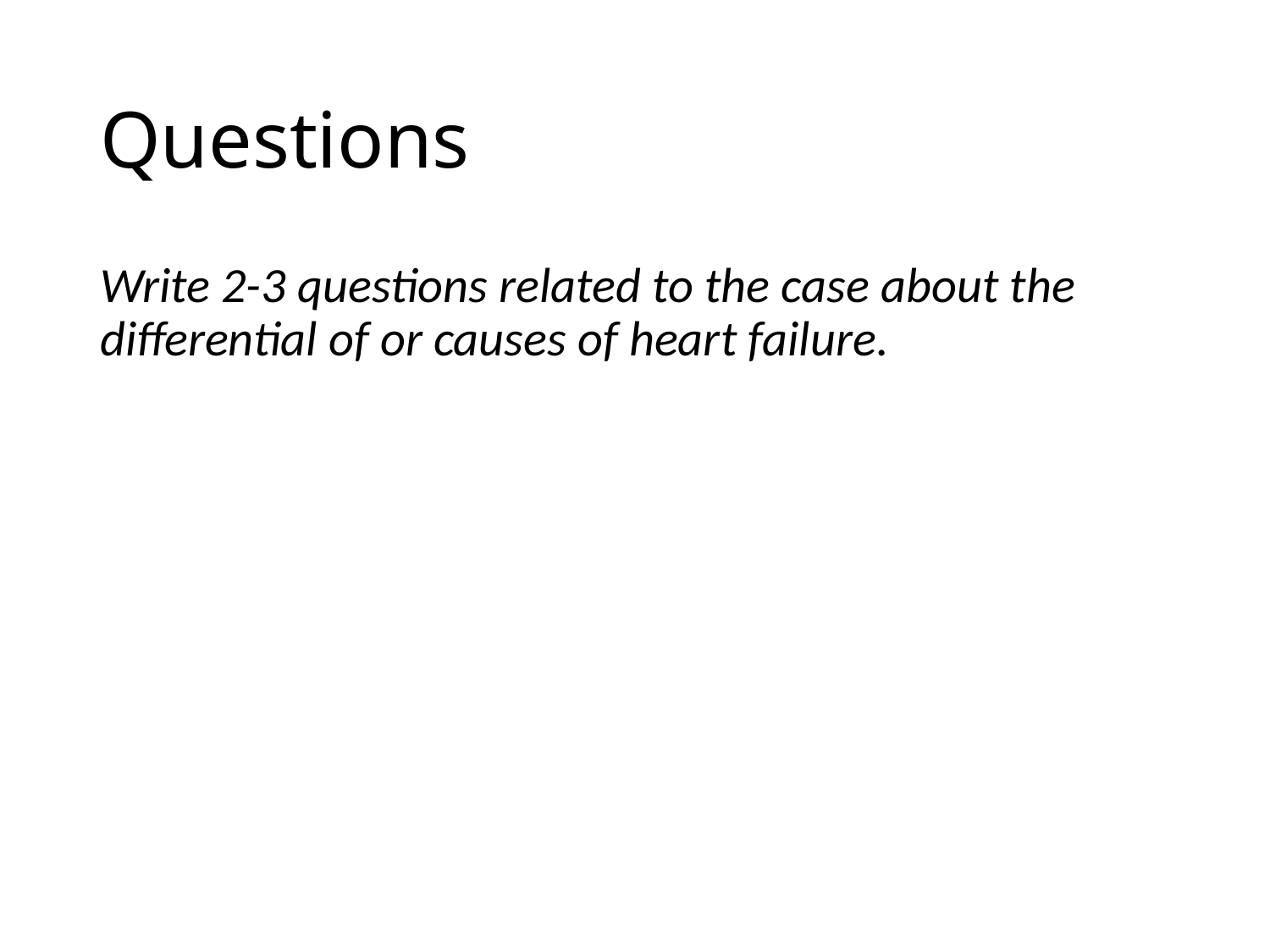

# Questions
Write 2-3 questions related to the case about the differential of or causes of heart failure.

## Slide 7
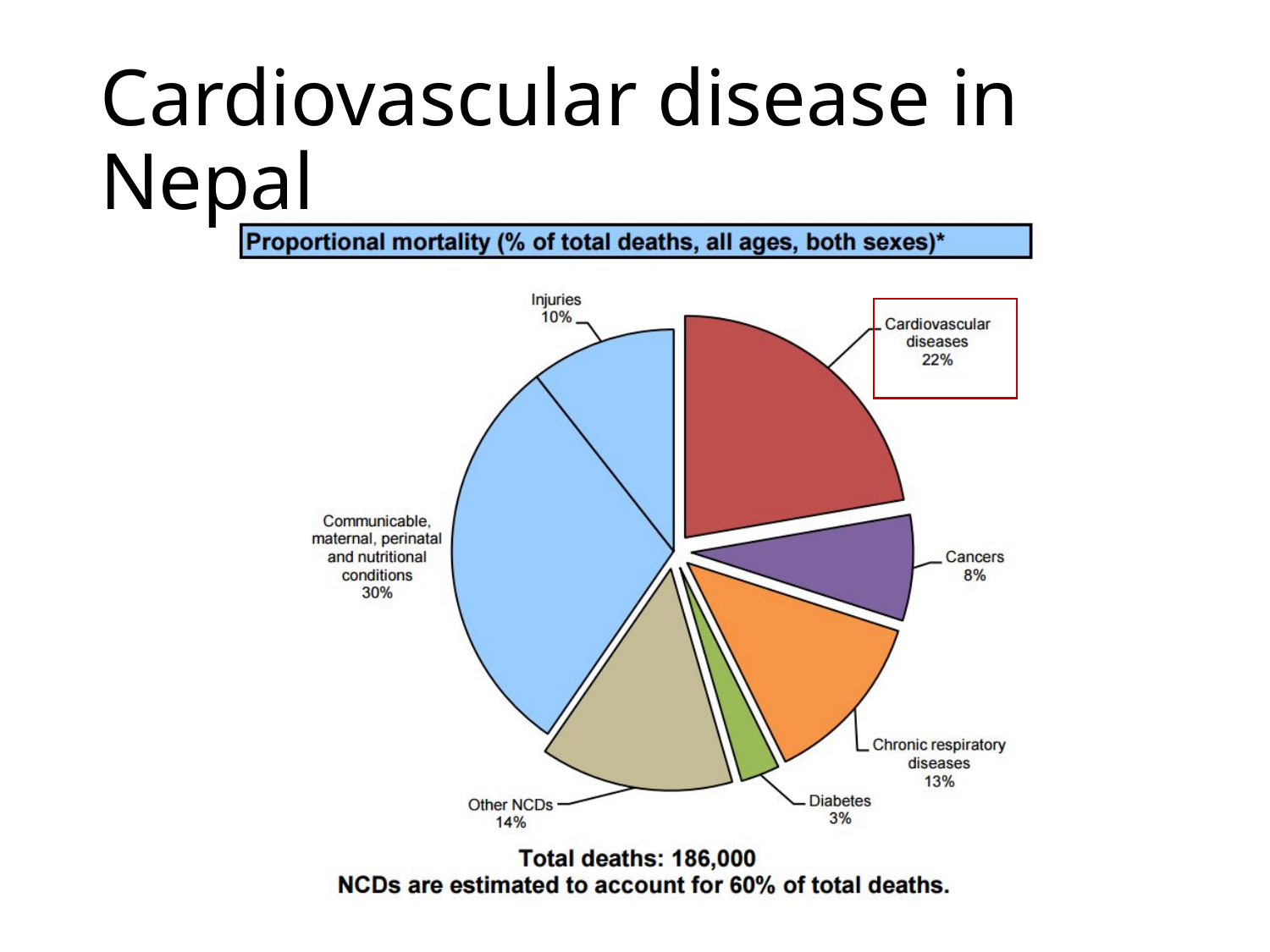

# Cardiovascular disease in Nepal

## Slide 8
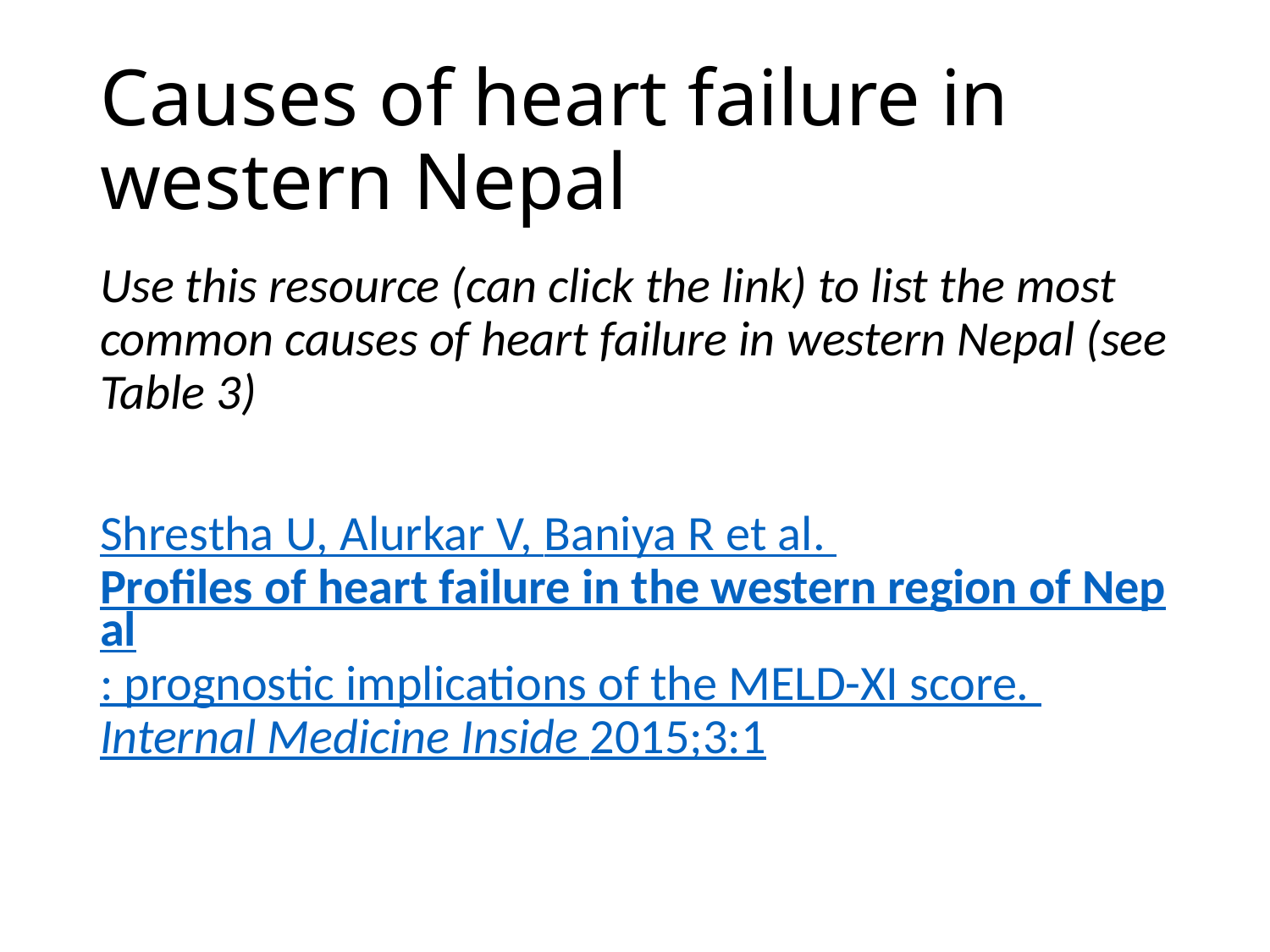

# Causes of heart failure in western Nepal
Use this resource (can click the link) to list the most common causes of heart failure in western Nepal (see Table 3)
Shrestha U, Alurkar V, Baniya R et al. Profiles of heart failure in the western region of Nepal: prognostic implications of the MELD-XI score. Internal Medicine Inside 2015;3:1

## Slide 9
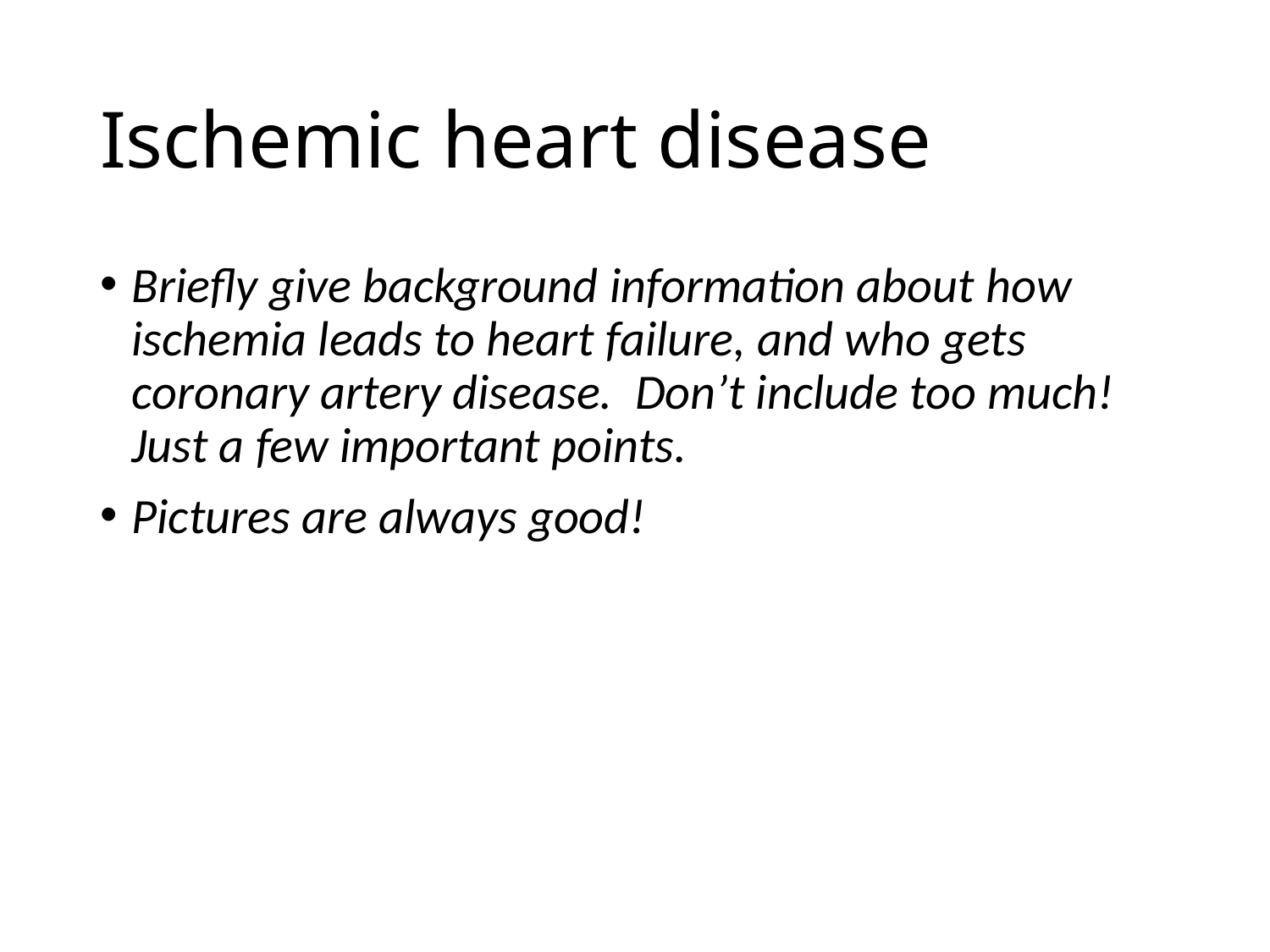

# Ischemic heart disease
Briefly give background information about how ischemia leads to heart failure, and who gets coronary artery disease. Don’t include too much! Just a few important points.
Pictures are always good!

## Slide 10
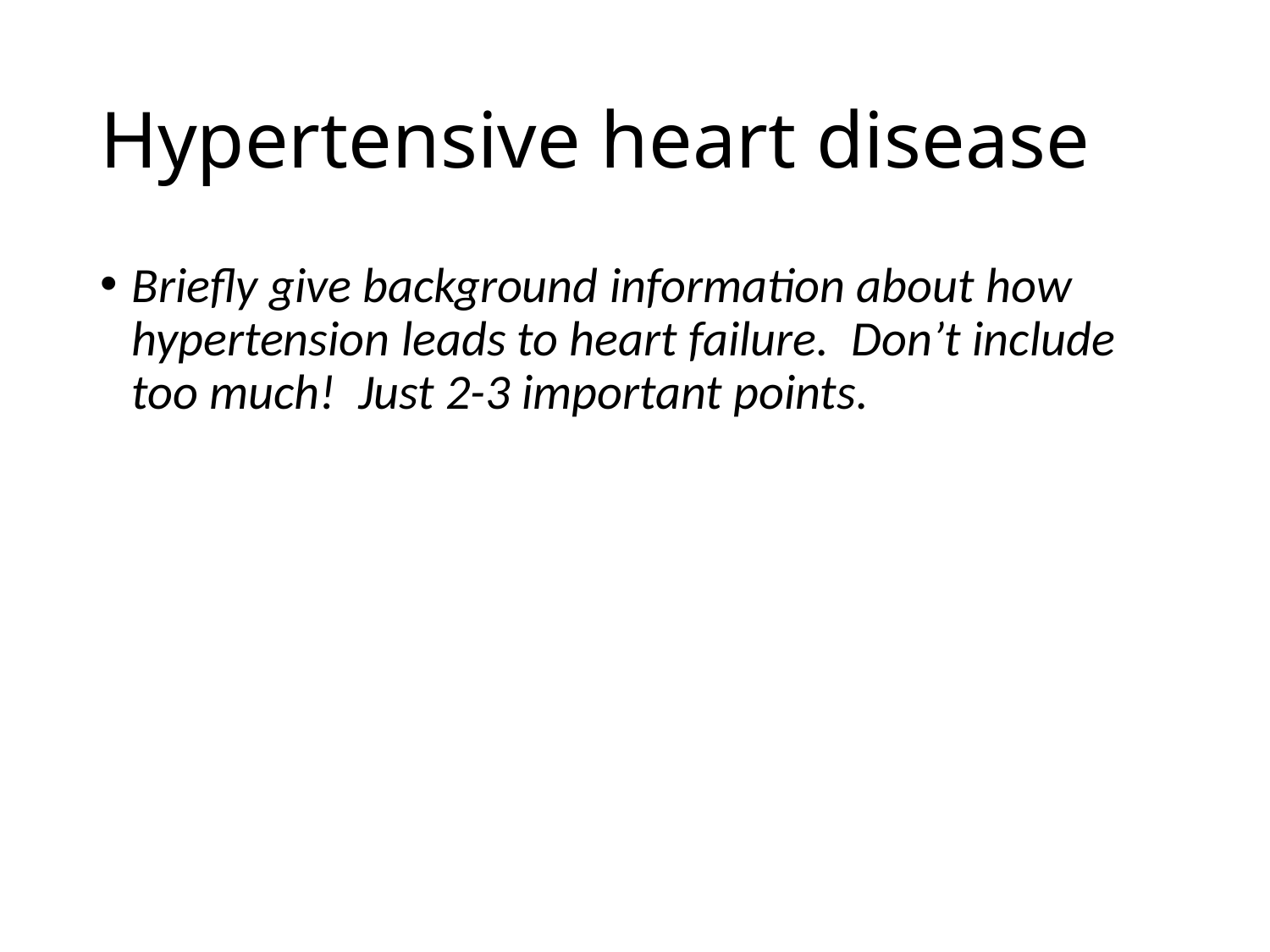

# Hypertensive heart disease
Briefly give background information about how hypertension leads to heart failure. Don’t include too much! Just 2-3 important points.

## Slide 11
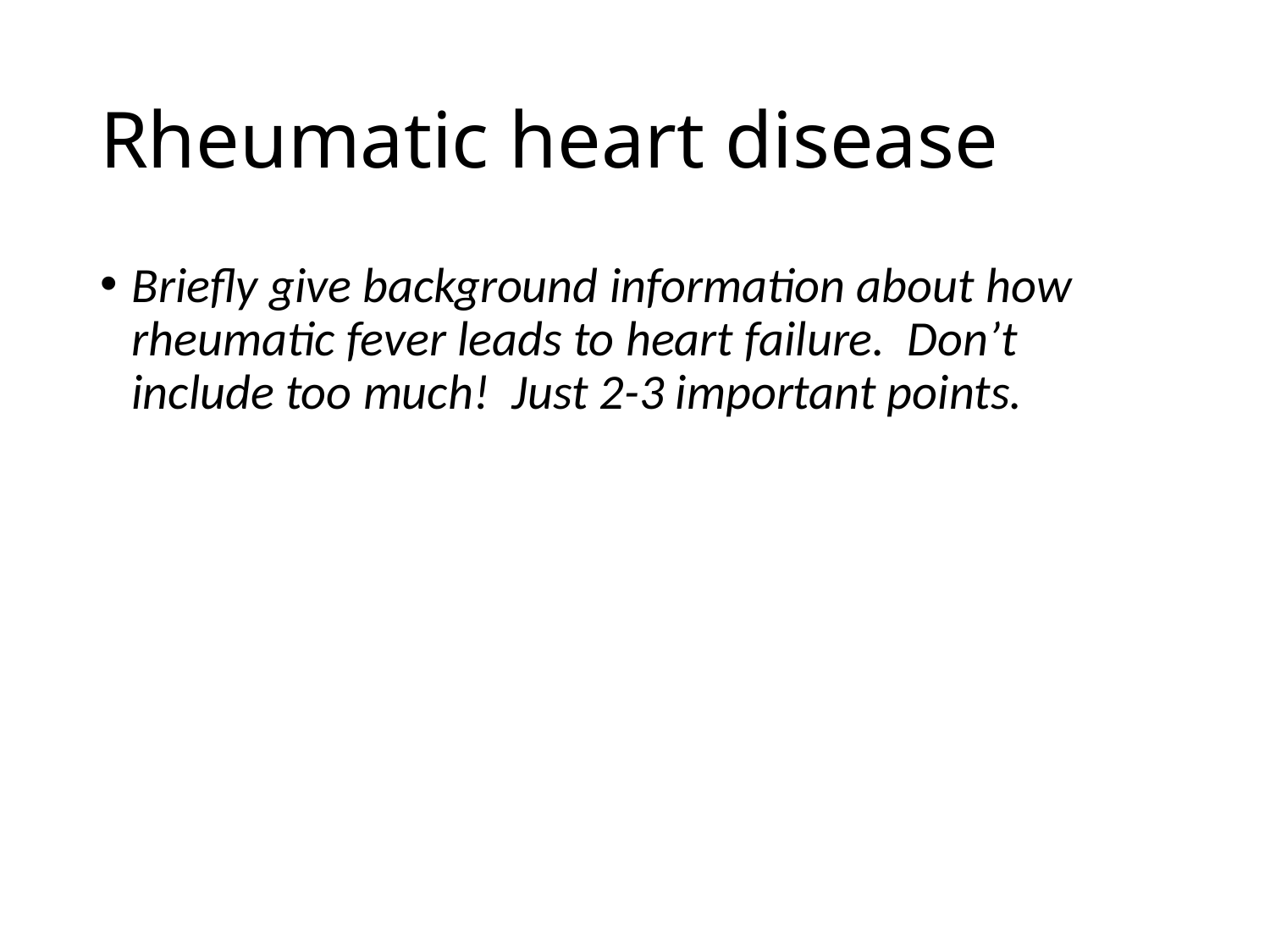

# Rheumatic heart disease
Briefly give background information about how rheumatic fever leads to heart failure. Don’t include too much! Just 2-3 important points.

## Slide 12
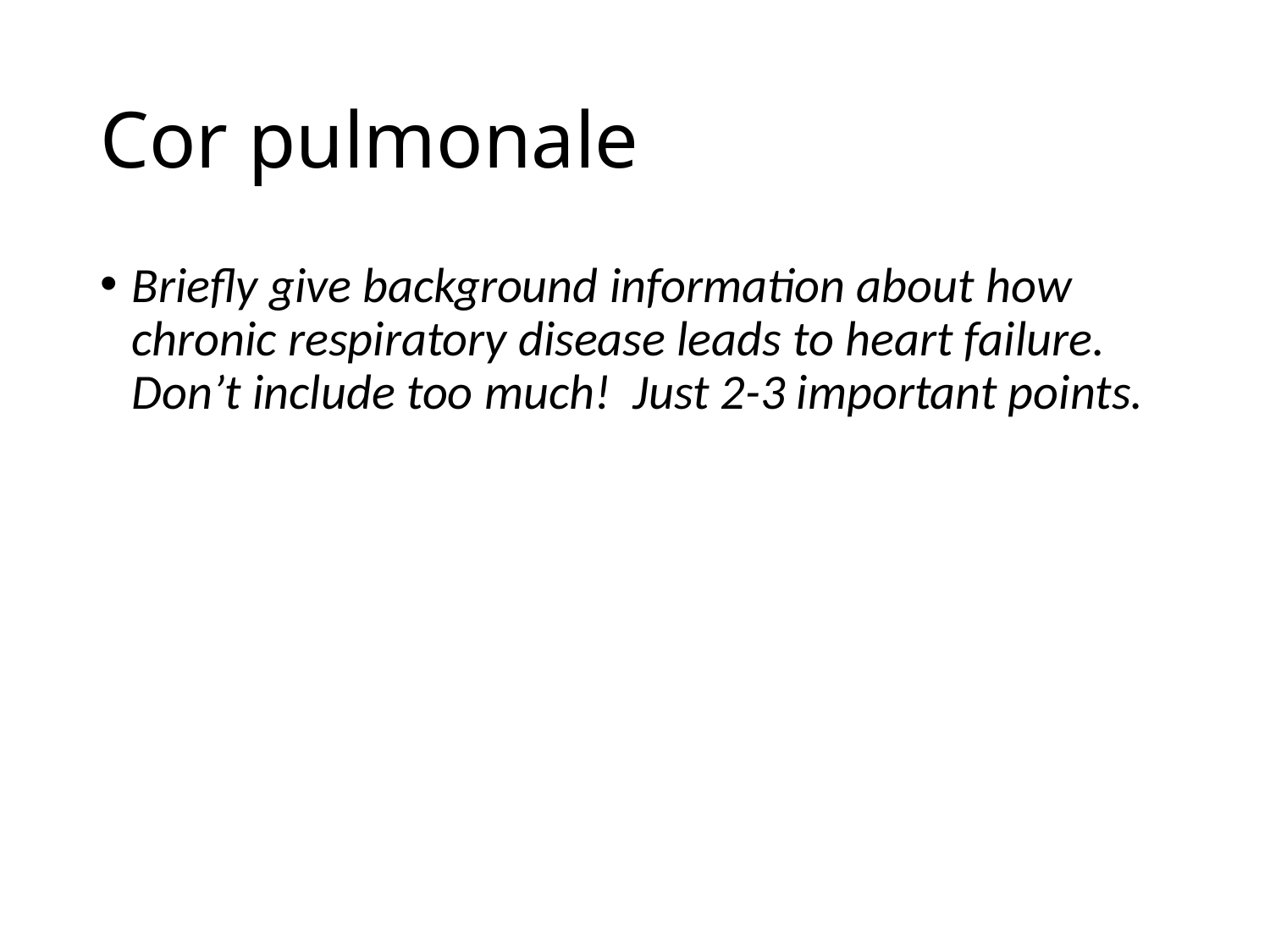

# Cor pulmonale
Briefly give background information about how chronic respiratory disease leads to heart failure. Don’t include too much! Just 2-3 important points.

## Slide 13
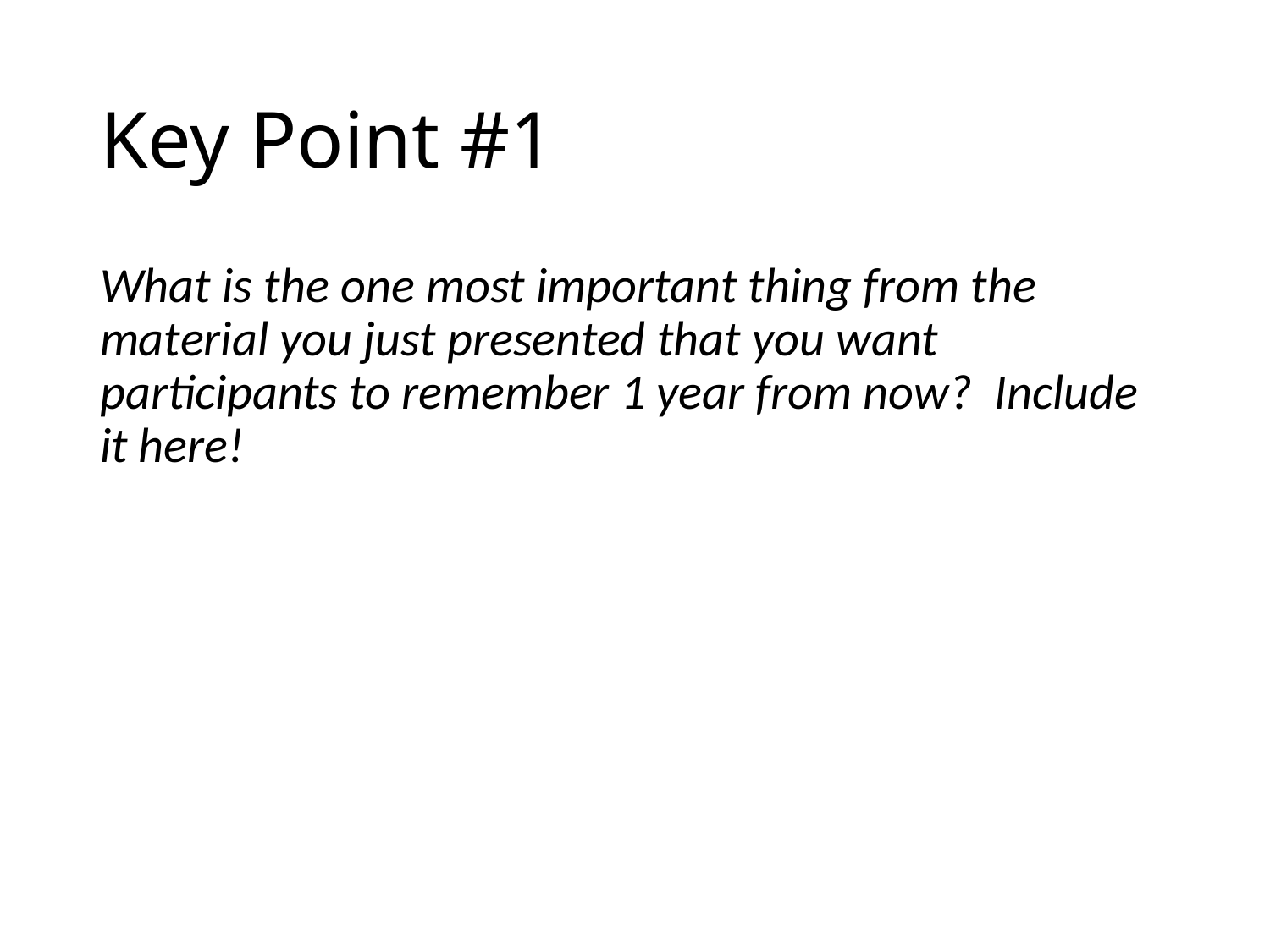

# Key Point #1
What is the one most important thing from the material you just presented that you want participants to remember 1 year from now? Include it here!

## Slide 14
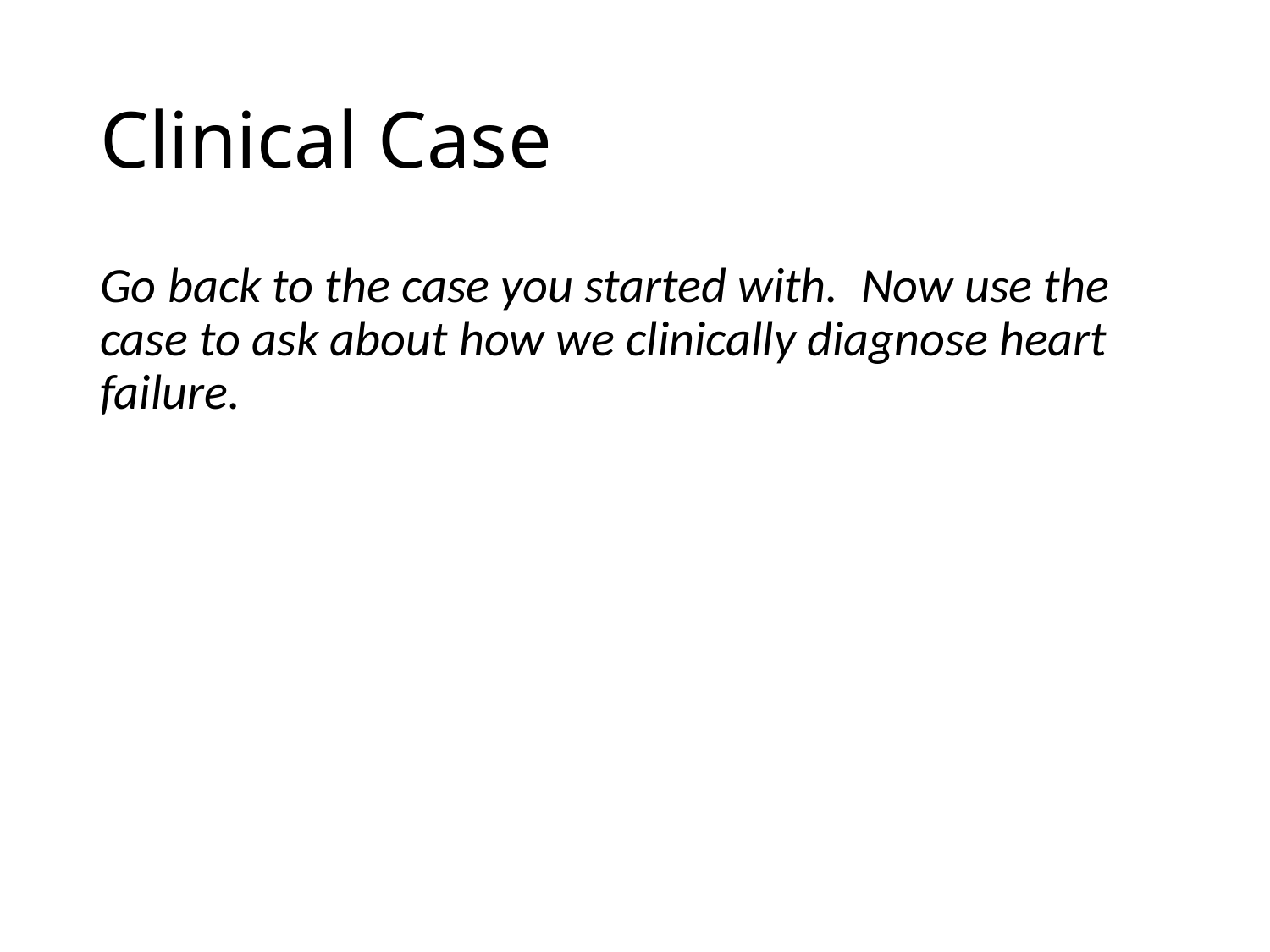

# Clinical Case
Go back to the case you started with. Now use the case to ask about how we clinically diagnose heart failure.

## Slide 15
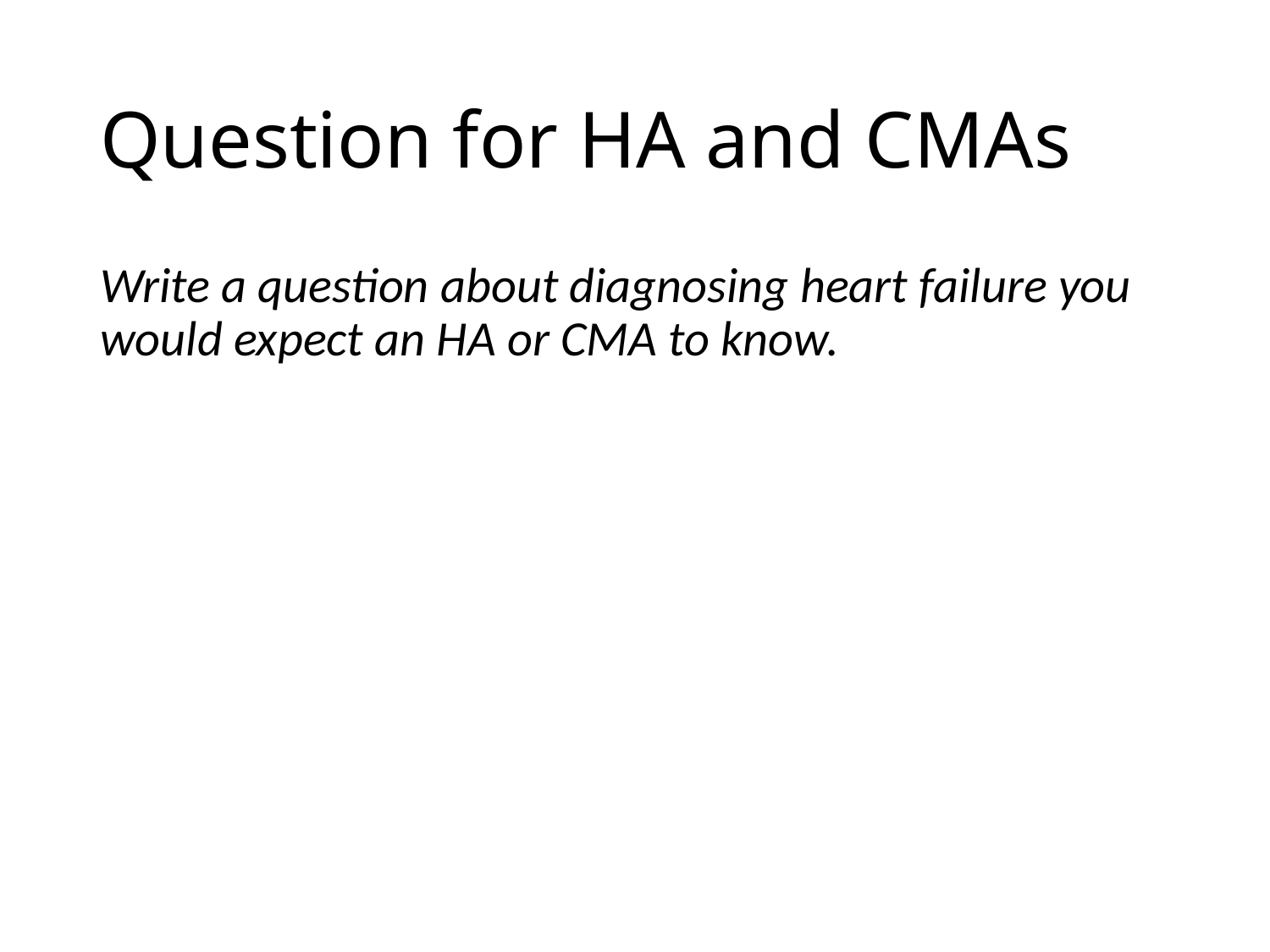

# Question for HA and CMAs
Write a question about diagnosing heart failure you would expect an HA or CMA to know.

## Slide 16
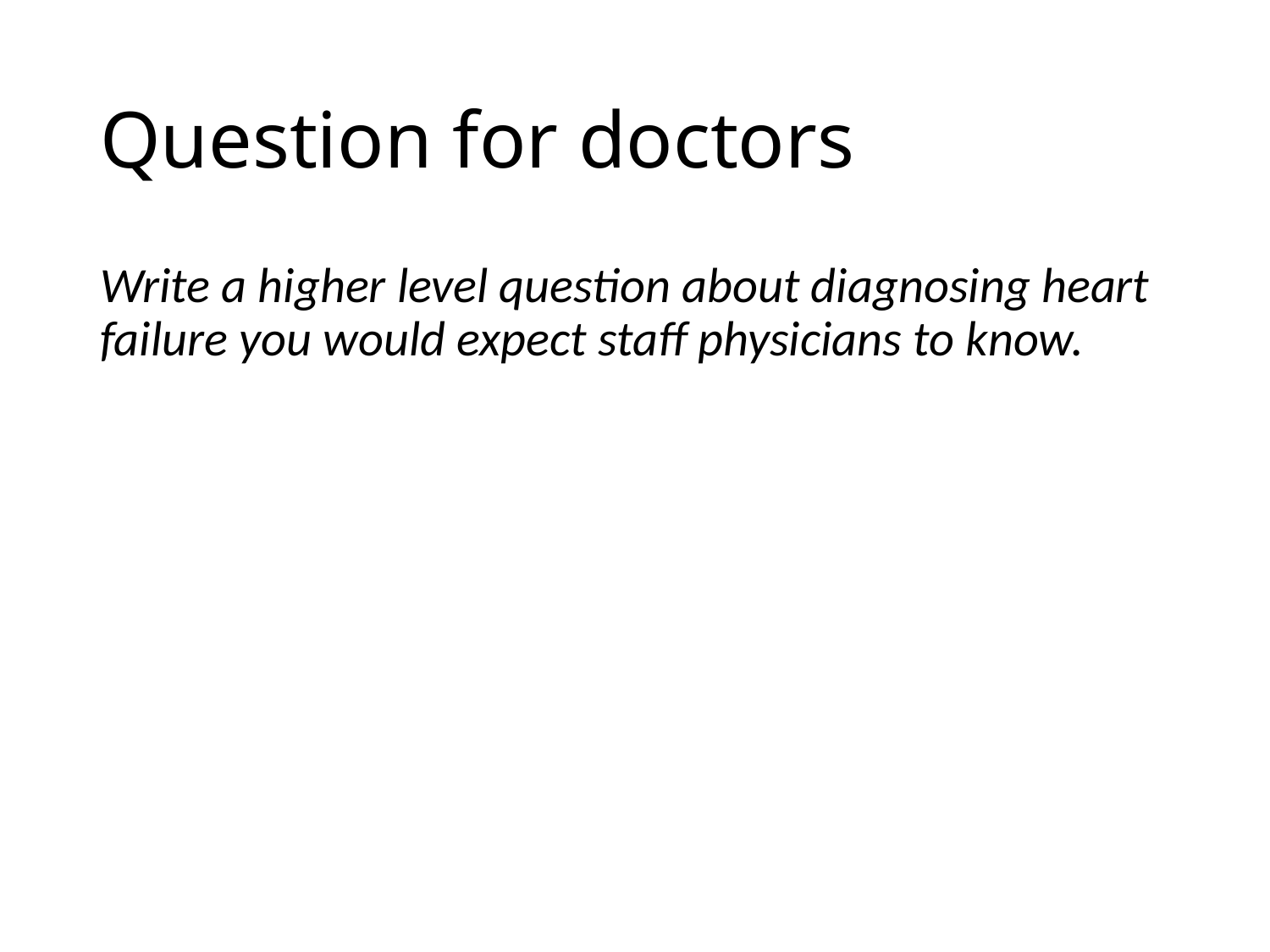

# Question for doctors
Write a higher level question about diagnosing heart failure you would expect staff physicians to know.

## Slide 17
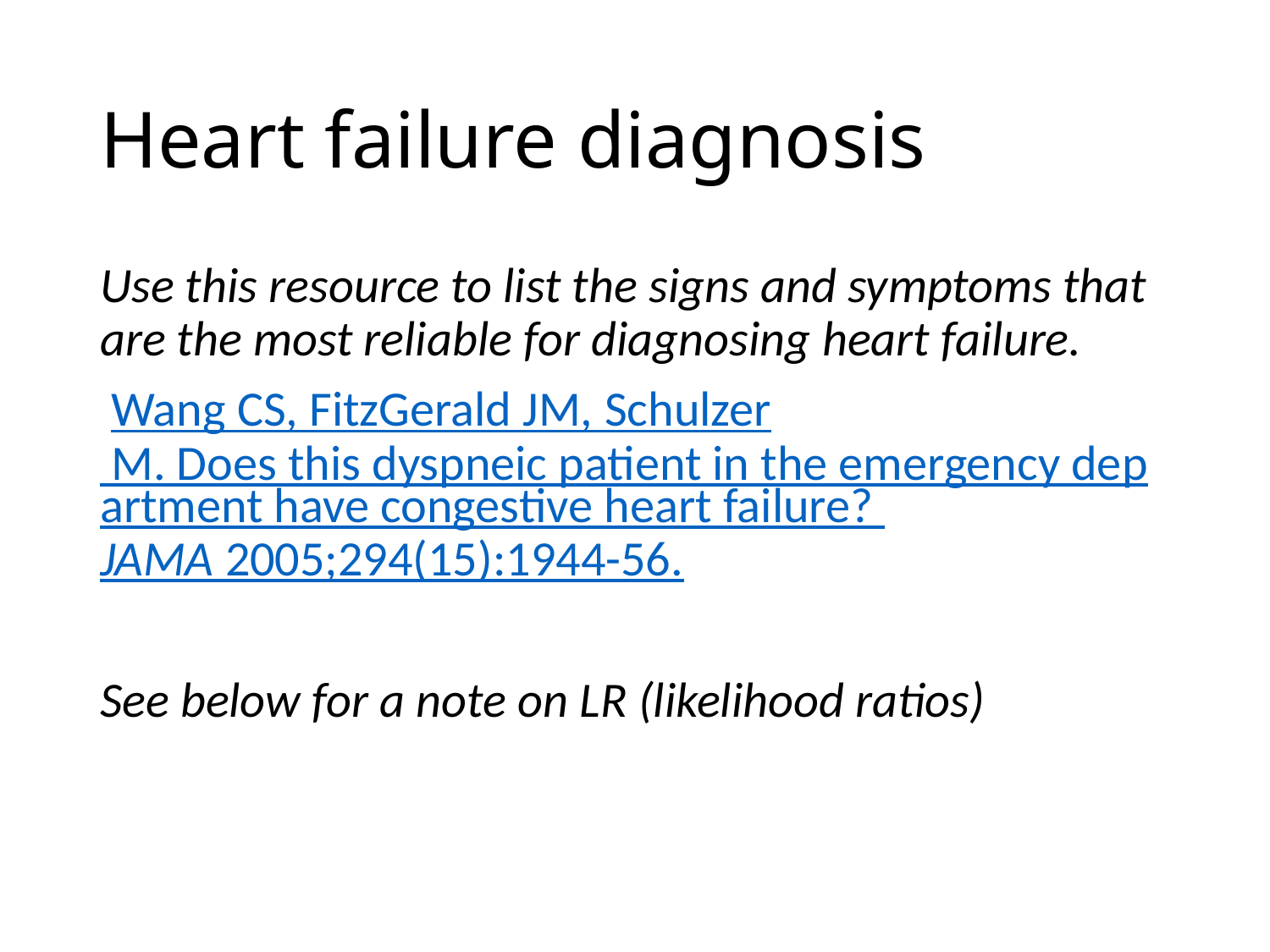

# Heart failure diagnosis
Use this resource to list the signs and symptoms that are the most reliable for diagnosing heart failure.
 Wang CS, FitzGerald JM, Schulzer M. Does this dyspneic patient in the emergency department have congestive heart failure? JAMA 2005;294(15):1944-56.
See below for a note on LR (likelihood ratios)

## Slide 18
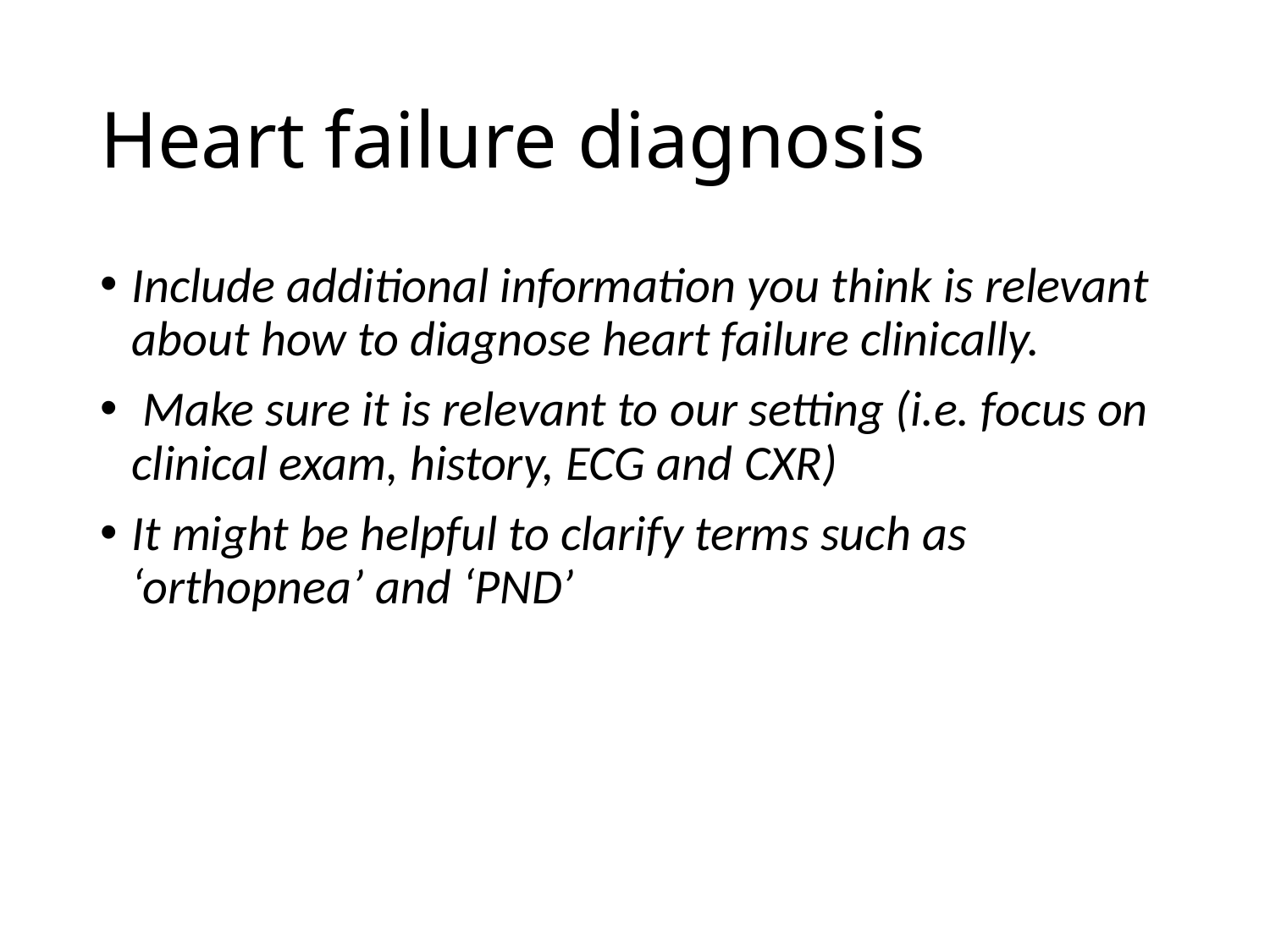

# Heart failure diagnosis
Include additional information you think is relevant about how to diagnose heart failure clinically.
 Make sure it is relevant to our setting (i.e. focus on clinical exam, history, ECG and CXR)
It might be helpful to clarify terms such as ‘orthopnea’ and ‘PND’

## Slide 19
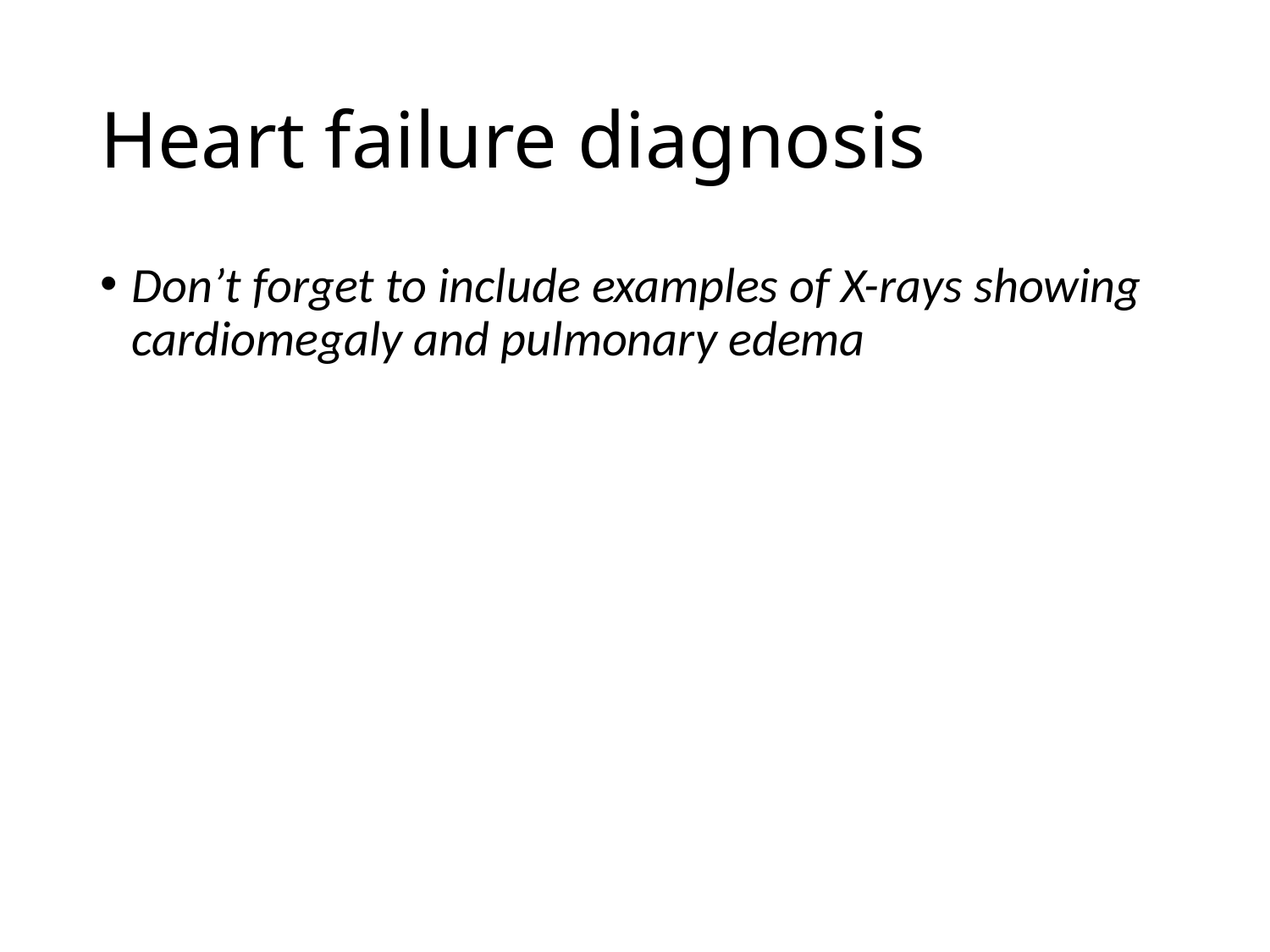

# Heart failure diagnosis
Don’t forget to include examples of X-rays showing cardiomegaly and pulmonary edema

## Slide 20
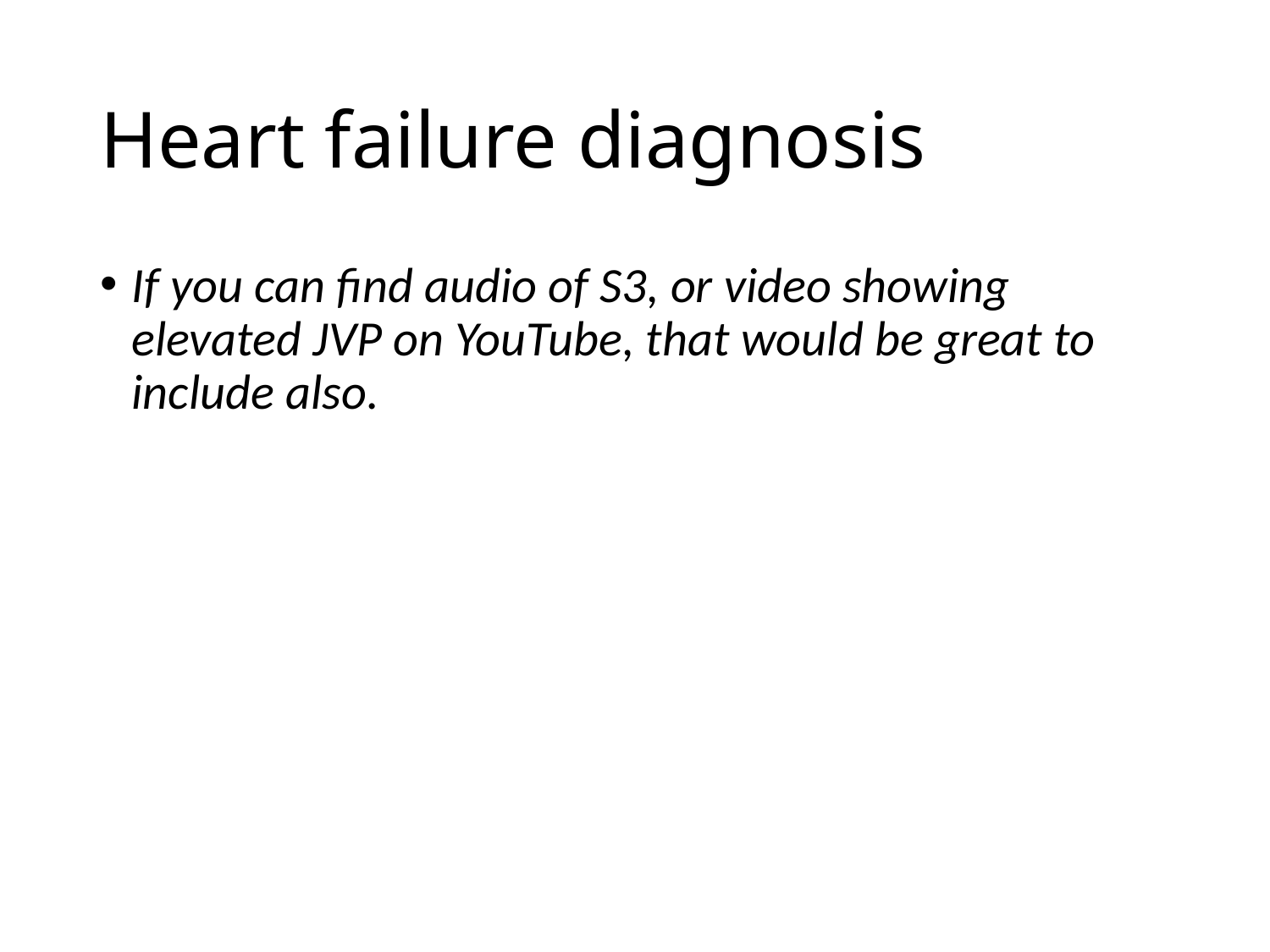

# Heart failure diagnosis
If you can find audio of S3, or video showing elevated JVP on YouTube, that would be great to include also.

## Slide 21
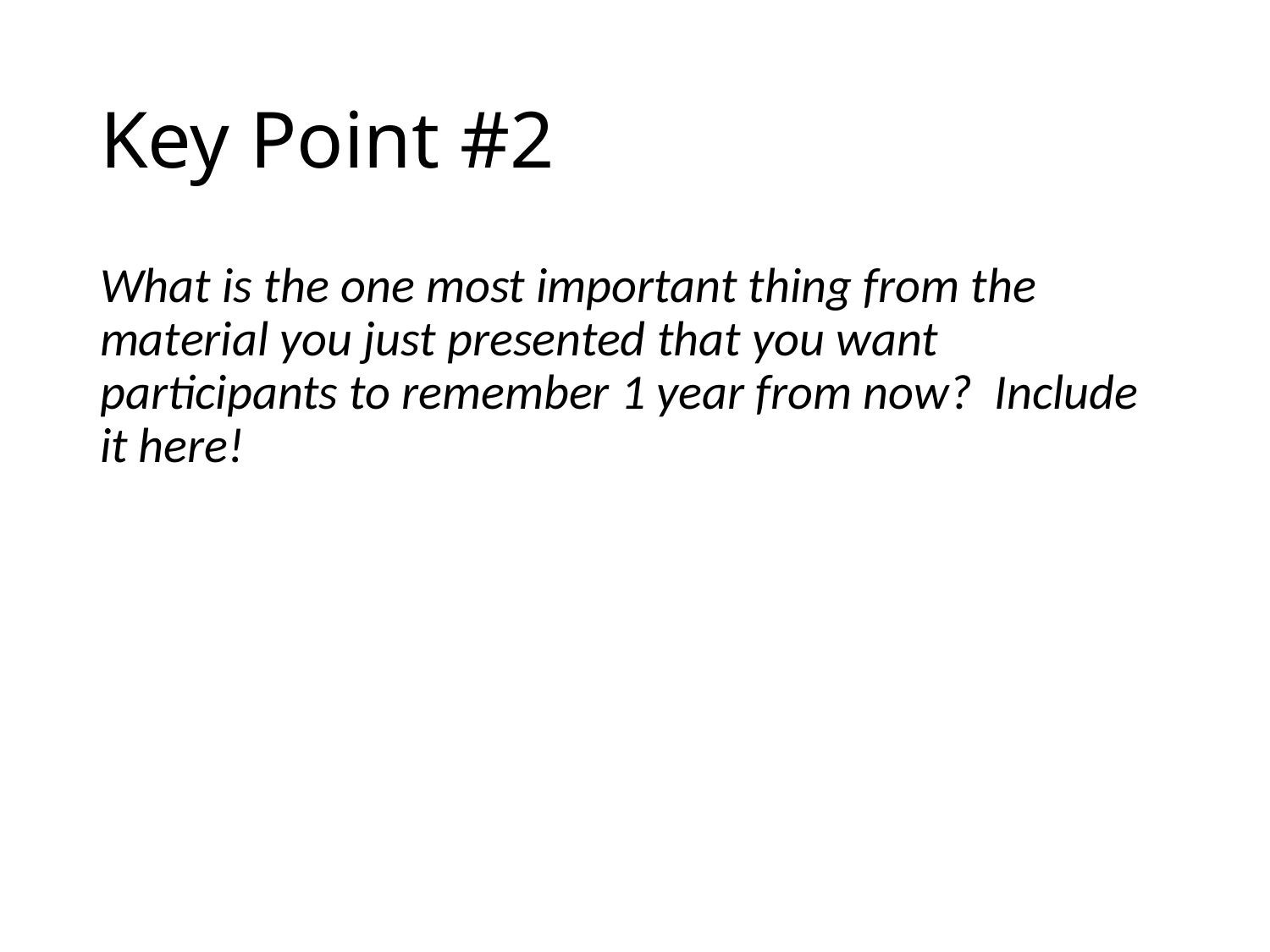

# Key Point #2
What is the one most important thing from the material you just presented that you want participants to remember 1 year from now? Include it here!

## Slide 22
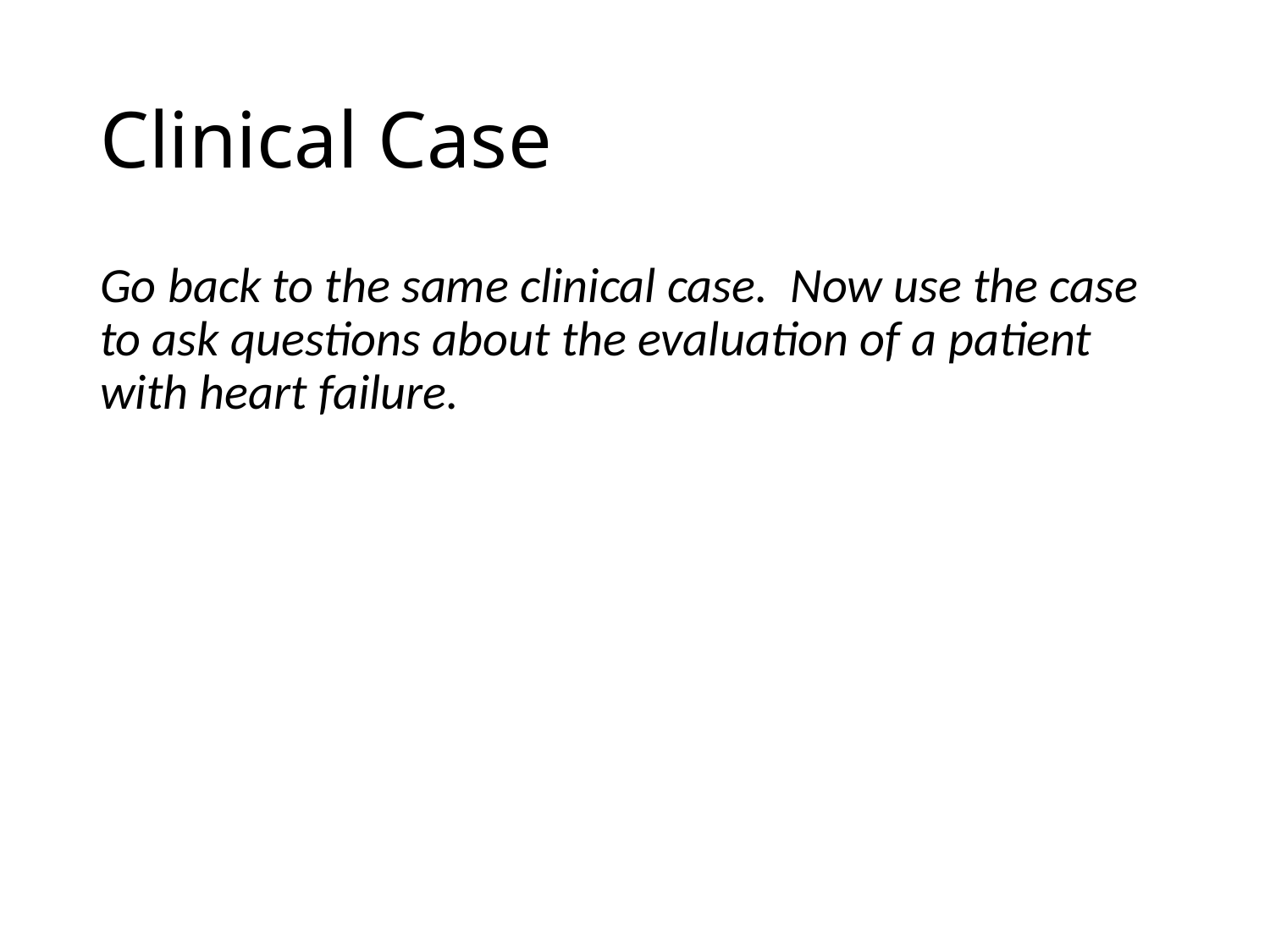

# Clinical Case
Go back to the same clinical case. Now use the case to ask questions about the evaluation of a patient with heart failure.

## Slide 23
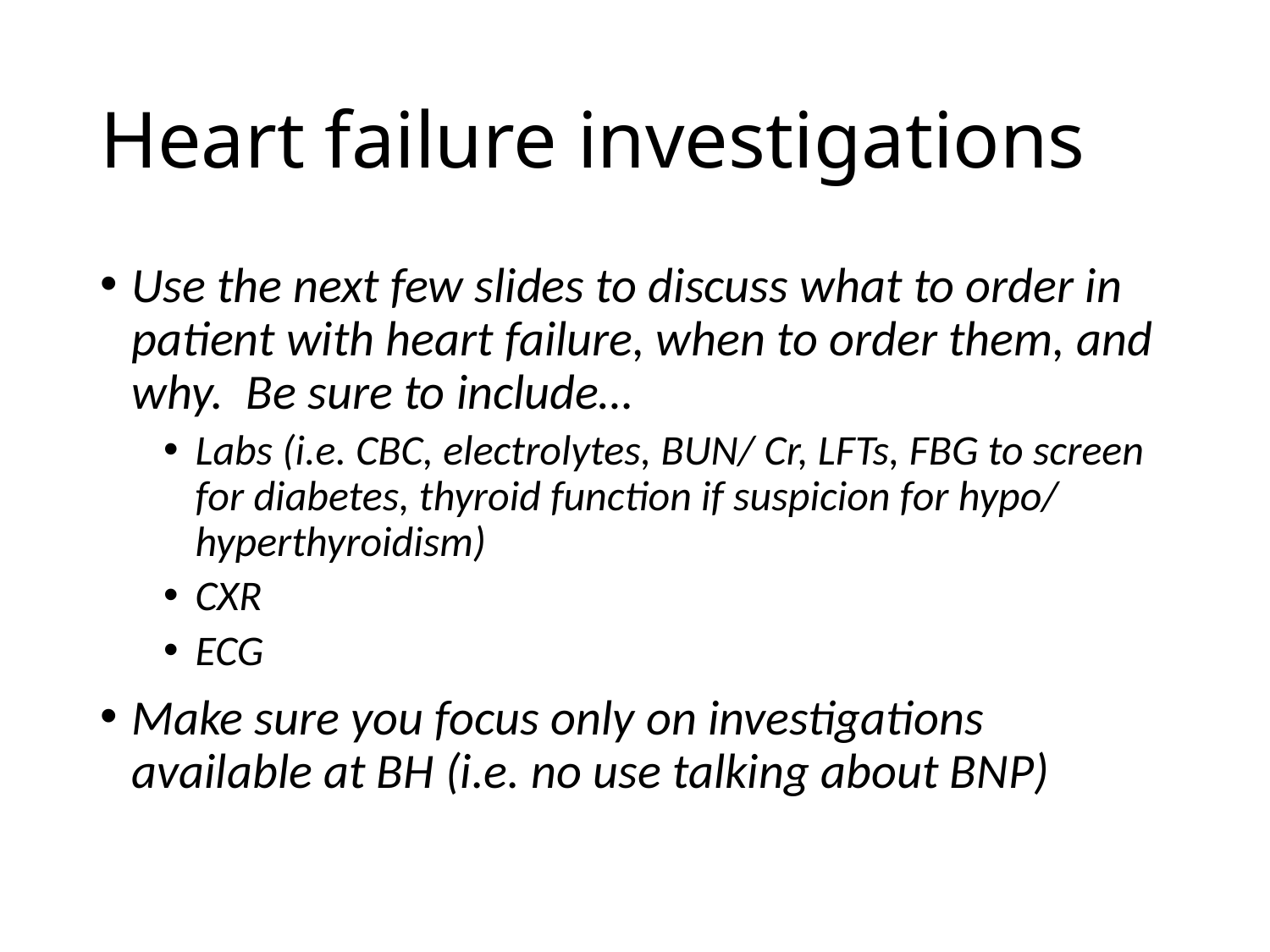

# Heart failure investigations
Use the next few slides to discuss what to order in patient with heart failure, when to order them, and why. Be sure to include…
Labs (i.e. CBC, electrolytes, BUN/ Cr, LFTs, FBG to screen for diabetes, thyroid function if suspicion for hypo/ hyperthyroidism)
CXR
ECG
Make sure you focus only on investigations available at BH (i.e. no use talking about BNP)

## Slide 24
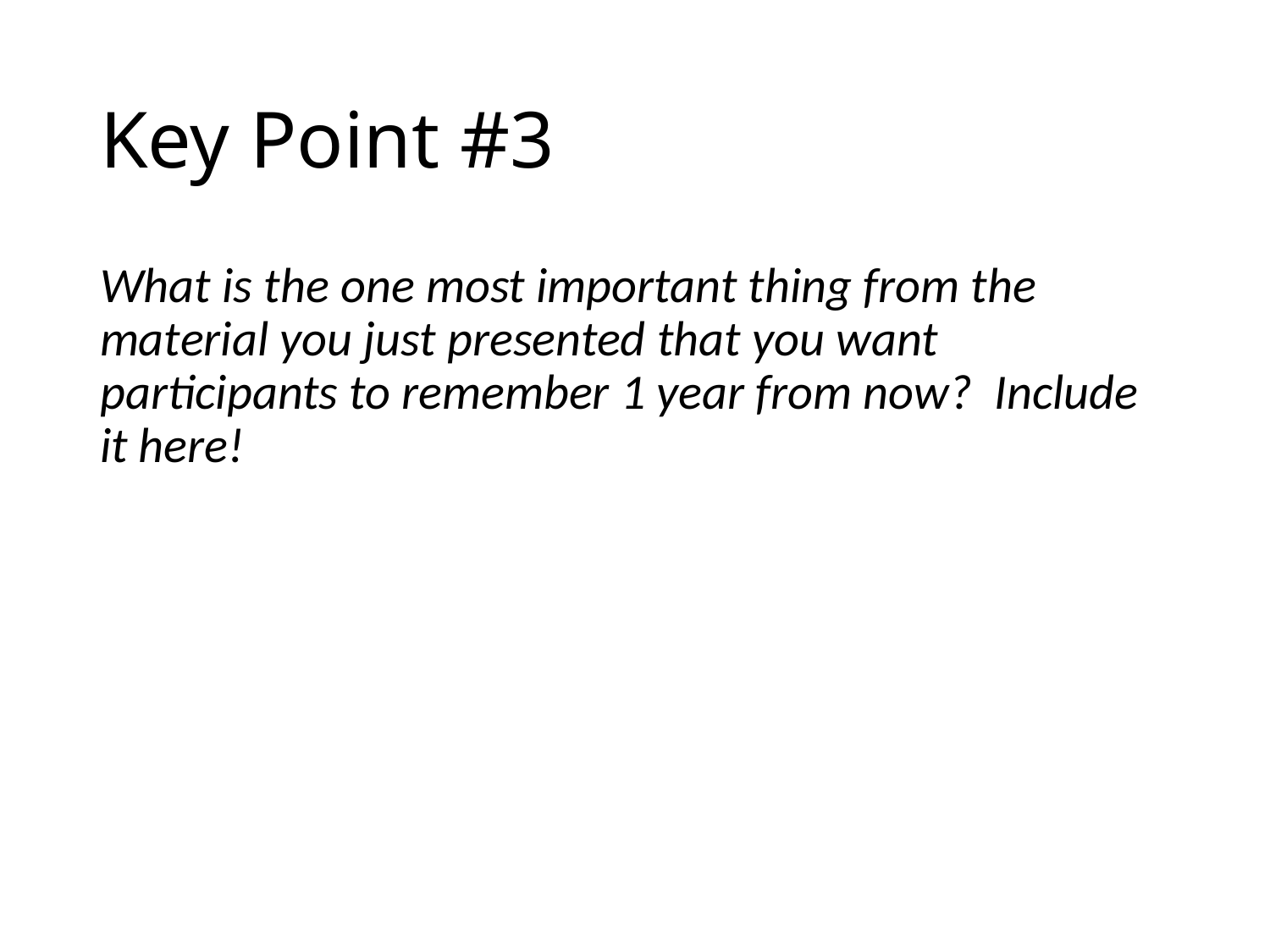

# Key Point #3
What is the one most important thing from the material you just presented that you want participants to remember 1 year from now? Include it here!

## Slide 25
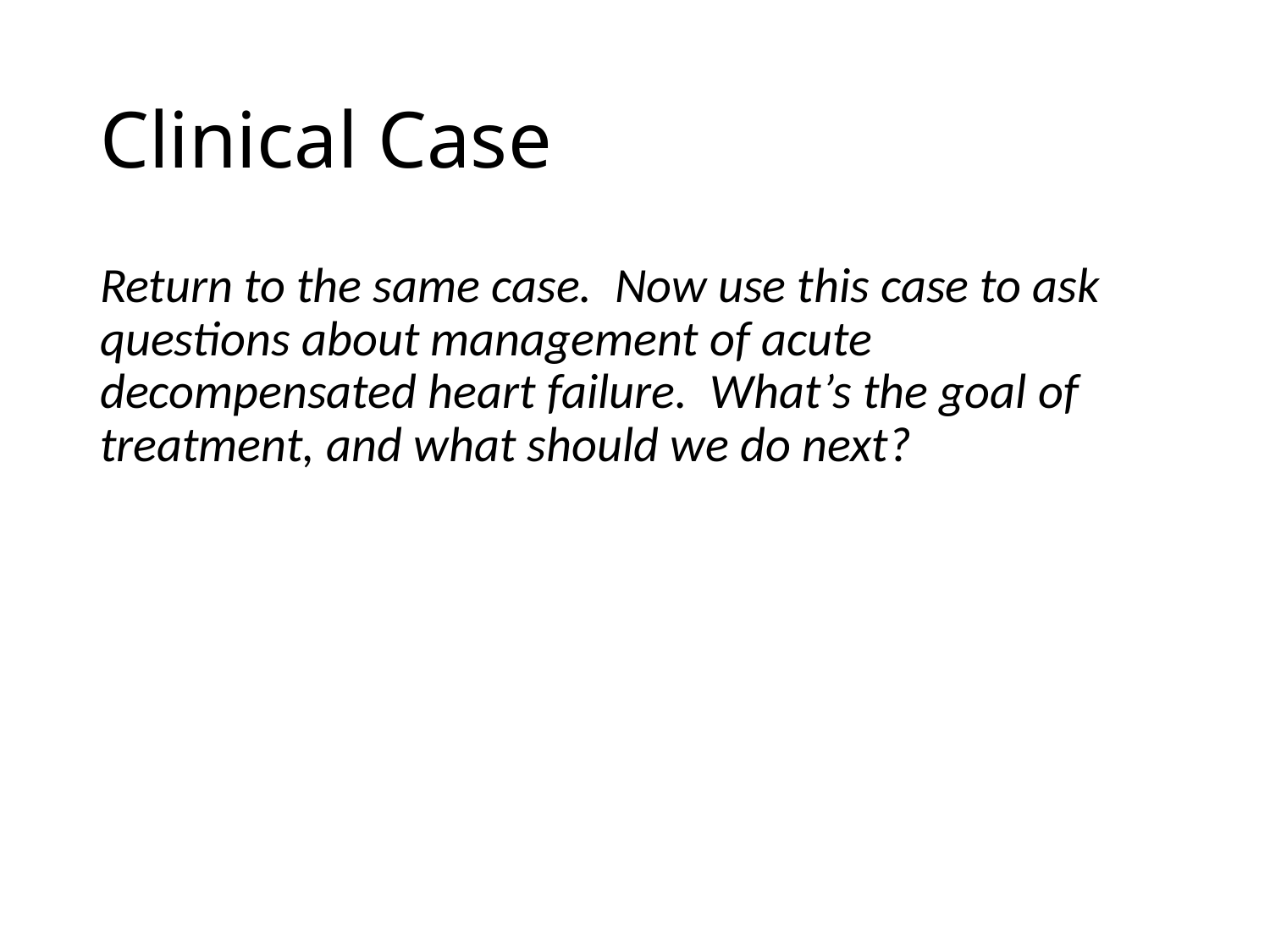

# Clinical Case
Return to the same case. Now use this case to ask questions about management of acute decompensated heart failure. What’s the goal of treatment, and what should we do next?

## Slide 26
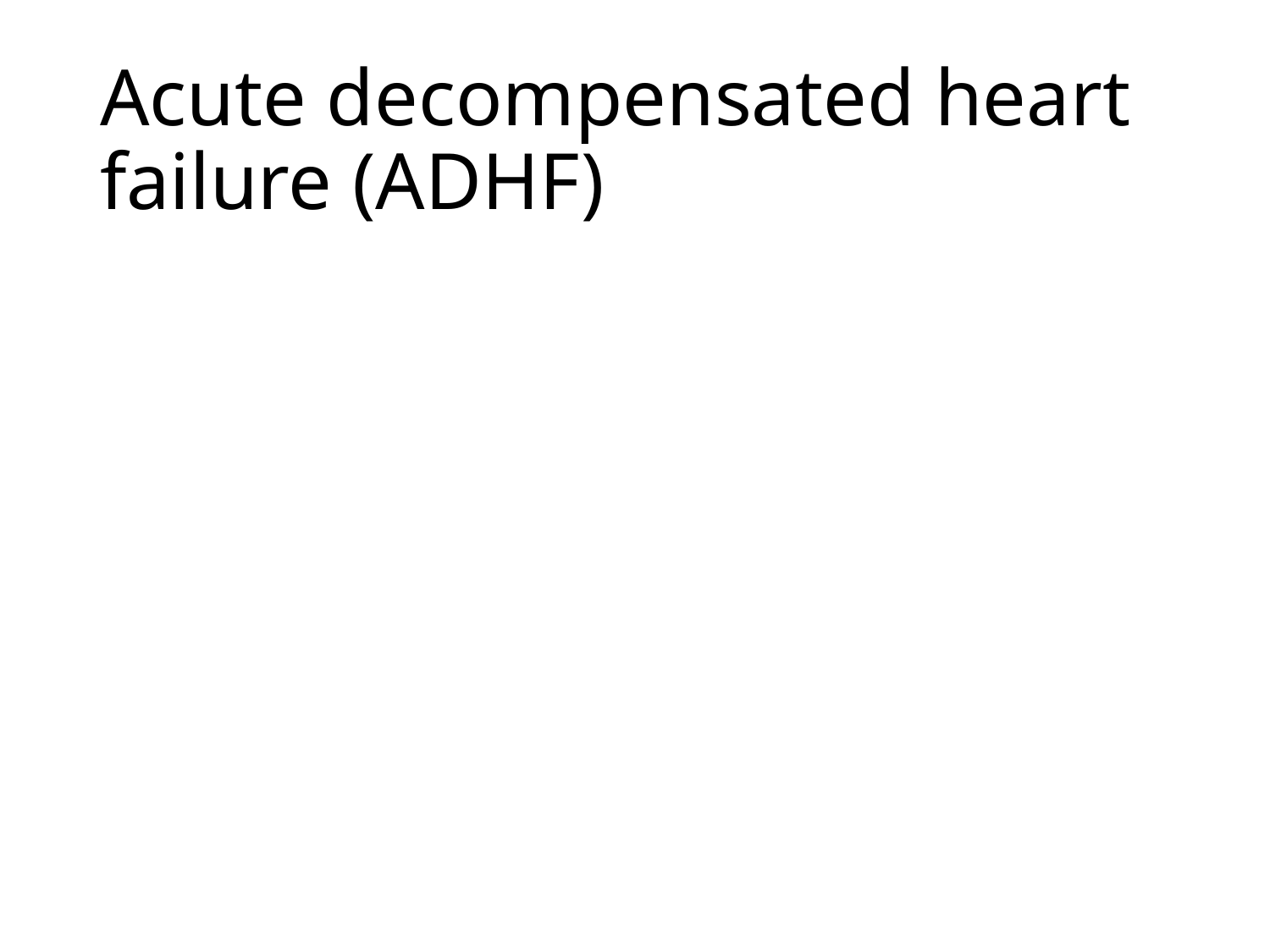

# Acute decompensated heart failure (ADHF)

## Slide 27
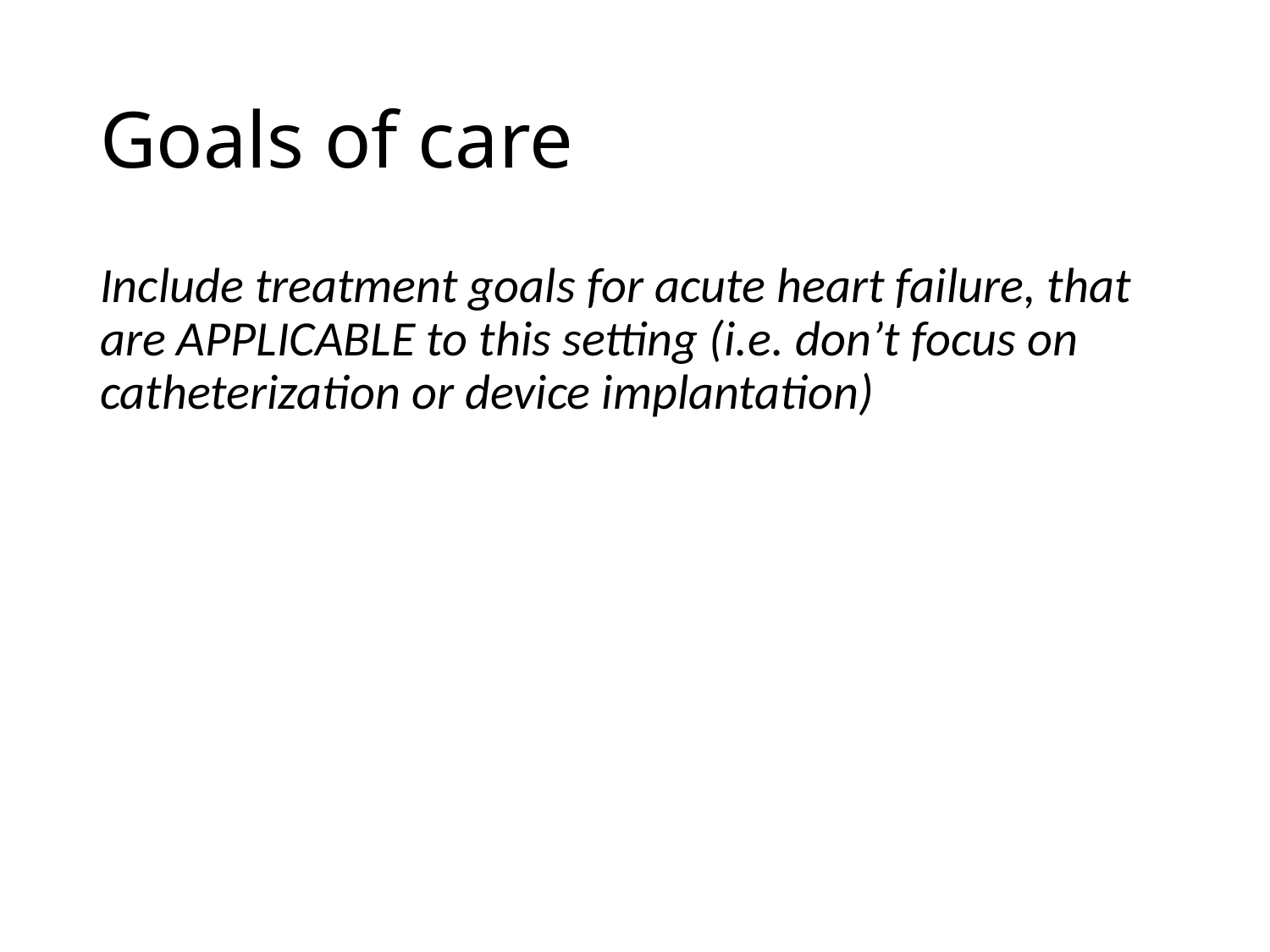

# Goals of care
Include treatment goals for acute heart failure, that are APPLICABLE to this setting (i.e. don’t focus on catheterization or device implantation)

## Slide 28
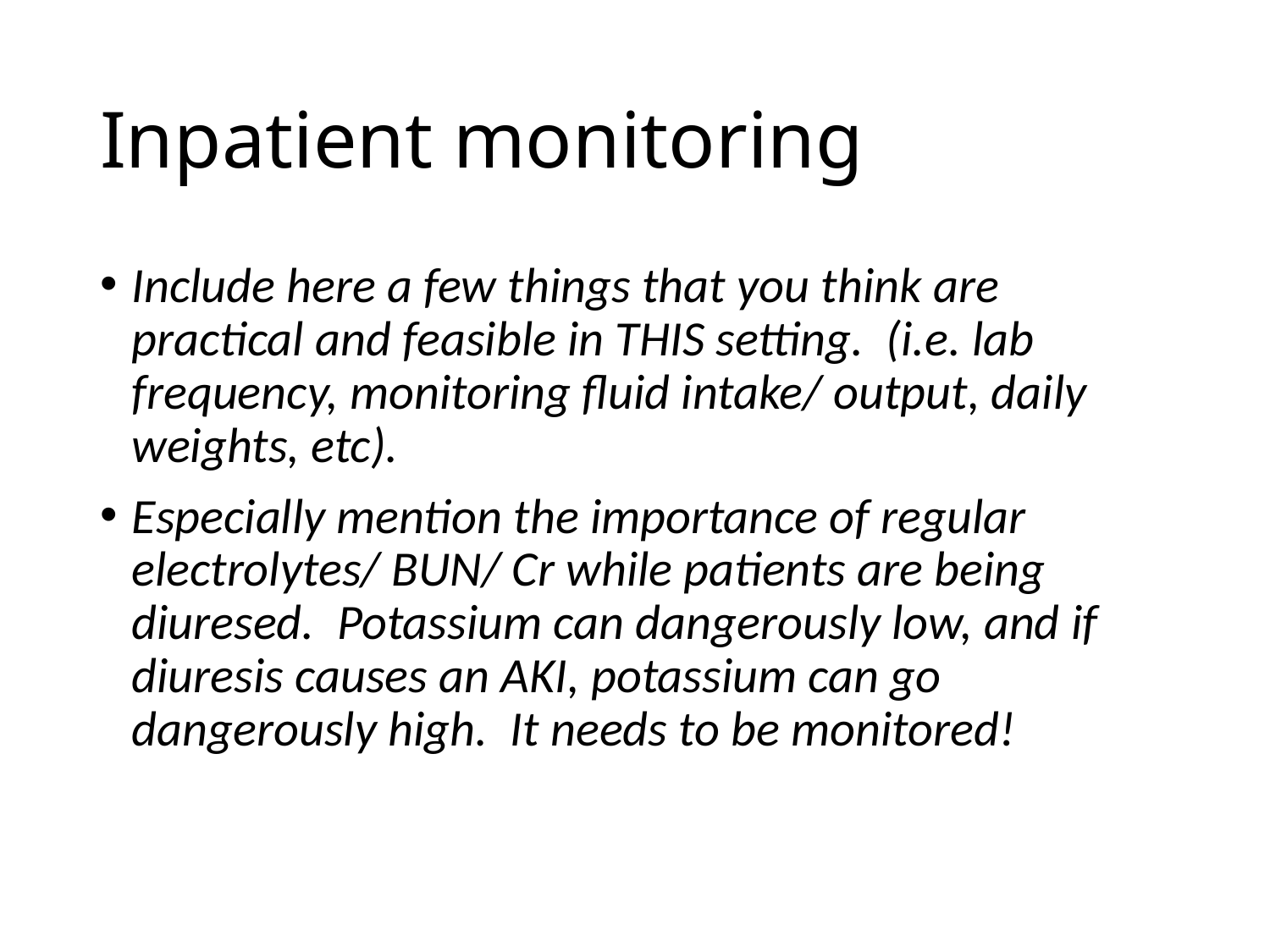

# Inpatient monitoring
Include here a few things that you think are practical and feasible in THIS setting. (i.e. lab frequency, monitoring fluid intake/ output, daily weights, etc).
Especially mention the importance of regular electrolytes/ BUN/ Cr while patients are being diuresed. Potassium can dangerously low, and if diuresis causes an AKI, potassium can go dangerously high. It needs to be monitored!

## Slide 29
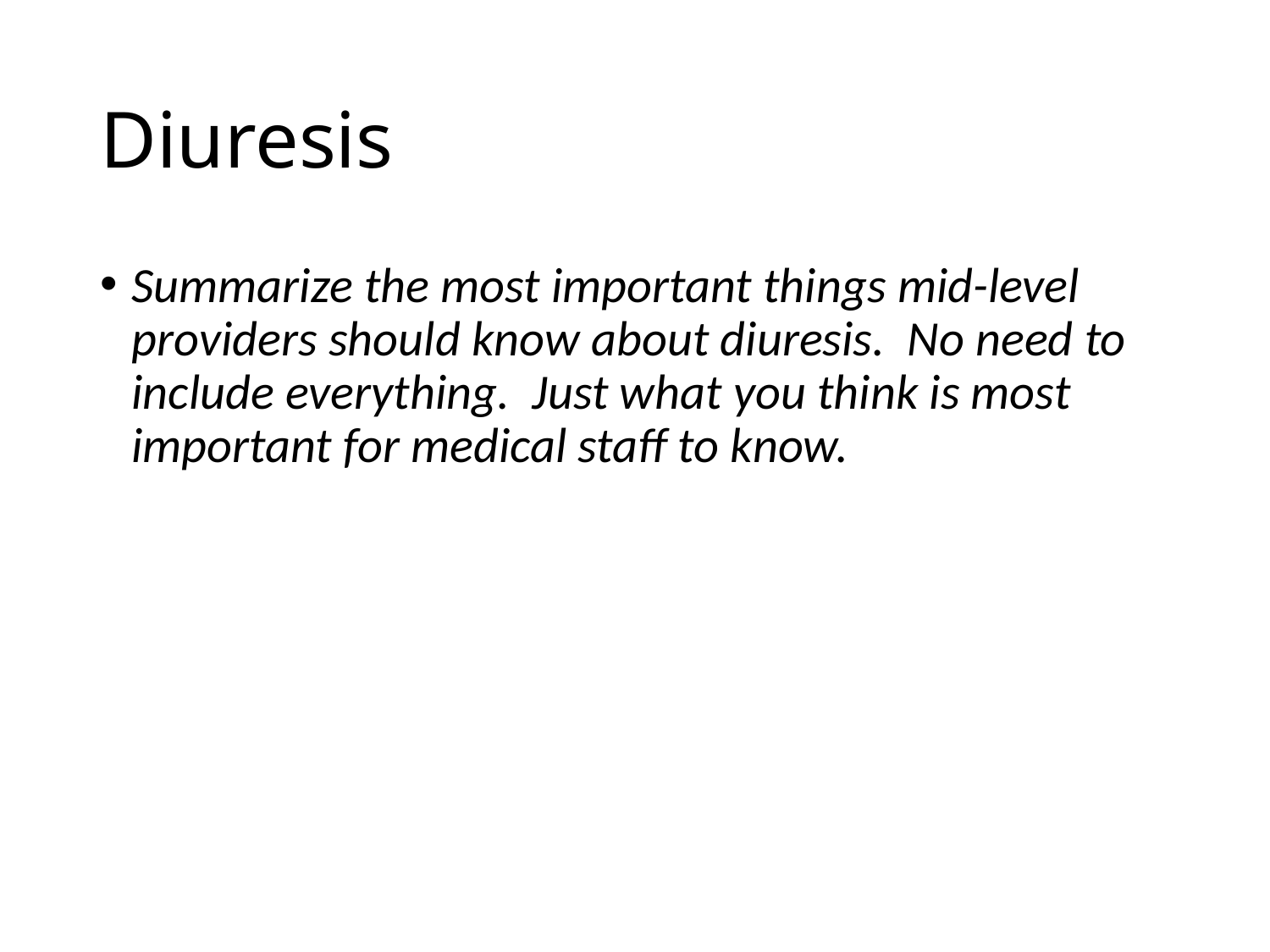

# Diuresis
Summarize the most important things mid-level providers should know about diuresis. No need to include everything. Just what you think is most important for medical staff to know.

## Slide 30
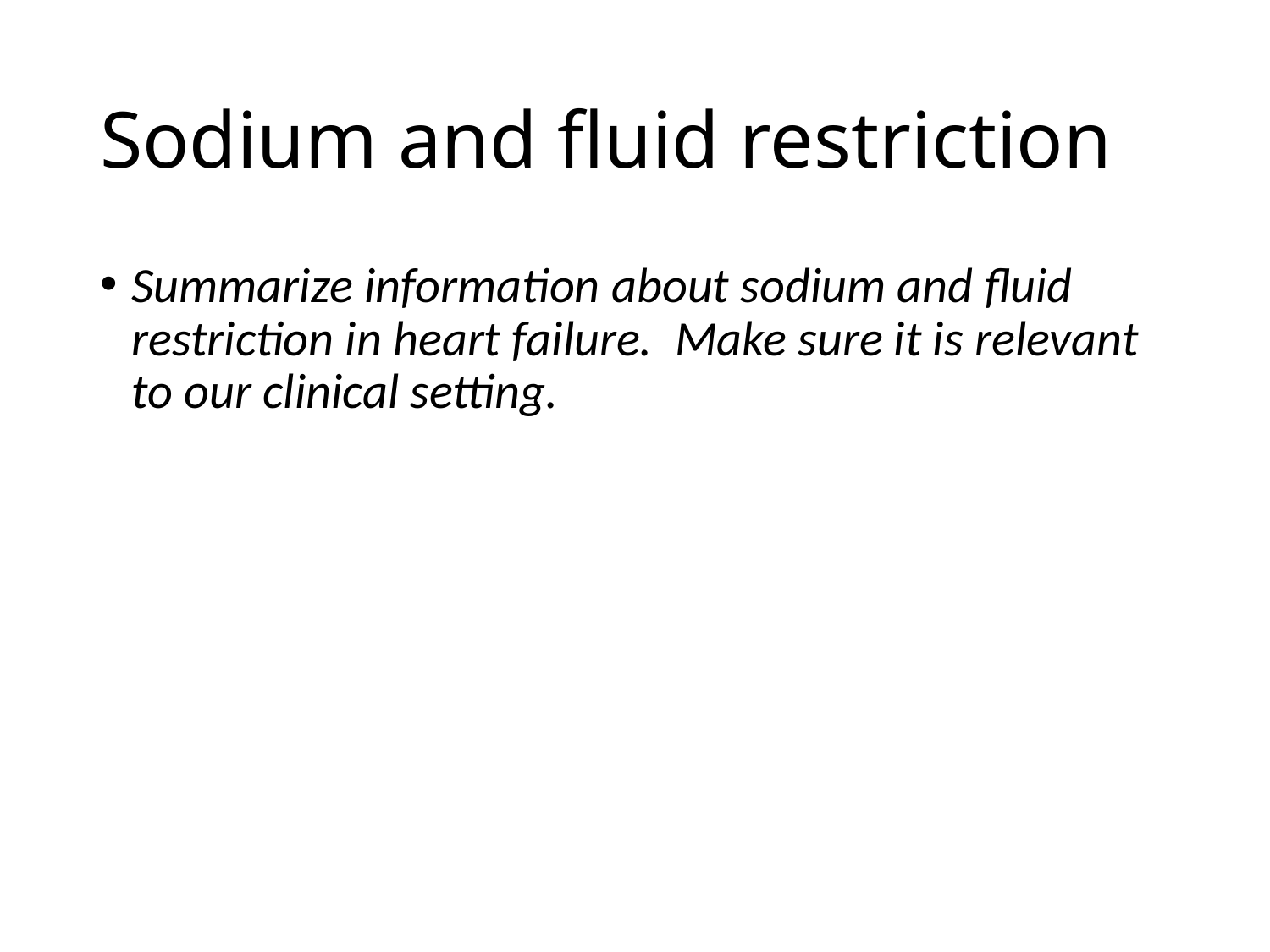

# Sodium and fluid restriction
Summarize information about sodium and fluid restriction in heart failure. Make sure it is relevant to our clinical setting.

## Slide 31
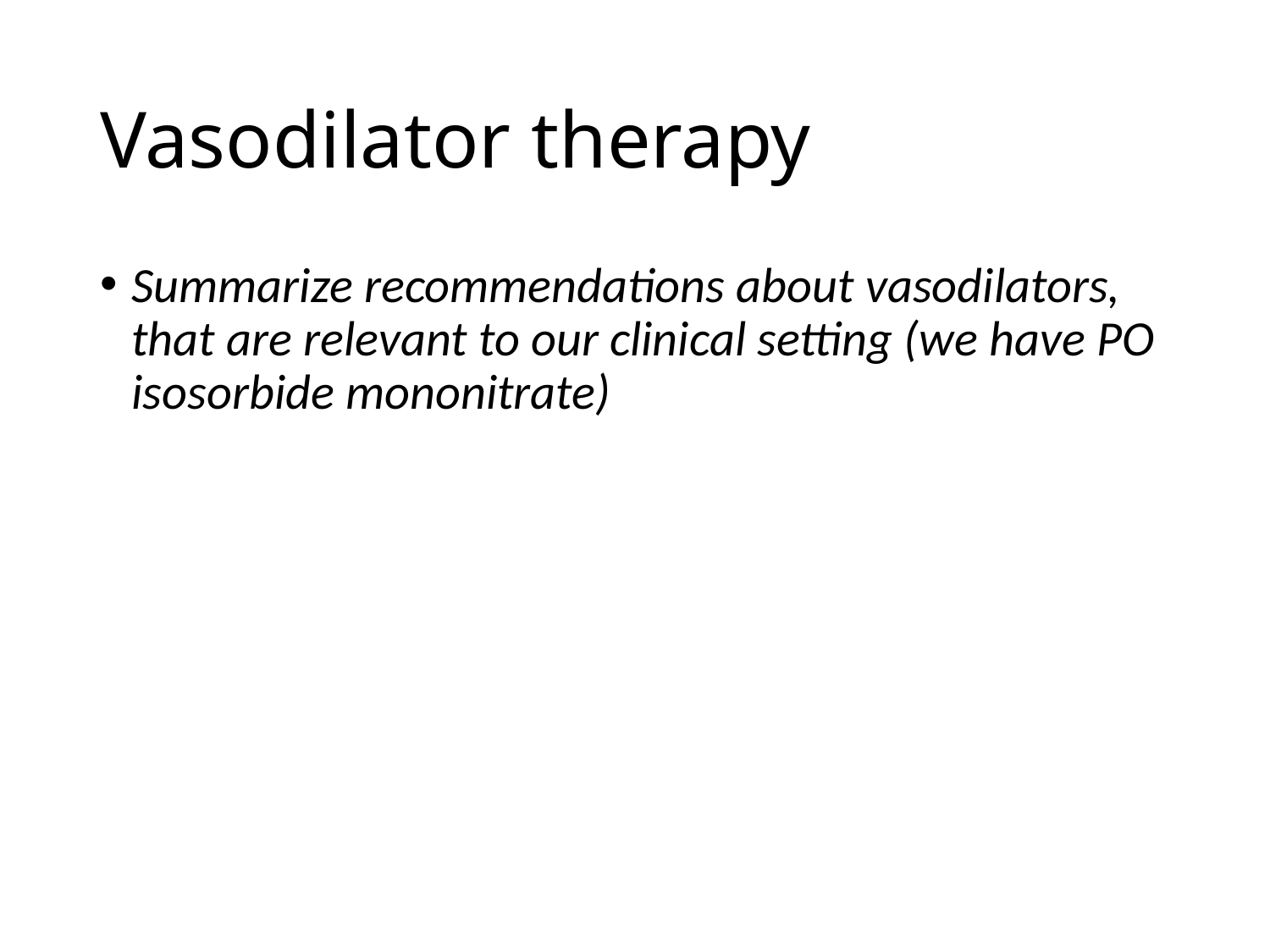

# Vasodilator therapy
Summarize recommendations about vasodilators, that are relevant to our clinical setting (we have PO isosorbide mononitrate)

## Slide 32
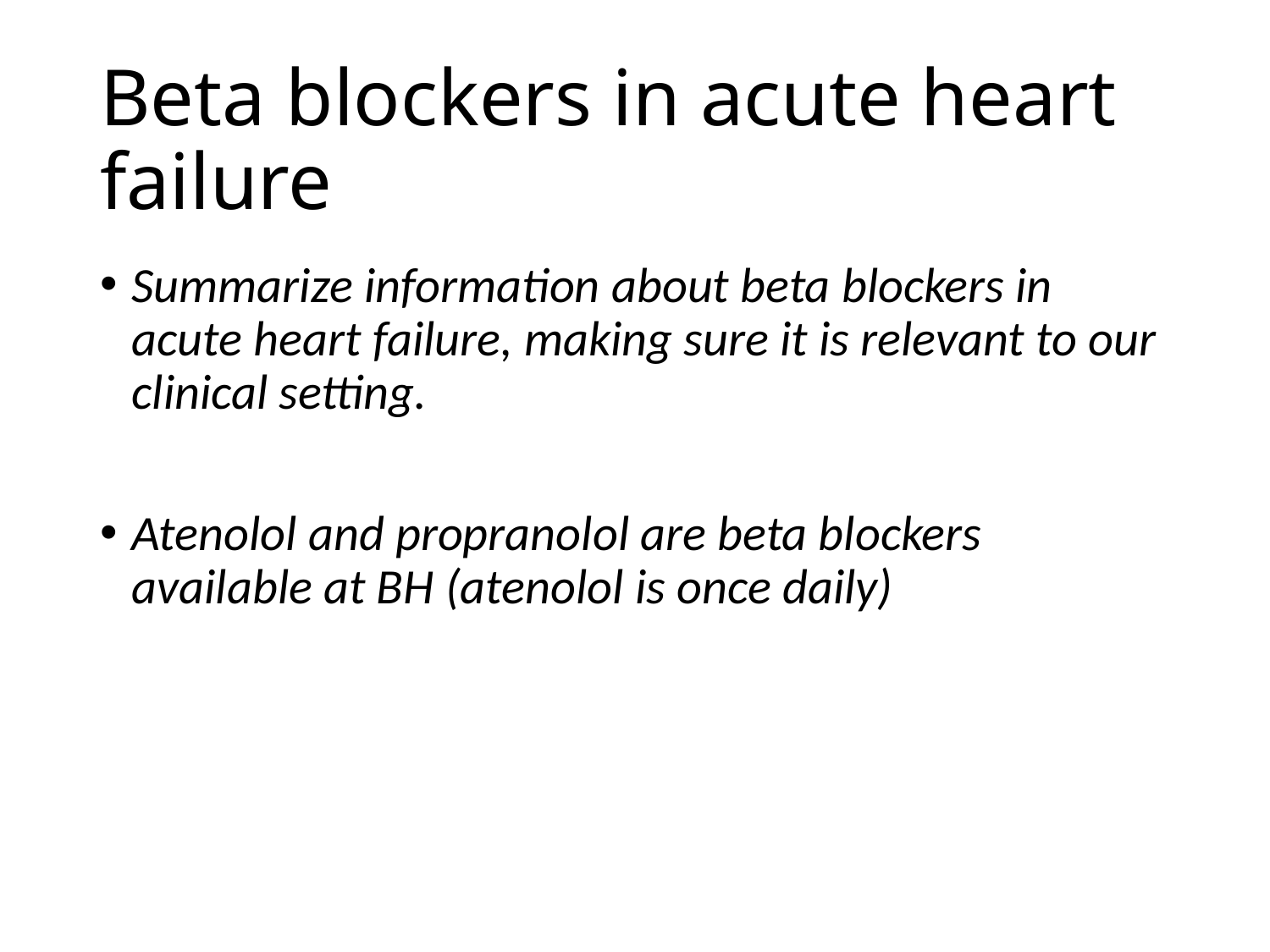

# Beta blockers in acute heart failure
Summarize information about beta blockers in acute heart failure, making sure it is relevant to our clinical setting.
Atenolol and propranolol are beta blockers available at BH (atenolol is once daily)

## Slide 33
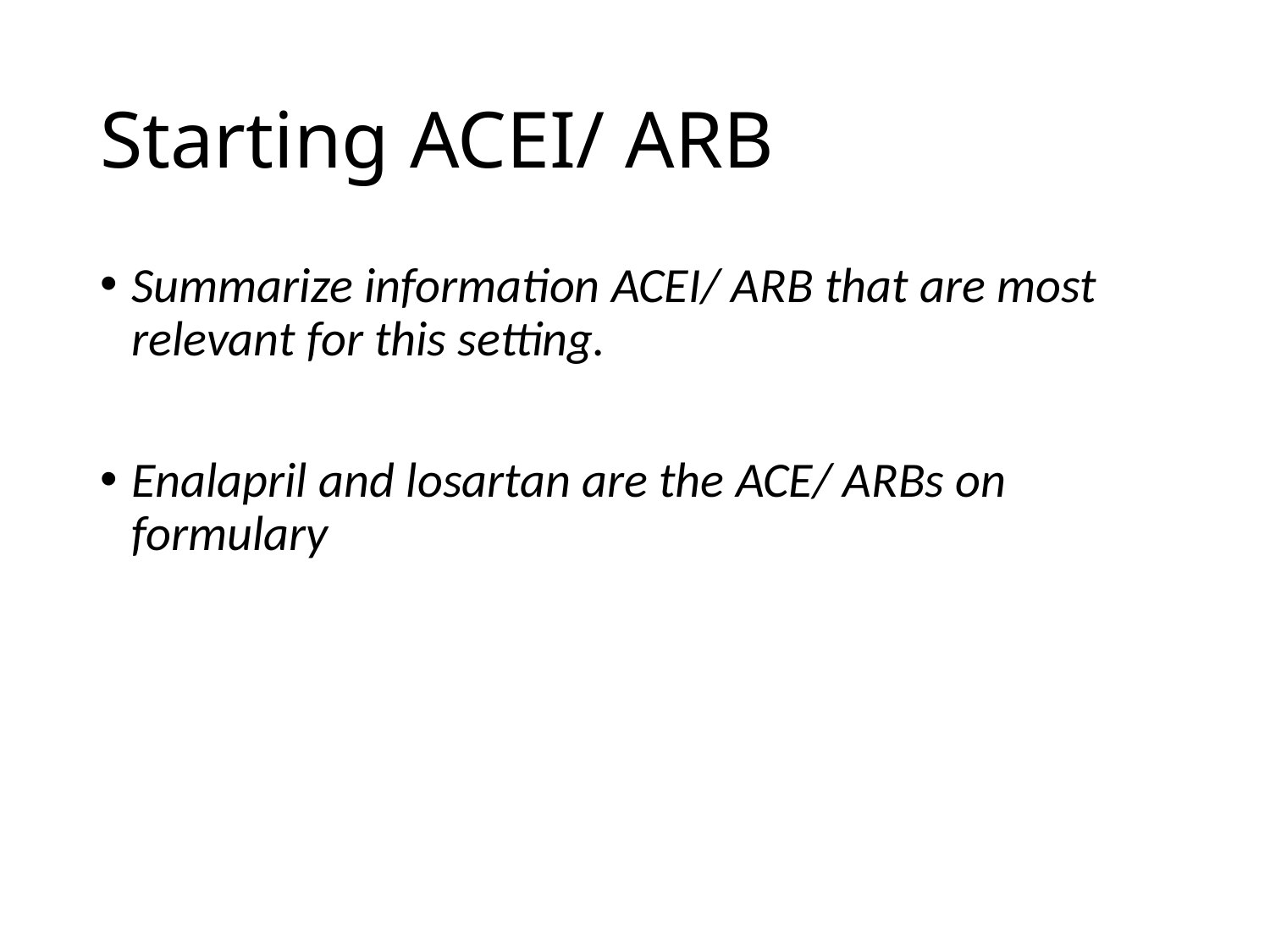

# Starting ACEI/ ARB
Summarize information ACEI/ ARB that are most relevant for this setting.
Enalapril and losartan are the ACE/ ARBs on formulary

## Slide 34
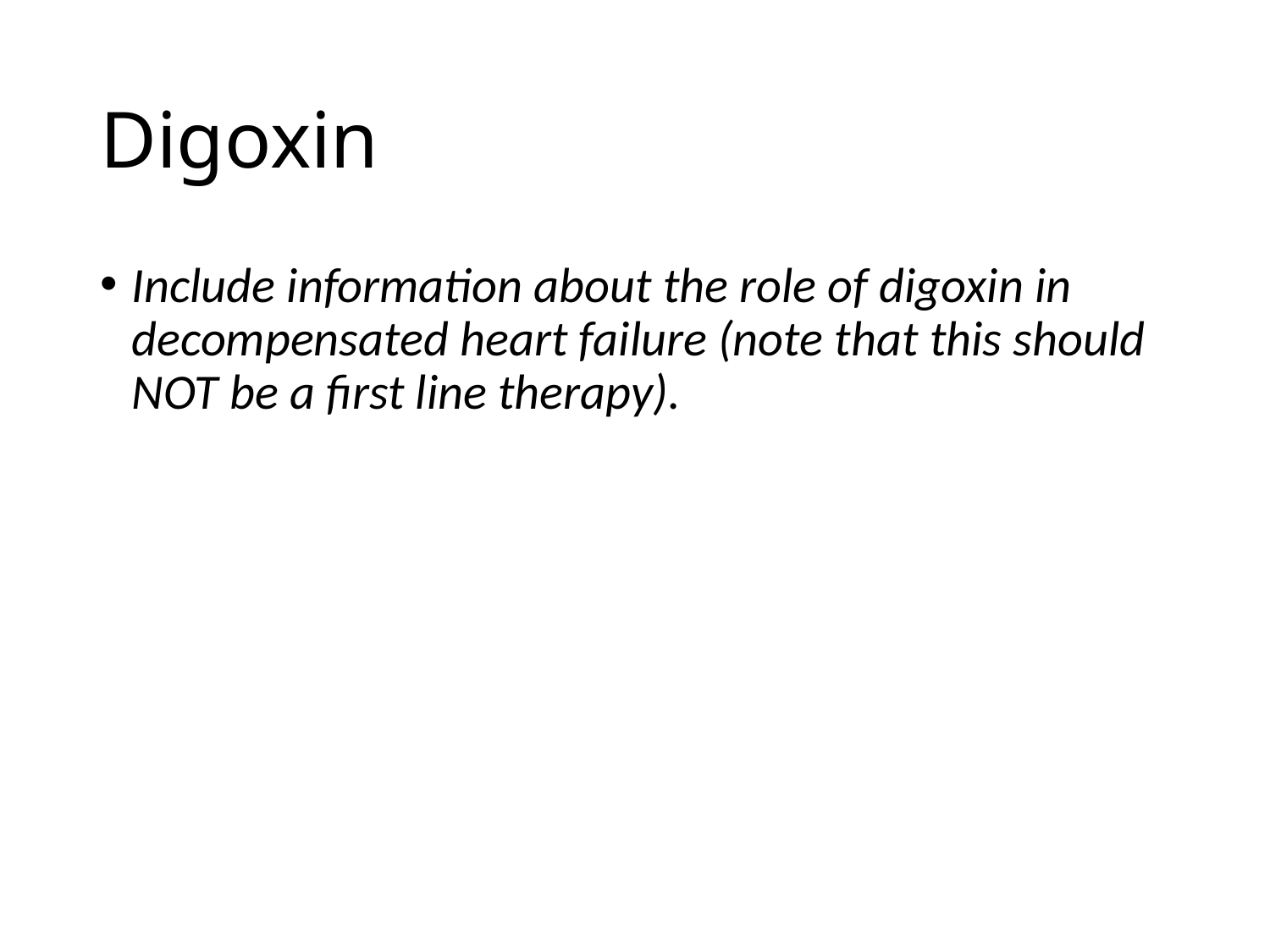

# Digoxin
Include information about the role of digoxin in decompensated heart failure (note that this should NOT be a first line therapy).

## Slide 35
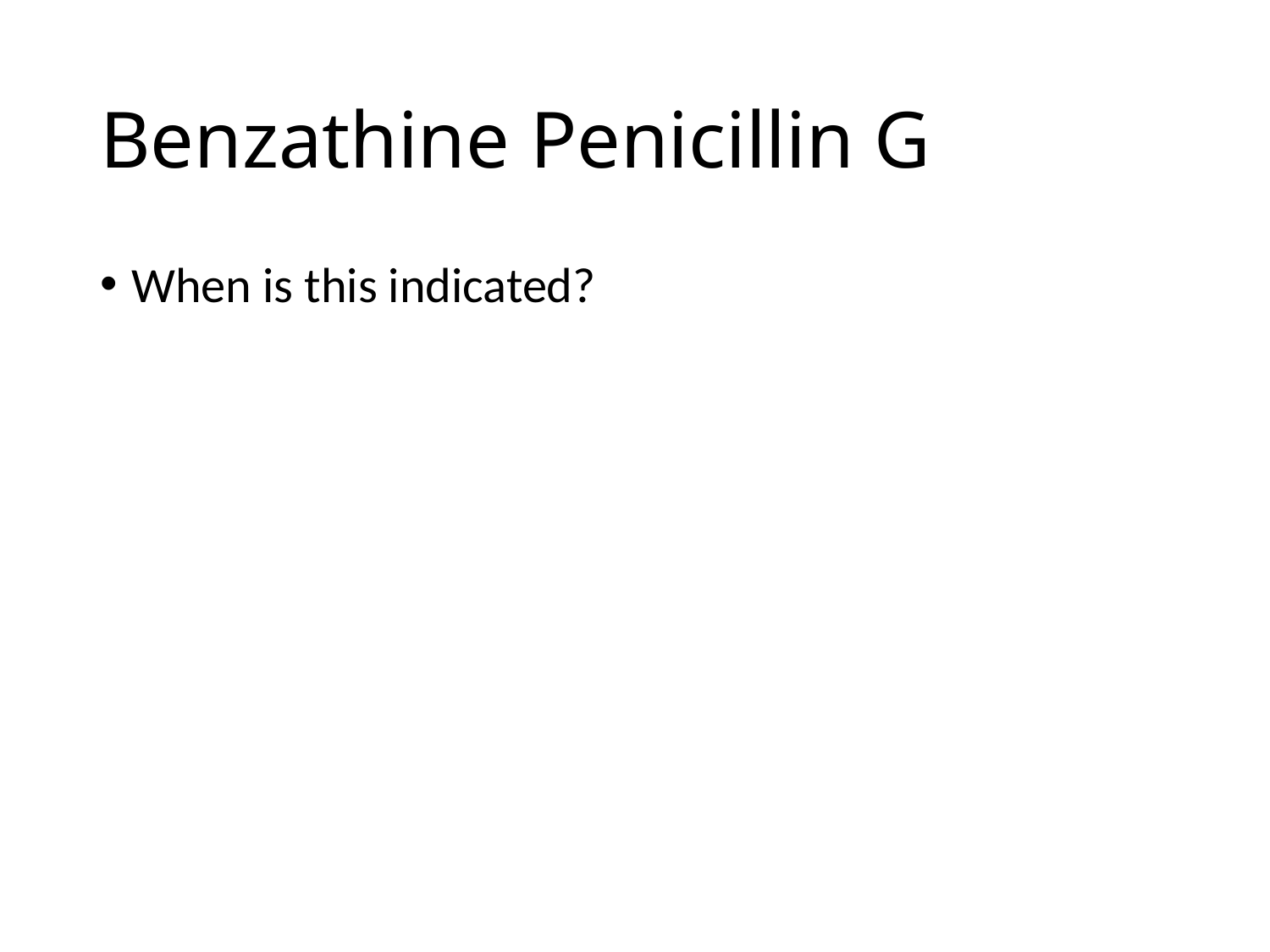

# Benzathine Penicillin G
When is this indicated?

## Slide 36
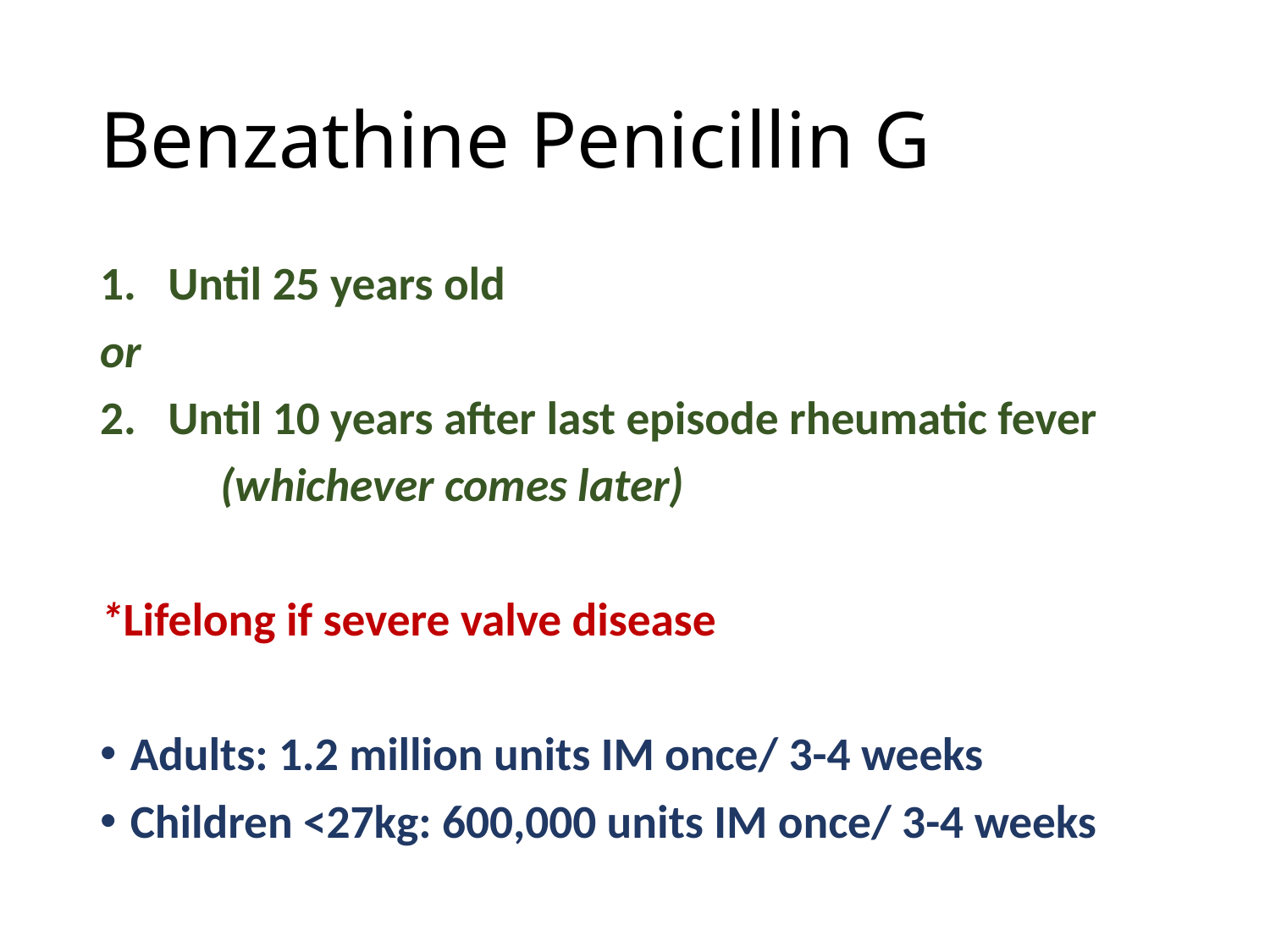

# Benzathine Penicillin G
Until 25 years old
or
2. Until 10 years after last episode rheumatic fever
 	(whichever comes later)
*Lifelong if severe valve disease
Adults: 1.2 million units IM once/ 3-4 weeks
Children <27kg: 600,000 units IM once/ 3-4 weeks

## Slide 37
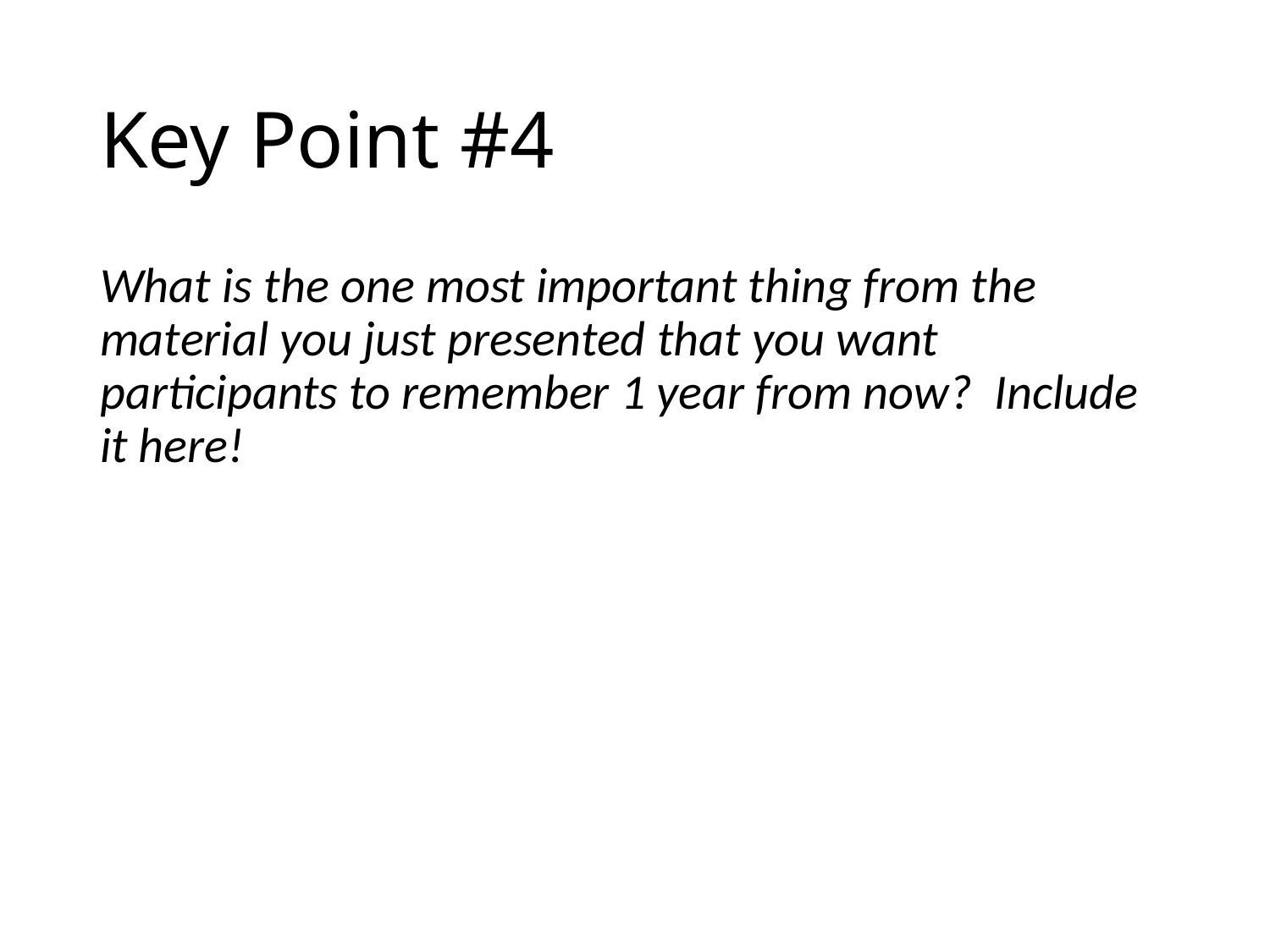

# Key Point #4
What is the one most important thing from the material you just presented that you want participants to remember 1 year from now? Include it here!

## Slide 38
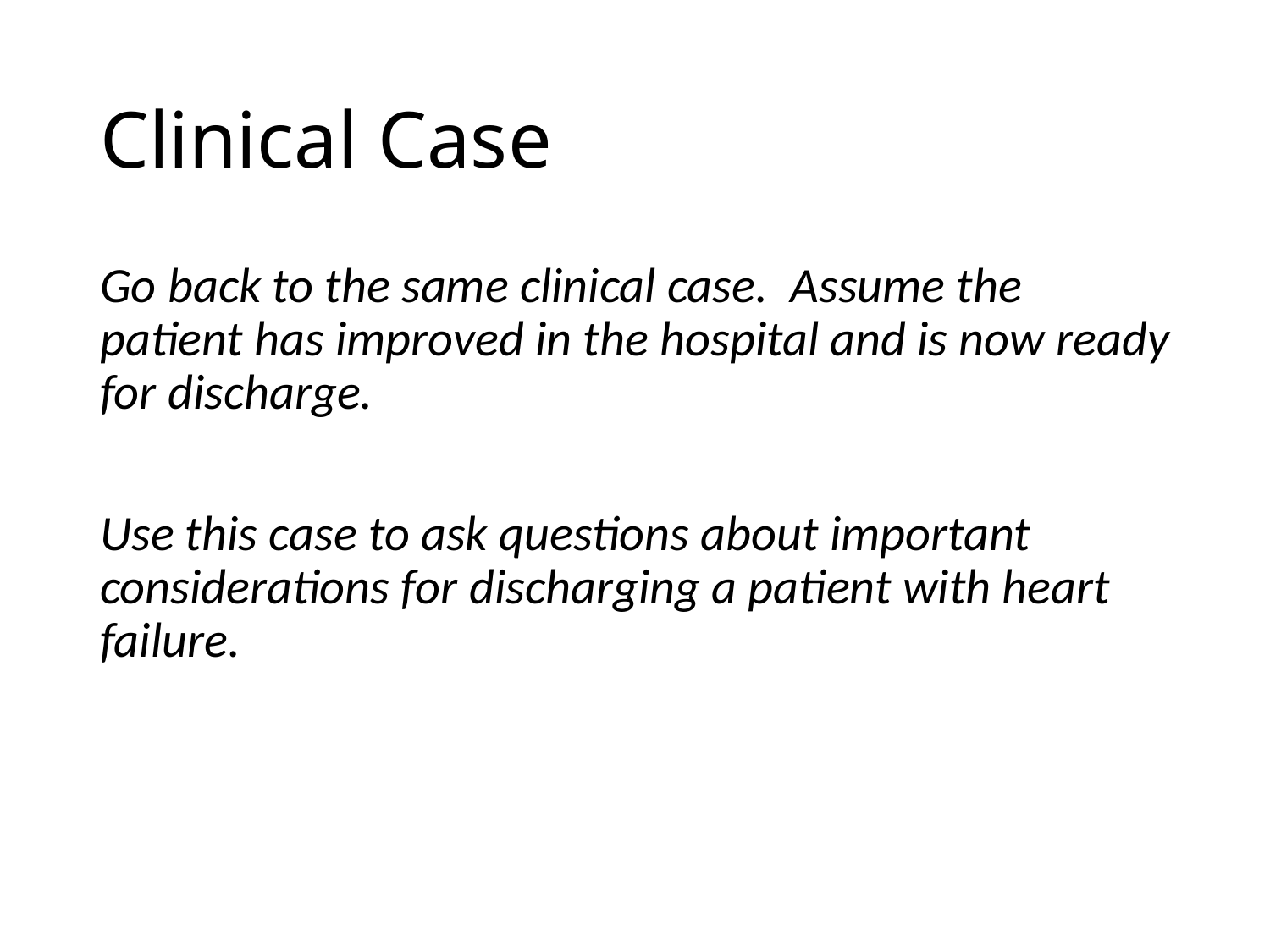

# Clinical Case
Go back to the same clinical case. Assume the patient has improved in the hospital and is now ready for discharge.
Use this case to ask questions about important considerations for discharging a patient with heart failure.

## Slide 39
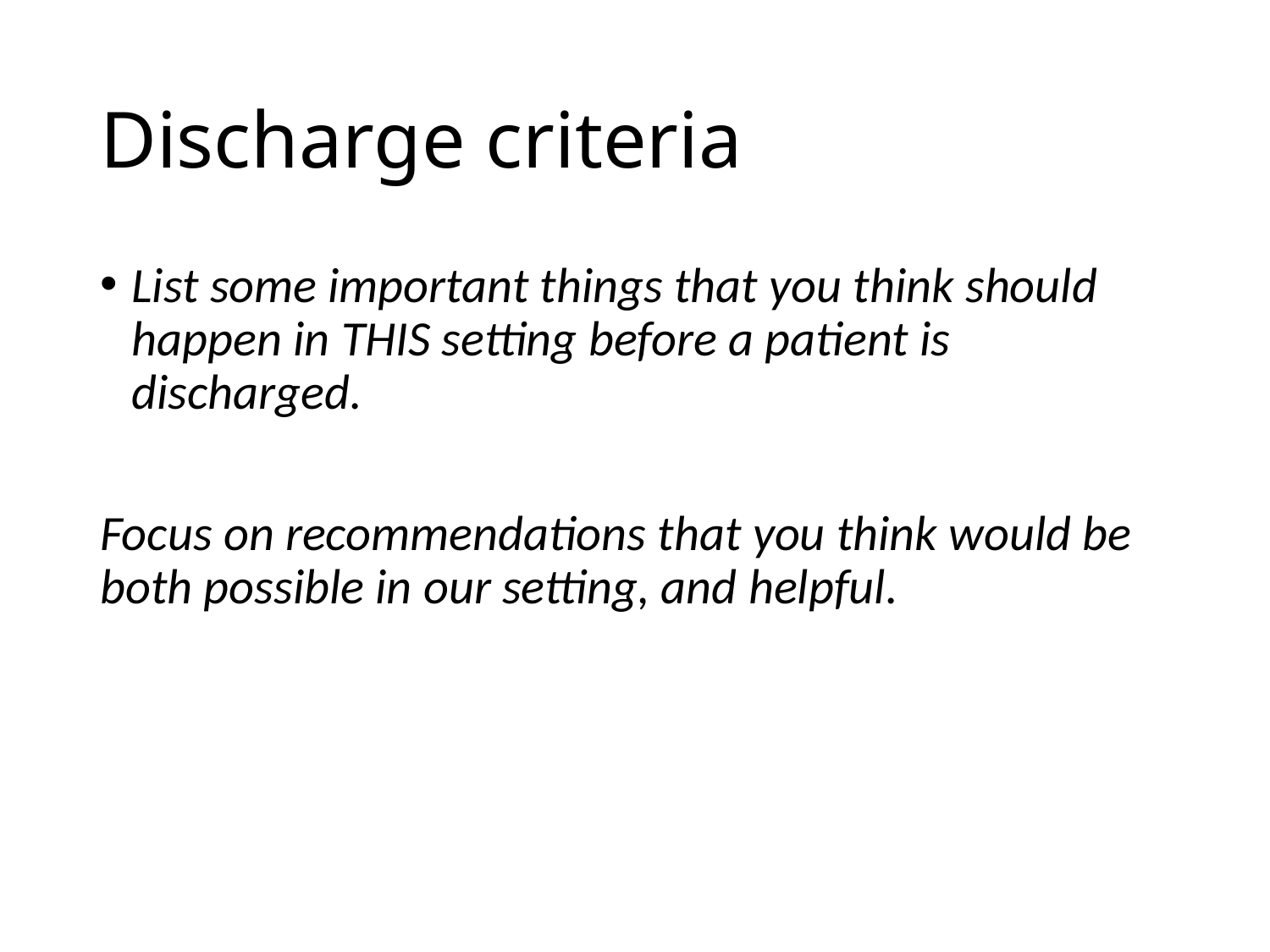

# Discharge criteria
List some important things that you think should happen in THIS setting before a patient is discharged.
Focus on recommendations that you think would be both possible in our setting, and helpful.

## Slide 40
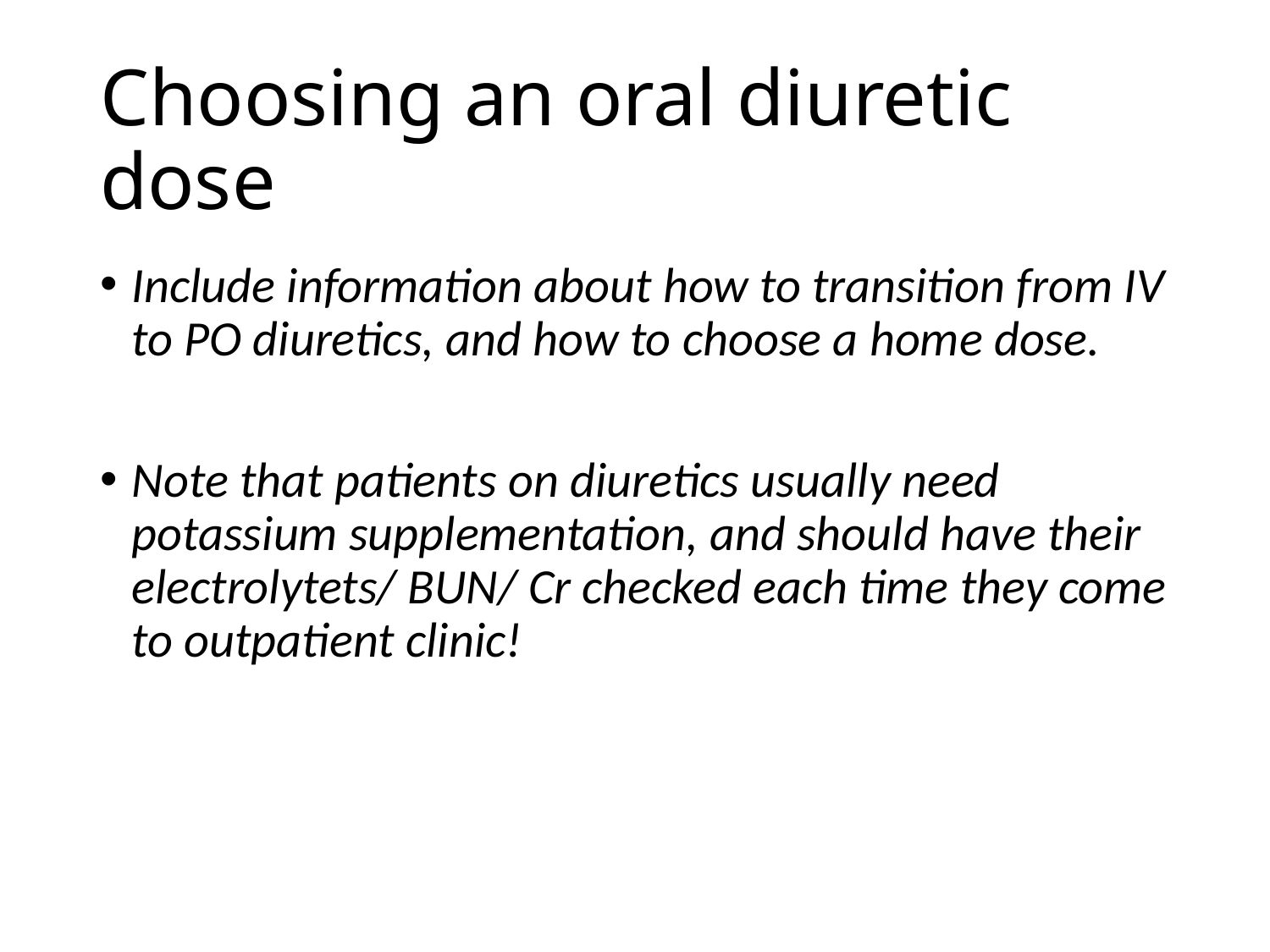

# Choosing an oral diuretic dose
Include information about how to transition from IV to PO diuretics, and how to choose a home dose.
Note that patients on diuretics usually need potassium supplementation, and should have their electrolytets/ BUN/ Cr checked each time they come to outpatient clinic!

## Slide 41
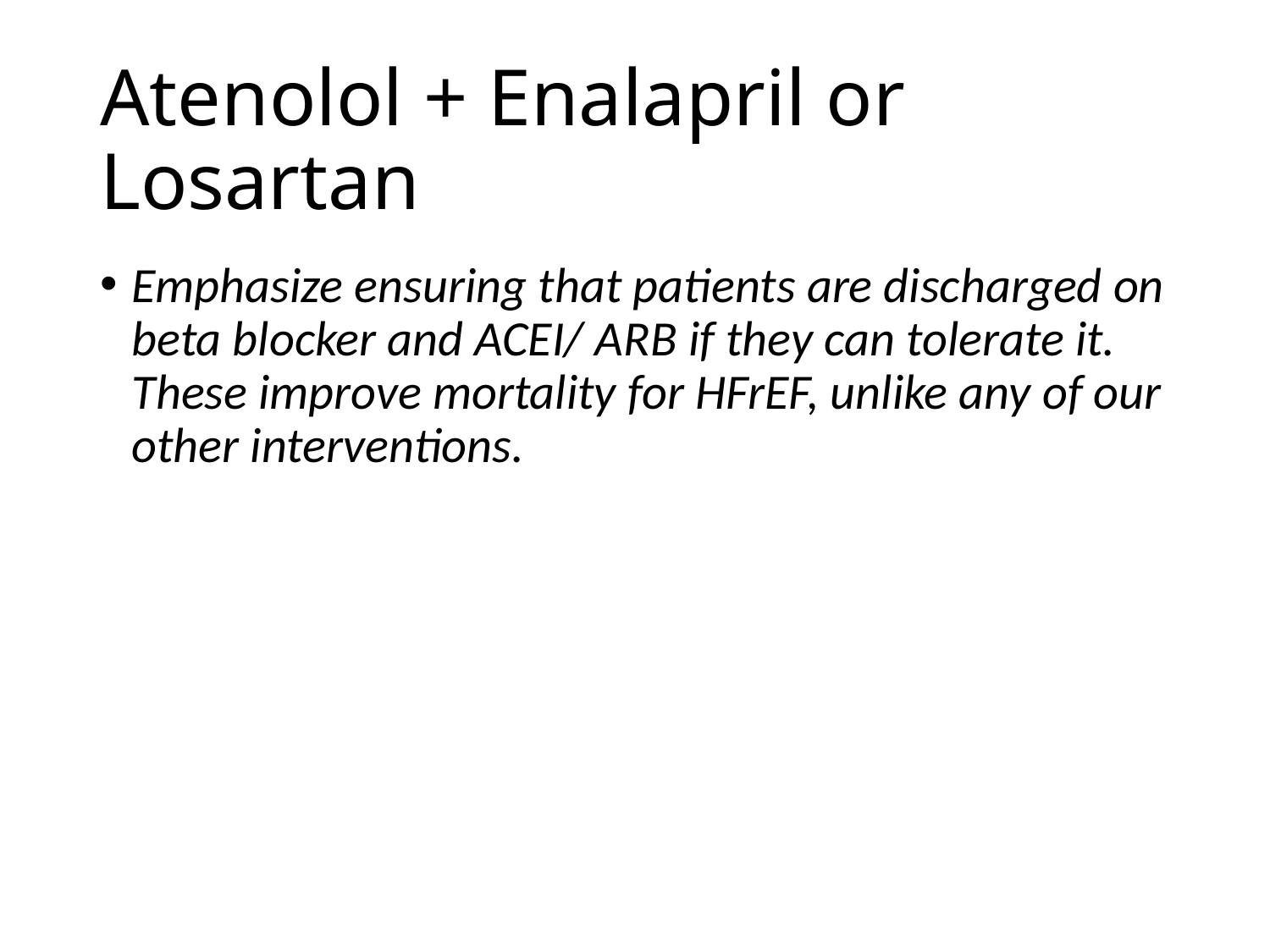

# Atenolol + Enalapril or Losartan
Emphasize ensuring that patients are discharged on beta blocker and ACEI/ ARB if they can tolerate it. These improve mortality for HFrEF, unlike any of our other interventions.

## Slide 42
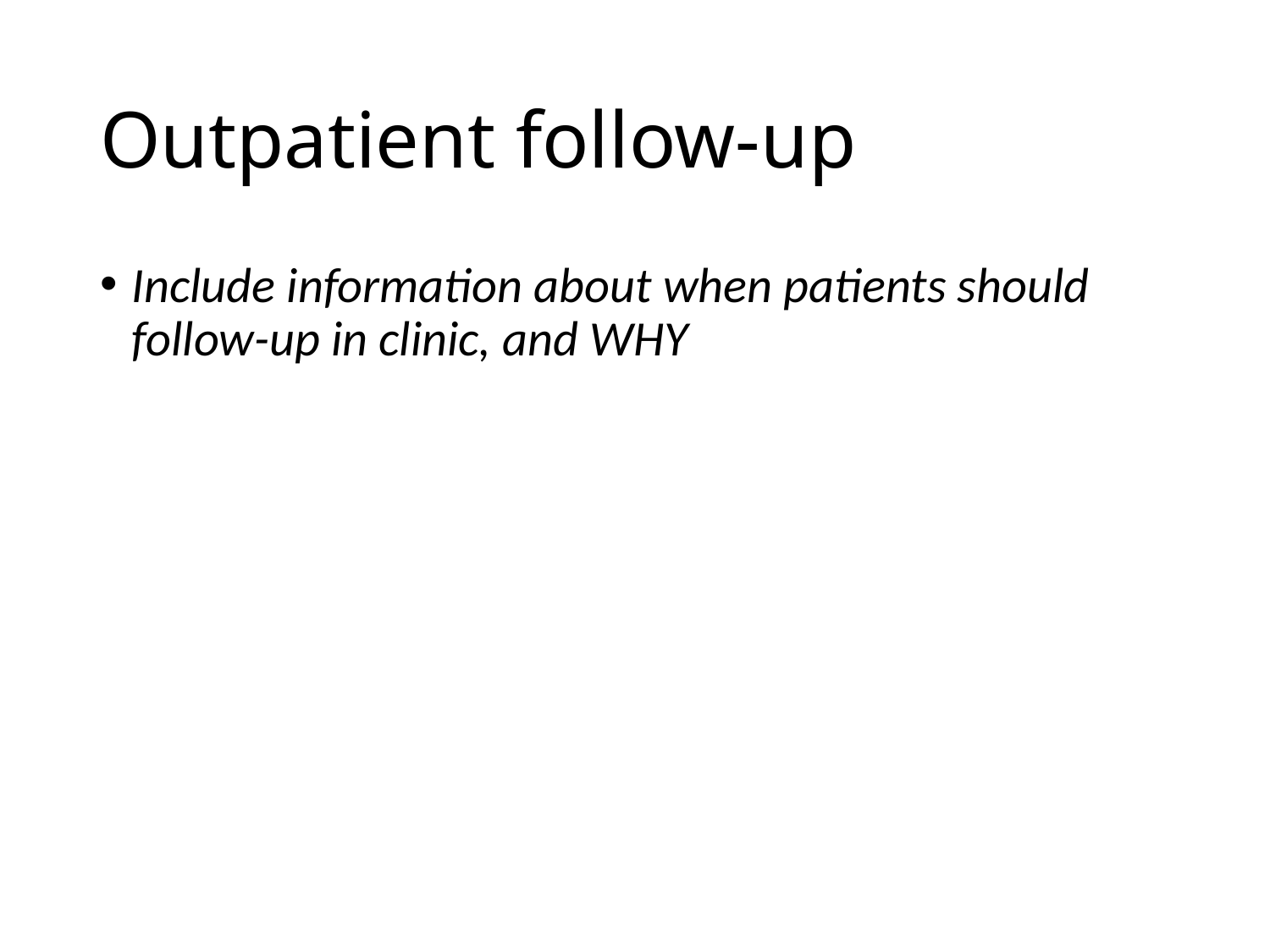

# Outpatient follow-up
Include information about when patients should follow-up in clinic, and WHY

## Slide 43
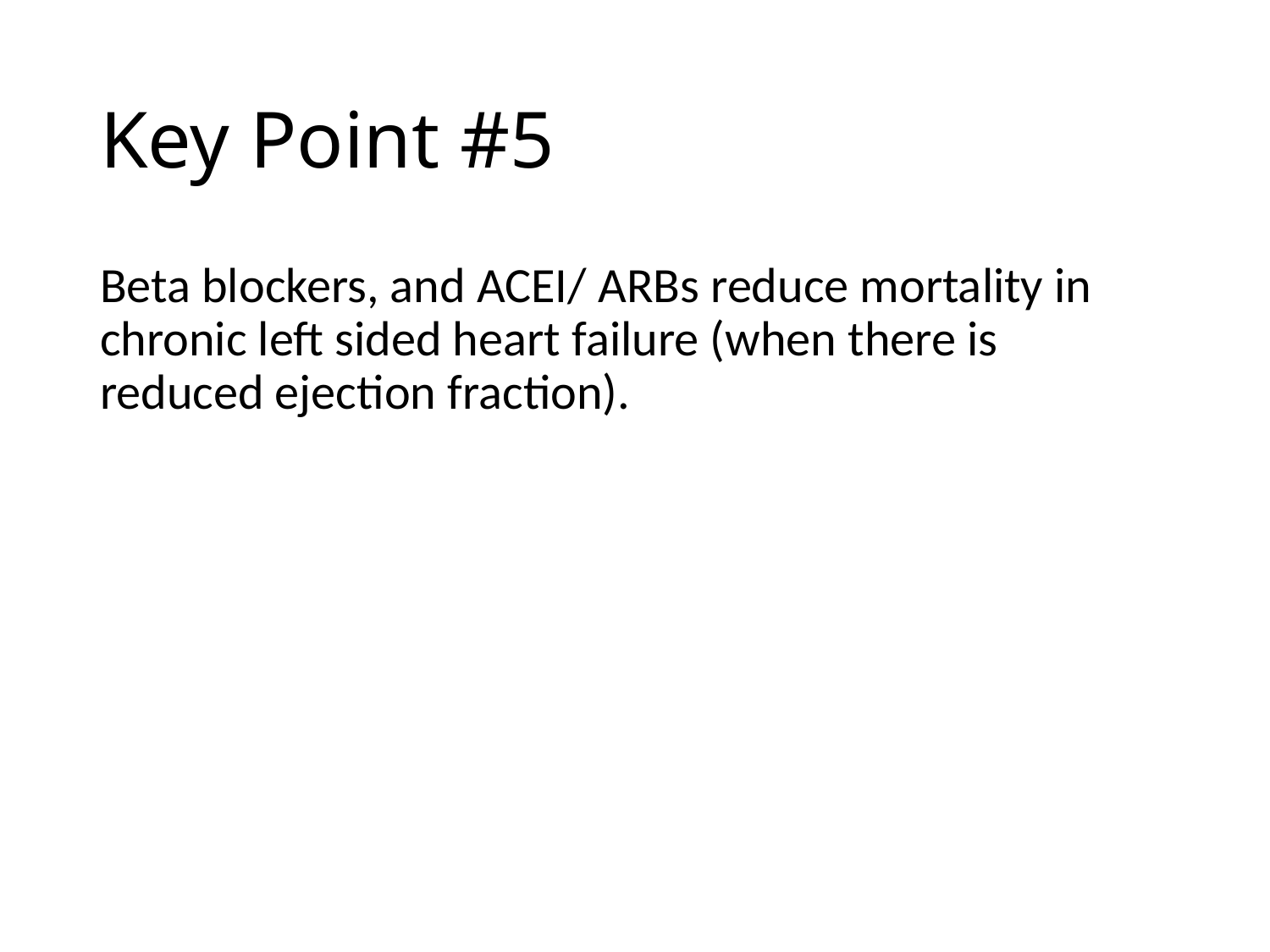

# Key Point #5
Beta blockers, and ACEI/ ARBs reduce mortality in chronic left sided heart failure (when there is reduced ejection fraction).

## Slide 44
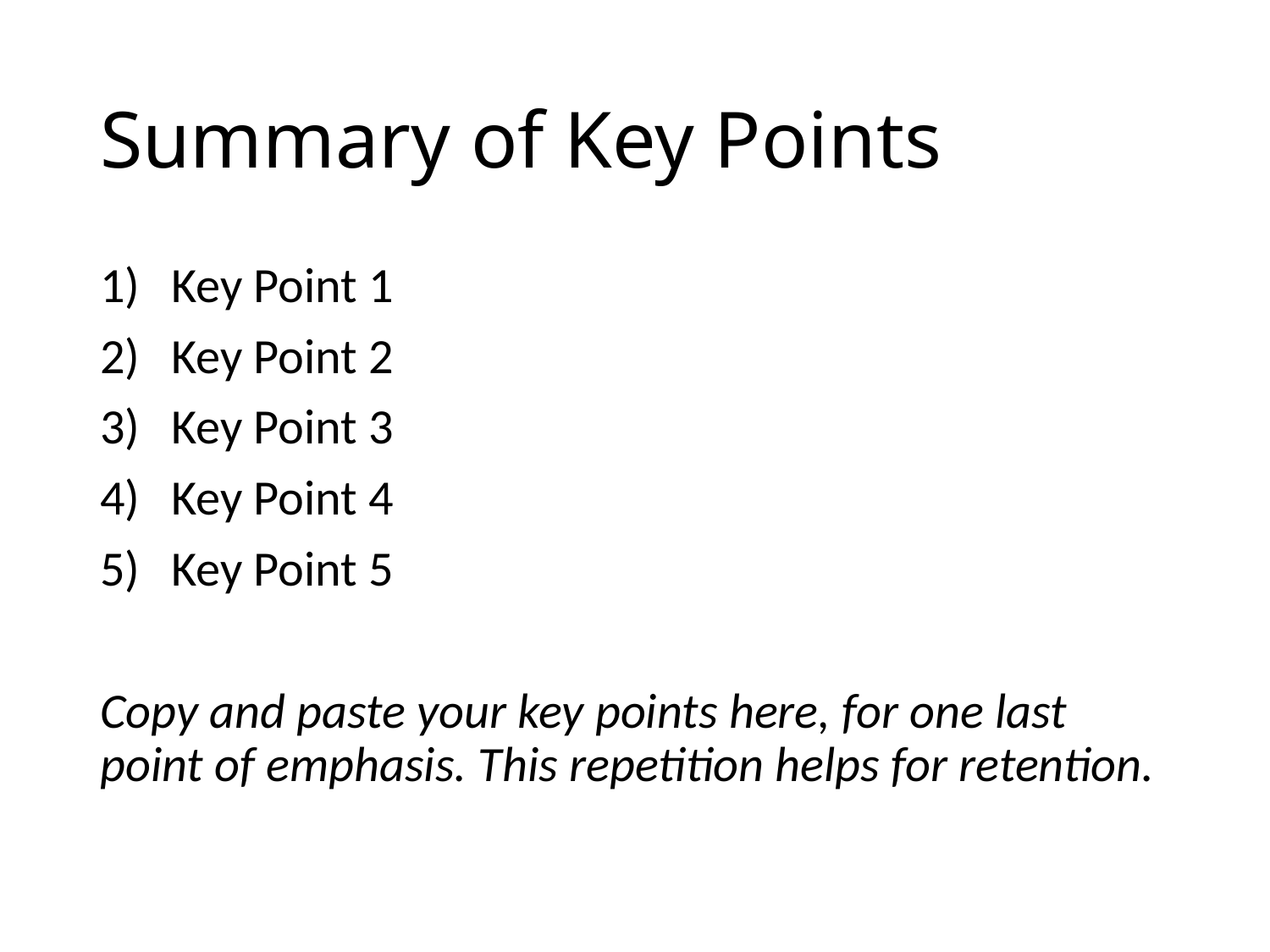

# Summary of Key Points
Key Point 1
Key Point 2
Key Point 3
Key Point 4
Key Point 5
Copy and paste your key points here, for one last point of emphasis. This repetition helps for retention.

## Slide 45
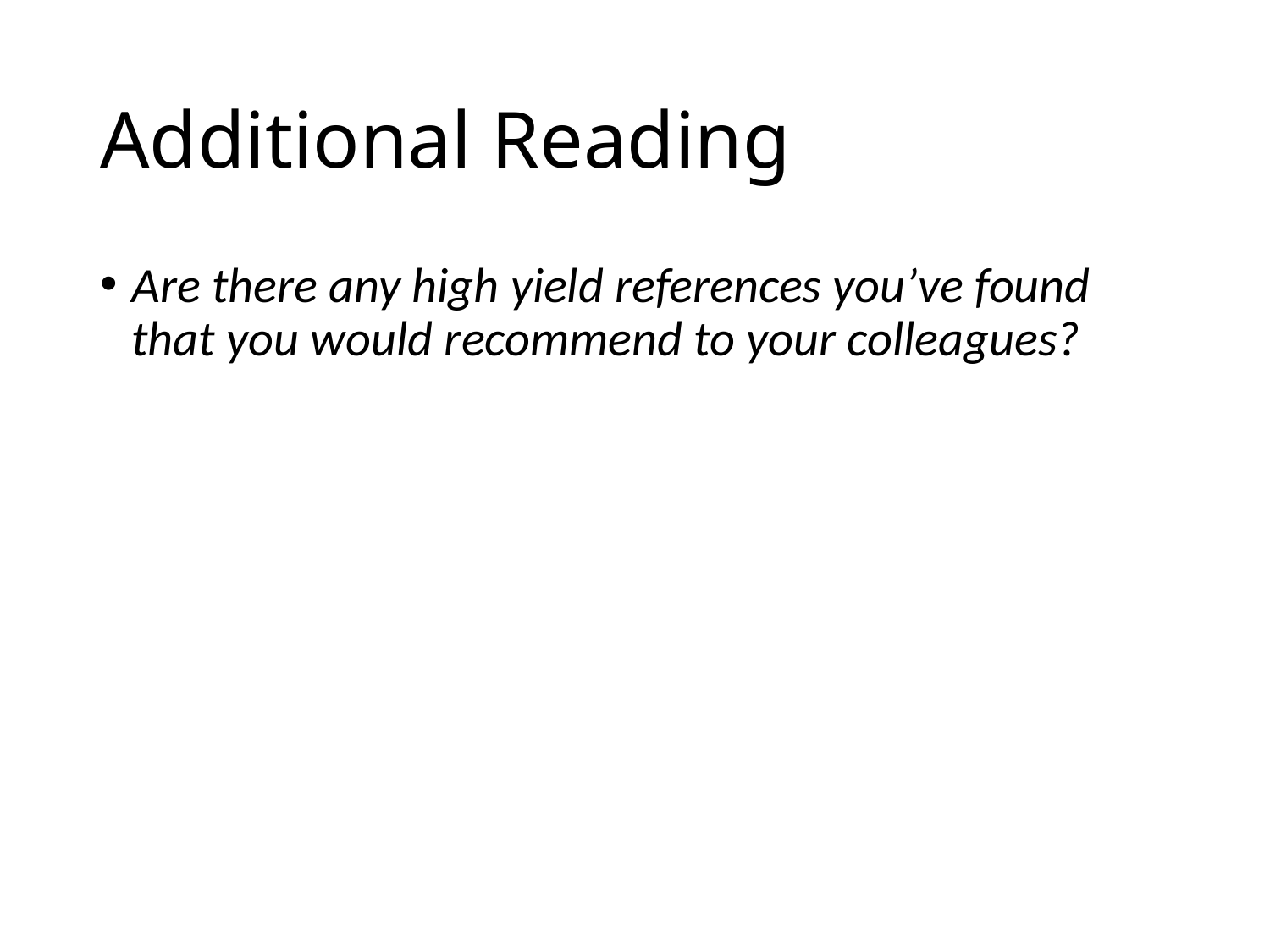

# Additional Reading
Are there any high yield references you’ve found that you would recommend to your colleagues?

## Slide 46
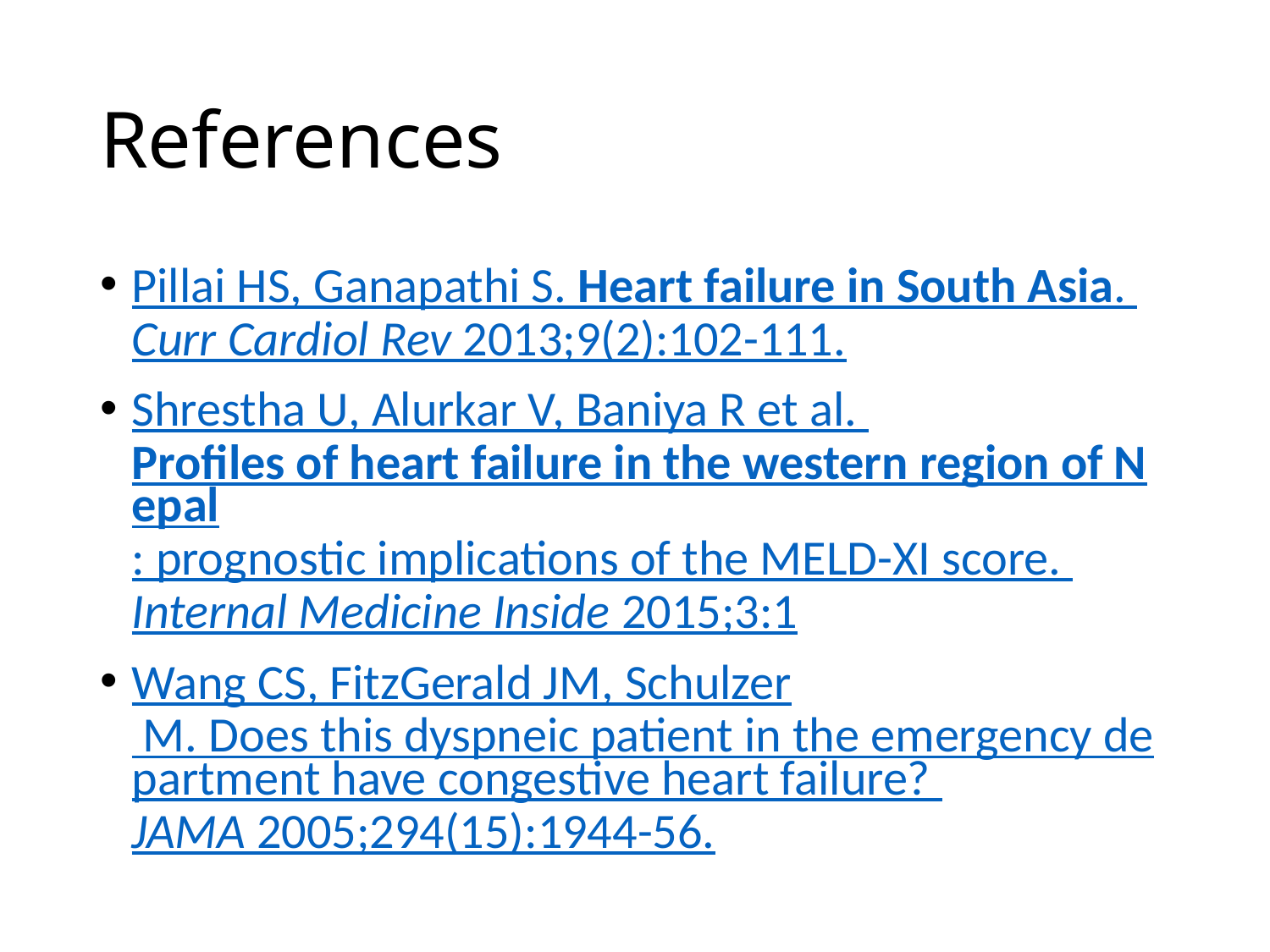

# References
Pillai HS, Ganapathi S. Heart failure in South Asia. Curr Cardiol Rev 2013;9(2):102-111.
Shrestha U, Alurkar V, Baniya R et al. Profiles of heart failure in the western region of Nepal: prognostic implications of the MELD-XI score. Internal Medicine Inside 2015;3:1
Wang CS, FitzGerald JM, Schulzer M. Does this dyspneic patient in the emergency department have congestive heart failure? JAMA 2005;294(15):1944-56.

## Slide 47
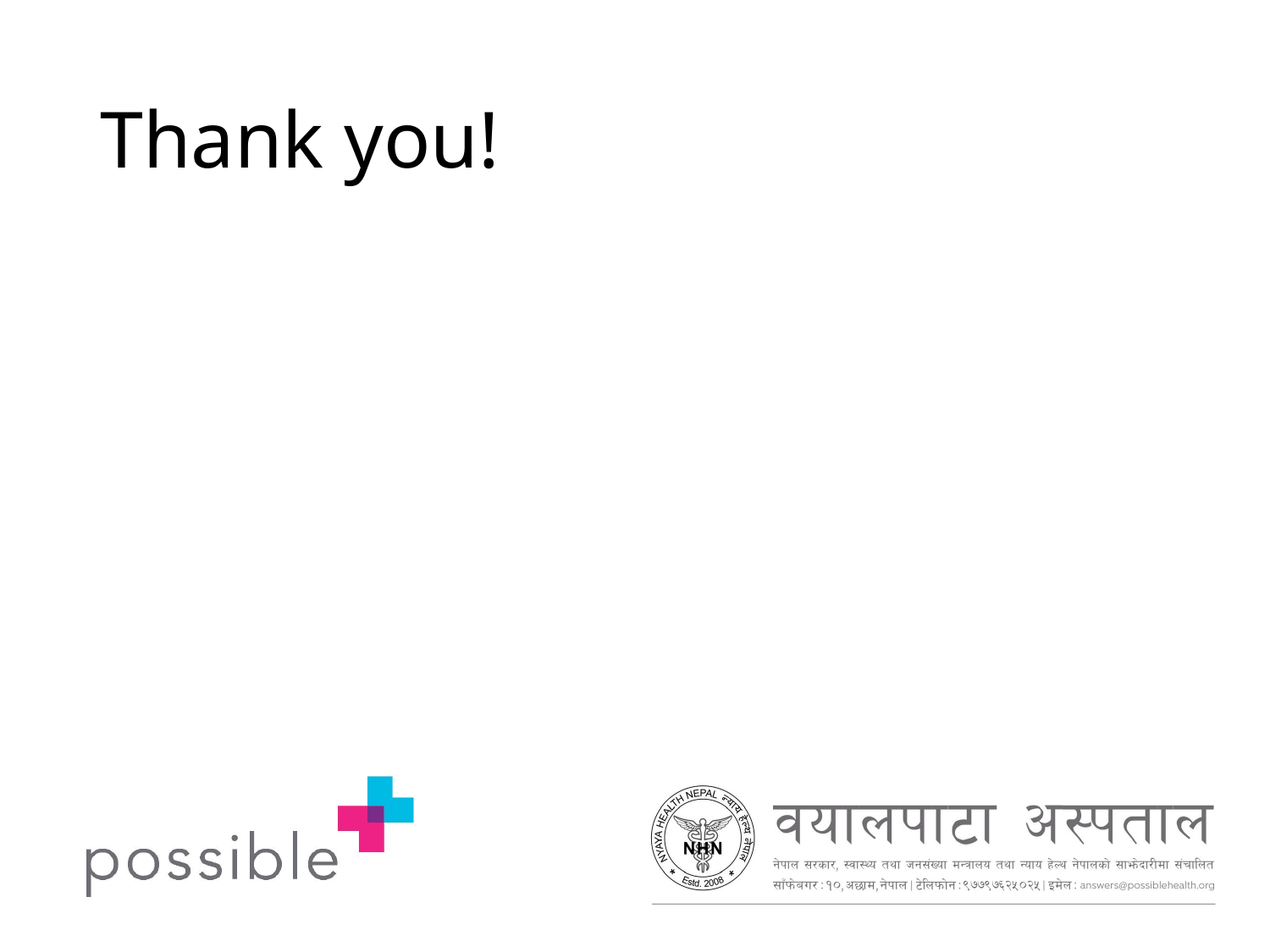

# Thank you!

## Slide 48
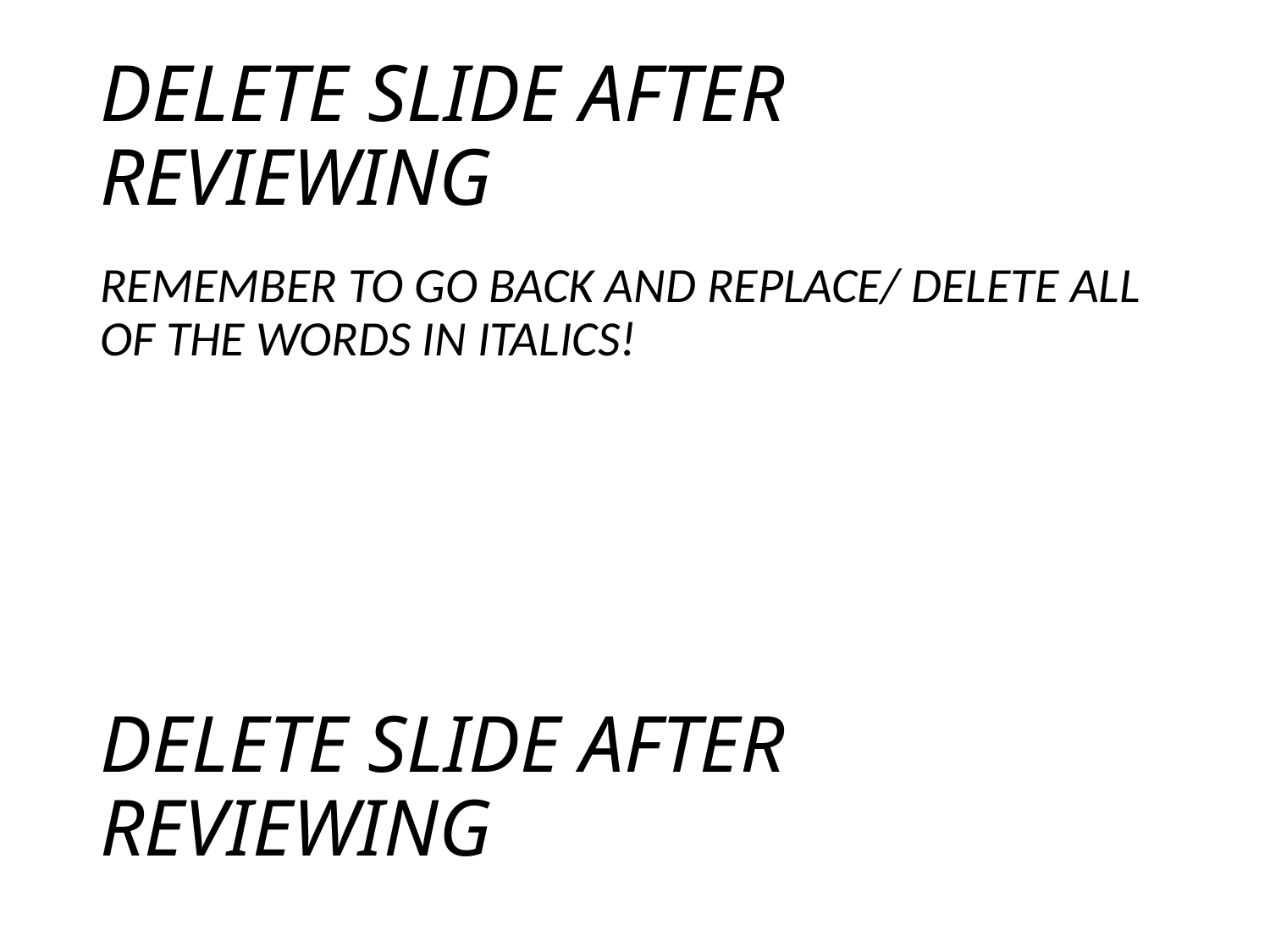

# DELETE SLIDE AFTER REVIEWING
REMEMBER TO GO BACK AND REPLACE/ DELETE ALL OF THE WORDS IN ITALICS!
DELETE SLIDE AFTER REVIEWING
