# Supplementary figures and images for: Transition to active learning in rural Nepal: an adaptable and scalable curriculum development model
Source: BMC Med Educ. 2019 Feb 20;19:61. doi: 10.1186/s12909-019-1492-3 (PMC6383231; doi:10.1186/s12909-019-1492-3)

# CME Curriculum Improvement: Theory of Change Map

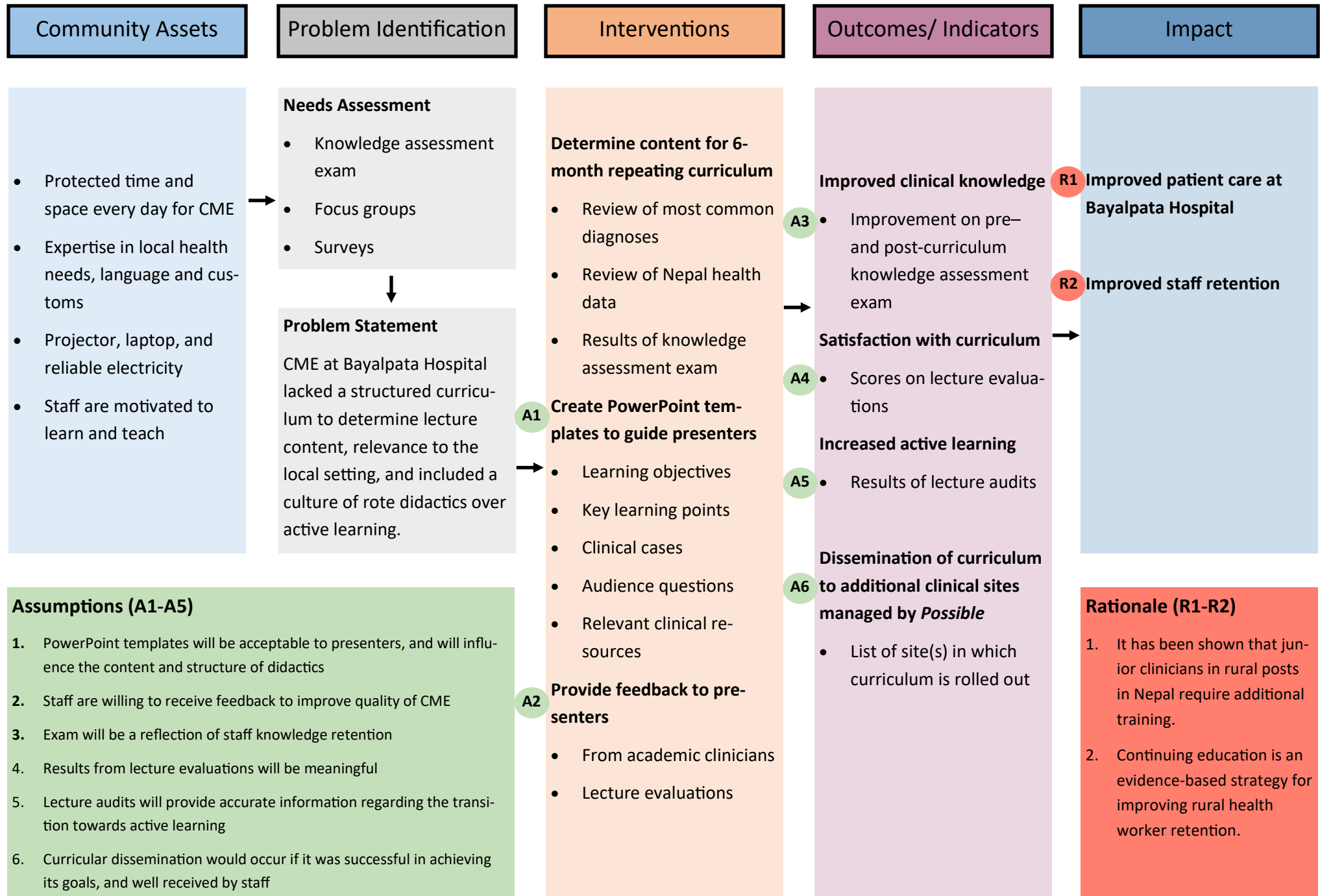

Supplement: Supplementary file 4 — Theory of Change map for curriculum improvement. (PDF 171 kb) [file 12909_2019_1492_MOESM4_ESM.pdf]
