# Supplementary material for: Transition to active learning in rural Nepal: an adaptable and scalable curriculum development model
Source: BMC Med Educ. 2019 Feb 20;19:61. doi: 10.1186/s12909-019-1492-3 (PMC6383231; doi:10.1186/s12909-019-1492-3)
Supplement: Supplementary file 5 — Lecture evaluation, in English and Nepali. (PDF 156 kb) [file 12909_2019_1492_MOESM5_ESM.pdf]

LECTURE EVALUATION

1) My level of training
   - ANM
   - CMA
   - MBBS
   - Nurse
   - HA
   - MD-GP

2) This lecture was easy for me to understand.

Strongly agree — Agree — Disagree — Strongly disagree

3) This lecture was relevant to my work.

Strongly agree — Agree — Disagree — Strongly disagree

4) I can identify one change I will make in my work because of this lecture.

Yes — No — If yes, what change? ______________________________________

5) I asked a question, answered a question, or spoke in a discussion during this lecture.

Yes — No

Comments:

१) योग्यता
   - ANM
   - CMA
   - MBBS
   - Nurse
   - HA
   - MD-GP

२) यो कक्षा मेरो लागी बुझ्न सजिलो थियो।

पूर्णरुपले सहमत छु — सहमत छु — असहमत छु — पूर्णरुपले असहमत छु

३) यो कक्षा मेरो लागि उपायोगी थियो।

पूर्णरुपले सहमत छु — सहमत छु — असहमत छु — पूर्णरुपले असहमत छु

४) यो कक्षपछी मैले मेरो काममा केहि परिवर्तन ल्याउन सक्छु।

हो — होइन — हो भने एउटा उदाहरण दिनुहोस् ___________________________________

५) यो कक्षमा मैले कुनै प्रश्न सोधेँ, वा कुनै प्रश्नको उत्तर दिएँ वा छलफल मा सहभागी भएँ ।

हो — होइन

६) कुनै सल्लाह अथवा सुझाव छ भने दिनुहोस्:|
